# Carrageenan catabolism is encoded by a complex regulon in marine heterotrophic bacteria

**DOI:** 10.1038/s41467-017-01832-6

**Published:** 2017-11-22

**Authors:** Elizabeth Ficko-Blean, Aurélie Préchoux, François Thomas, Tatiana Rochat, Robert Larocque, Yongtao Zhu, Mark Stam, Sabine Génicot, Murielle Jam, Alexandra Calteau, Benjamin Viart, David Ropartz, David Pérez-Pascual, Gaëlle Correc, Maria Matard-Mann, Keith A. Stubbs, Hélène Rogniaux, Alexandra Jeudy, Tristan Barbeyron, Claudine Médigue, Mirjam Czjzek, David Vallenet, Mark J. McBride, Eric Duchaud, Gurvan Michel

**Affiliations:** 1 Sorbonne Universités, UPMC Univ Paris 06, CNRS, UMR 8227, Integrative Biology of Marine Models, Station Biologique de Roscoff, CS 90074 Roscoff, Bretagne France; 2 VIM, INRA, Université Paris-Saclay, 78350 Jouy-en-Josas, France; 3 Department of Biological Sciences, University of Wisconsin-Milwaukee, 53201 Milwaukee, WI USA; 4 UMR 8030, CNRS, Université Évry-Val-d’Essonne, CEA, Institut de Génomique - Genoscope, Laboratoire d’Analyses Bioinformatiques pour la Génomique et le Métabolisme, F-91000 Évry, France; 5 INRA, UR1268 Biopolymers Interactions Assemblies, F-44316 Nantes, France; 6 School of Molecular Sciences, The University of Western Australia, Crawley, WA 6009 Australia

## Abstract

Macroalgae contribute substantially to primary production in coastal ecosystems. Their biomass, mainly consisting of polysaccharides, is cycled into the environment by marine heterotrophic bacteria using largely uncharacterized mechanisms. Here we describe the complete catabolic pathway for carrageenans, major cell wall polysaccharides of red macroalgae, in the marine heterotrophic bacterium *Zobellia galactanivorans*. Carrageenan catabolism relies on a multifaceted carrageenan-induced regulon, including a non-canonical polysaccharide utilization locus (PUL) and genes distal to the PUL, including a *susCD*-like pair. The carrageenan utilization system is well conserved in marine *Bacteroidetes* but modified in other phyla of marine heterotrophic bacteria. The core system is completed by additional functions that might be assumed by non-orthologous genes in different species. This complex genetic structure may be the result of multiple evolutionary events including gene duplications and horizontal gene transfers. These results allow for an extension on the definition of bacterial PUL-mediated polysaccharide digestion.

## Abstract (web summary)

Carrageenans, major cell wall polysaccharides of red macroalgae, are metabolised by marine heterotrophic bacteria through unclear mechanisms. Here, the authors identify an unusual polysaccharide-utilization locus encoding carrageenan catabolism in a marine bacterium, and characterise the complete pathway.

## Introduction

Carrageenans, alongside agars, are the main cell wall polysaccharides of red macroalgae and play vital roles in the development and physiology of these photosynthetic eukaryotes. These complex polymers consists of d-galactose based units alternatively linked by β-1,4 and α-1,3 linkages. The β-linked unit is either a d-galactose-6-sulfate or a 3,6-anhydro-d-galactose, a bicyclic sugar unique to red macroalgae^1^. Carrageenan structures are further modified by the presence of various substituents (sulfate, methyl and pyruvate groups). For more than 70 years, carrageenans have been widely used as ingredients in food, personal care, and cosmetic industries due to their gelling and emulsifying properties. In addition, these polysaccharides and their derived oligosaccharides have multiple biological properties and are promising molecules for blue biotechnology^2^. In marine ecosystems carrageenans constitute a huge biomass and thus a precious carbon source for marine heterotrophic bacteria (MHB). However, carrageenan catabolism is largely uncharacterized and only a few enzymes specific for these sulfated galactans are known^3–7^.

*Bacteroidetes* are considered key recyclers of marine polysaccharides^8–10^ and notably of carrageenans^2^. Bacteria from this phylum are particularly suitable models for efficiently characterizing complete carbohydrate degradation pathways. Indeed, they have developed multi-component protein systems tailored for sensing, binding, transporting, and degrading specific glycans, as described for the starch-utilization system (Sus)^11,12^. The genes encoding these proteins are adjacent and co-regulated; these regions are referred to as Polysaccharide Utilization Loci (PUL (singular) or PULs (plural)). Tandem *susD*-like and *susC*-like genes, which encode a carbohydrate-binding lipoprotein and a TonB-dependent transporter (TBDT) respectively, are considered as a hallmark of PULs^13^, and the presence of these *susCD*-like gene pairs is used to identify PULs in *Bacteroidetes* genomes^14^. Several PULs have been extensively characterized in human intestinal *Bacteroidetes* ^15–18^, describing the complex molecular mechanisms behind the glycoside hydrolase (GH) enzymes and carbohydrate-binding proteins involved in the digestion of common dietary polysaccharides. Some previous studies have described the partial characterization of PULs targeting marine polysaccharides, for instance, alginate-specific PULs were shown to be genuine operons and to encode the enzymes responsible for the bioconversion of alginate into 2-keto-3-deoxy-6-phosphogluconate^19^. The transcription of a PUL in *Bacteroides plebeius* was shown to be induced by porphyran. The PUL-encoded enzymes responsible for the initial endolytic degradation of this sulfated agar (family GH16 and GH86 β-porphyranases) were biochemically and structurally characterized^20^. However, such studies, focusing on macroalgal polysaccharides, have not yet reached the level of integrative characterization observed in the field of PULs from human gut *Bacteroidetes*.

The marine *Bacteroidetes Zobellia galactanivorans* Dsij^T^, originally isolated from a healthy red macroalga, is a bacterial model for the bioconversion of algal polysaccharides^10^. Notably, this bacterium can utilize kappa-carrageenan (KC), iota-carrageenan (IC), and lambda-carrageenan as sole carbon sources and its kappa-carrageenase (CgkA, CAZy family GH16, http://www.cazy.org/^21^) and iota-carrageenases (CgiA1, CgiA2, and CgiA3, CAZy family GH82) have been studied^22–24^.

Here we describe in *Z. galactanivorans* the discovery and the integrative characterization of the complete pathway for utilization of the kappa family carrageenans (containing 3,6-anhydro-d-galactose units, mainly kappa-, iota- and beta-carrageenans^25^), which is carried out by a complex regulon including a dedicated atypical PUL, lacking the *susCD*-like pair, and carrageenan-induced loner genes including distal *susCD*-like pairs. The core of this complex system is conserved in MHB from different phyla. These carrageenan utilization systems appear to display a remarkable plasticity, likely resulting from diverse evolutionary events such as horizontal gene transfers and gene duplications.

## Results

### The ZGAL_3145-3159 cluster encodes carrageenan-specific enzymes

**Fig. 1:**
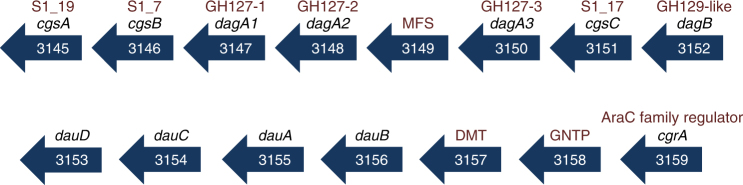
*Z. galactanivorans* PUL involved in carrageenan catabolism. Genes are annotated according to their CAZy family (GH127, GH129-like), sulfatase subfamily (S1_19, S1_7, and S1_17) or enzymatic activity. The names of the genes and their functions are given in Table 1. The acronyms for the gene names are as follows: *cgs*, CarraGeenan Sulfatase; *dag*, d-3,6-AnhydroGalactosidase; *dau*, d-3,6-Anhydrogalactose Utilization; *cgr*, CarraGeenan Regulator

The annotation of the genome sequence of *Z. galactanivorans* ^10^ revealed a cluster of genes encoding three GH127 enzymes (ZGAL_3147, ZGAL_3148, and ZGAL_3150), one enzyme assigned to the GH129 family^21^ (ZGAL_3152), and three sulfatases belonging to different S1 subfamilies (ZGAL_3145, ZGAL_3146, and ZGAL_3151 belonging to the subfamilies S1_19, S1_7, and S1_17, respectively, http://abims.sb-roscoff.fr/sulfatlas/^26^), suggesting this gene cluster could be a PUL specific for a sulfated algal polysaccharide (Fig. 1). This 15-gene locus notably encodes 4 additional carbohydrate-related enzymes (ZGAL_3153-3156), three cytoplasmic membrane transporters and an *araC* family transcription factor (ZGAL_3159), but does not include a *susCD*-like gene pair. The GHs have distant homologs in enteric bacteria: BT1003 (*Bt*GH127) from *Bacteroides thetaiotaomicron* is a 3-C-carboxy-5-deoxy-l-xylose (aceric acid)-hydrolase involved in rhamnogalacturonan-II depolymerization^18^; *Bl*GH127 from *Bifidobacterium longum* is an exo-β-l-arabinofuranosidase that acts on the plant glycoproteins extensins^27^; NagBb (*Bb*GH129) from *Bifidobacterium bifidum* hydrolyzes alpha-linked N-acetyl-d-galactosamine from intestinal mucin^28^. To the best of our knowledge L-aceric acid, β-L-arabinofuranose and α-N-acetyl-d-galactosamine are not known components of red algae^29^, suggesting that the GHs from *Z. galactanivorans* may have new substrate specificities (Supplementary Discussion). To test these hypotheses, we first cloned and overproduced the 11 enzyme-coding genes of this cluster in *Escherichia coli* BL21(DE3). The resulting recombinant proteins were all soluble and were purified by affinity chromatography (Supplementary Fig. 1), allowing examination of their activities.

**Fig. 2:**
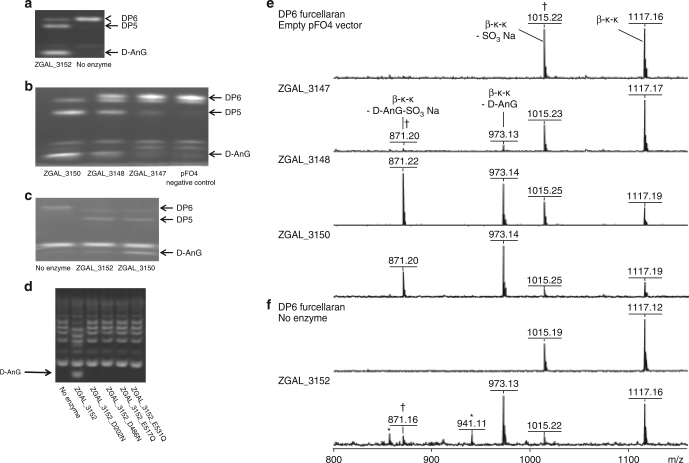
Biochemical characterization of the GH127 and GH129-like enzymes. GH127 enzymes (DagA1, DagA2, DagA3; ZGAL_3147, ZGAL_3148, ZGAL_3150) and GH129-like enzymes (DagB, ZGAL_3152). **a** FACE gel depicting the reaction products after furcellaran (beta-carrageenan) oligosaccharides were treated with pure ZGAL_3152. **b** FACE gel depicting the reaction products after furcellaran oligosaccharides were treated with soluble lysate of *E. coli* BL21 (DE3) that were transformed with the pFO4 vector alone (negative control) or with the genes of interest cloned into the pFO4 vector **c** FACE gels depicting the reaction products after furcellaran oligosaccharides were treated with IMAC-purified ZGAL_3152 and ZGAL_3150. **d** FACE gels depicting the reaction products after furcellaran oligosaccharides were treated with ZGAL_3152 and four conservative active site mutants. **e** MALDI MS spectra of the DP6 beta-kappa-kappa oligosaccharide obtained in negative ionization mode after incubation with soluble lysates of BL21(DE3) cells that were transformed with the pFO4 vector only and ZGAL_3147, ZGAL_3148, ZGAL3150. **f** Spectrum obtained for DP6 beta-kappa-kappa oligosaccharide incubated with no enzyme and the spectrum of the same sample after treatment with IMAC purified ZGAL_3152. Fragments annotated with a † correspond to a sulfate loss induced by the ionization process. Peaks annotated with a * correspond to HEPES adducts on matrix clusters. DP stands for degree of polymerization

**Fig. 3:**
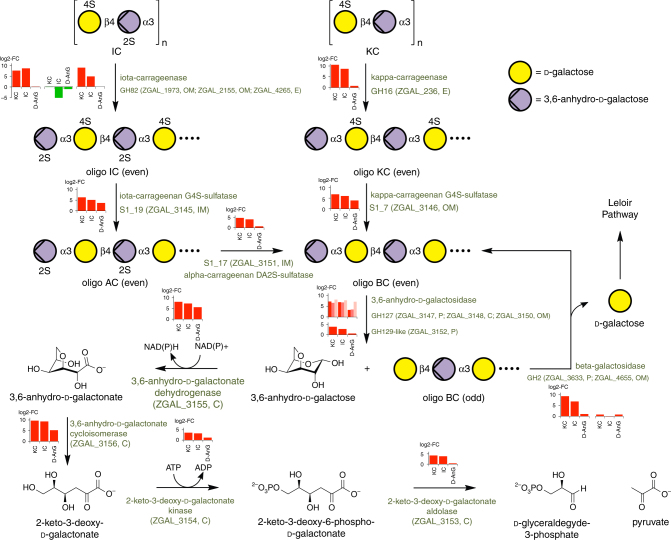
Kappa-carragenan and iota-carrageenan catabolic pathways in *Z. galactanivorans*. Enzymes performing each reaction step are mentioned, together with their encoding genes. For each step, bar plots depict the relative expression of the corresponding gene(s) in kappa-carrageenan (KC), iota-carrageenan (IC), or 3,6-anhydro-d-galactose (D-AnG) compared to d-galactose, as measured by RNA-seq (values are mean of log2-fold change, *n* = 3). Predicted enzyme localization is shown next to the gene number: OM, outer membrane; IM, inner membrane; E, extracellular; C, cytoplasmic; P, periplasm. AC stands for alpha-carrageenan and BC for beta-carrageenan. In this schematic, alpha- and beta-carrabiose motifs are considered as intermediary compounds. Nonetheless, alpha- and beta-carrageenans can be naturally found in some red algal species and the catabolic pathway described here is also valid for these natural carrageenans

In vitro assays of the GH127 and GH129-like enzymes showed no activity on poly- or oligo-saccharides of agarose (uncharged substrate), agars, porphyrans, KC or IC (sulfated substrates). However, the presence of sulfatases in the gene cluster suggested that these GHs could act after the sulfatases and thus require de-sulfated substrates. Since we had already tested agarose, we searched for a natural source of de-sulfated carrageenans. *Furcellaria lumbricalis* is a red macroalga with beta-carrageenan (BC) in its cell wall, primarily composed of kappa-carrabiose and uncharged beta-carrabiose motifs^30^. This hybrid polysaccharide is commonly referred to as furcellaran. No GH activity was detected on furcellaran using fluorophore-assisted carbohydrate electrophoresis (FACE)^31^. In order to produce oligosaccharides, furcellaran was treated with the kappa-carrageenase from *Pseudoalteromonas carrageenovora* which cleaves the β-1,4 bond within kappa-carrabiose motifs in an endolytic manner^3^. The product oligosaccharides have a neutral 3,6-anhydro-d-galactose (D-AnG) on the non-reducing end and a d-galactose-4-sulfate on the reducing end. These oligosaccharides were purified by size-exclusion chromatography and the fraction containing a majority of hexasaccharides was tested with the GHs (Fig. 2). All the GHs showed activity on the furcellaran hexasaccharide, as demonstrated using FACE. Nonetheless, there appears to be some as yet undetermined differences in specificity between the three GH127 enzymes as the FACE gel shows different intensities and banding patterns (Fig. 2b). ZGAL_3150 (GH127-3) and ZGAL_3152 (GH129-like) were the most active enzymes and appear indistinguishable biochemically based on FACE patterns (Fig. 2c). MALDI-TOF-MS analysis of these enzymatic digests indicated the release of the terminal neutral monosaccharide D-AnG and of a pentasaccharide (Fig. 2e). Thus, these enzymes are exo-lytic α-1,3-(3,6-anhydro)-d-galactosidases which cleave the α-1,3 linkage between D-AnG and d-galactose on the non-reducing end, releasing D-AnG and odd-DP (degree of polymerization) oligocarrageenans (Fig. 3). Overall, this describes a novel enzymatic activity, long predicted to be present in nature, but for the first time described here in two families of non-homologous enzymes. The genes of these new GHs have been named: *dagA*1 (ZGAL_3147 encoding GH127-1), *dagA*2 (ZGAL_3148 encoding GH127-2), *dagA*3 (ZGAL_3150 encoding GH127-3), and *dagB* (ZGAL_3152 encoding GH129-like). Furthermore, this discovery strongly supports that the ZGAL_3145-3159 gene cluster is carrageenan-specific.

ZGAL_3145 (S1_19), ZGAL_3146 (S1_7), and ZGAL_3151 (S1_17) were first shown to be active sulfatases using the artificial substrate 4-methylumbelliferyl sulfate. A combination of anion-exchange chromatography (HPLC) and ^1^H-NMR was used to determine the natural substrates and the regioselectivity of these sulfatases (Supplementary Discussion). As predicted, all these sulfatases were active on carrageenans (Supplementary Fig. 2A, B). ZGAL_3146 (gene named *cgsB*1) is active on kappa-carrabiose motifs, removing the 4-linked sulfate group from d-galactose to generate beta-carrabiose motifs (Supplementary Fig. 2C). ZGAL_3145 (*cgsA*) removes the 4-linked sulfate group from the galactose moiety of iota-carrabiose motifs, generating alpha-carrabiose motifs (Supplementary Figs. 3, 4). ZGAL_3151 (*cgsC*) acts subsequently on the alpha-carrabiose motifs, removing the 2-linked sulfate group from D-AnG to generate beta-carrabiose motifs (Supplementary Fig. 4). To the best of our knowledge, this is the first time this sulfatase activity has been demonstrated.

The original annotations of the four remaining enzymes were not obviously connected to carrageenan: 2-dehydro-3-deoxy-6-phosphogalactonate aldolase (ZGAL_3153), 2-dehydro-3-deoxygalactonokinase (ZGAL_3154), lactaldehyde dehydrogenase (ZGAL_3155), and aldonic acid dehydratase (ZGAL_3156). However, recent discovery of two enzymes from *Vibrio* sp. *EJY3* that convert 3,6-anhydro-l-galactose (agar component) into 2-dehydro-3-deoxygalactonate^32^ provided new insights. Indeed, *Vibrio* 3,6-anhydro-l-galactose dehydrogenase (VEJY3_09240) and 3,6-anhydro-l-galactonate cycloisomerase (VEJY3_09370) are distantly related to ZGAL_3155 and ZGAL_3156 (35% and 31% sequence identity, respectively). We thus hypothesized that ZGAL_3155 and ZGAL_3156 could catalyze similar reactions but on the D enantiomer of 3,6-anhydrogalactose. As predicted, ZGAL_3155 oxidizes D-AnG into 3,6-anhydro-d-galactonate in the presence of NAD^+^ and NADP^+^, with a 5–6-fold preference for NAD^+^ (Supplementary Fig. 5A, B). ZGAL_3155 is inactive on d-galactose and is therefore a specific 3,6-anhydro-d-galactose dehydrogenase (gene named *dauA*). The resulting 3,6-anhydro-d-galactonate is converted to 2-keto-3-deoxy-d-galactonate by ZGAL_3156, as measured through the thiobarbituric acid (TBA) assay (Supplementary Fig. 5C). ZGAL_3156 did not demonstrate any activity on D-AnG alone (Supplementary Fig. 5D) and is thus a 3,6-anhydro-d-galactonate cycloisomerase (*dauB*). In the presence of ATP, ZGAL_3154 phosphorylated the 2-keto-3-deoxy-d-galactonate to 2-keto-3-deoxy-6-phospho-d-galactonate. The activity of this 2-keto-3-deoxy-d-galactonate kinase (*dauC*) was indirectly measured as a function of the oxidation of NADH (Supplementary Fig. 5E). Finally, ZGAL_3153 catalyzed the conversion of 2-keto-3-deoxy-6-phospho-d-galactonate into d-glyceraldehyde-3-phosphate and pyruvate. The activity of this 2-keto-3-deoxy-d-galactonate aldolase (*dauD*) was measured both in the forward and reverse direction using the TBA assay (Supplementary Fig. 5F, G). In parallel, and in support of our findings, Lee et al.^33^ recently biochemically characterized homologs of these four enzymes in other marine bacteria.

### Crystal structure of two 3,6-anhydro-d-galactose-related enzymes

Most recombinant proteins were put into crystal trials in order to deepen our understanding of the structure/function relationship of these new enzymes. We were successful in solving the structures of the first α-1,3-(3,6-anhydro)-d-galactosidase (DagB, ZGAL_3152, GH129-like) and of the first 3,6-anhydro-d-galactonate cycloisomerase (DauB, ZGAL_3156) (pdb id 5opq and 5olc, Supplementary Table 1).

**Fig. 4:**
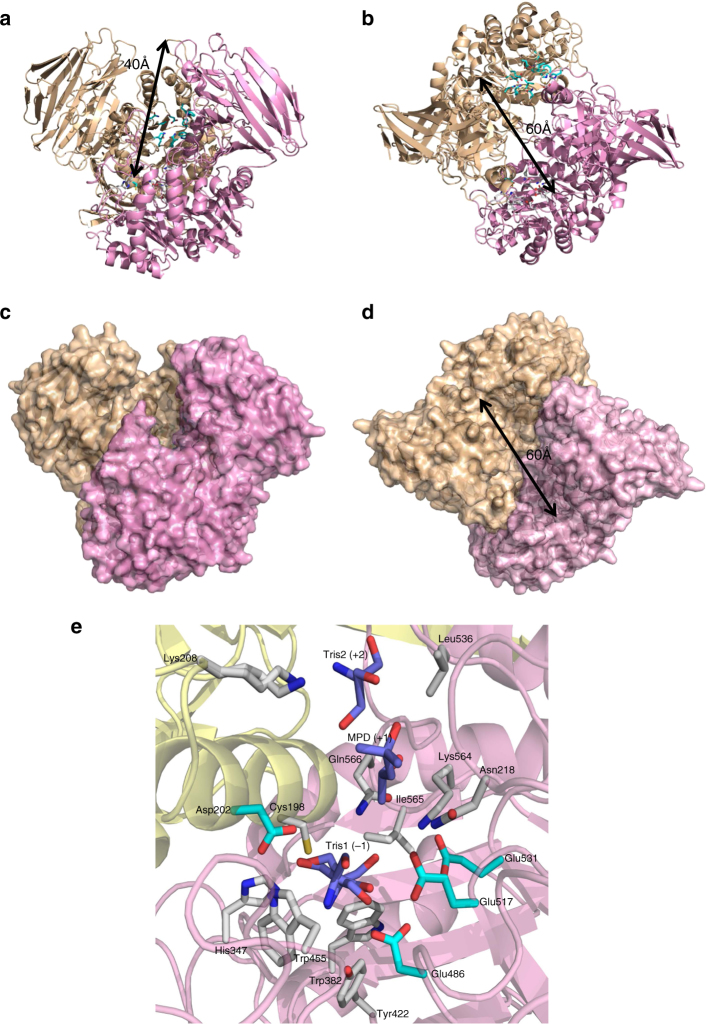
X-ray crystal structure of the α-1,3-(3,6-anhydro)-d-galactosidase DagB. **a** Secondary structure representation of DagB (ZGAL_3152, GH129-like) showing the depth of the active site crevasse for ZGAL_3152 (yellow and pink secondary structure representation for each monomer, pdb id 5opq). Active site residues, colored by element, are shown in light blue for one monomer and white for the other. **b** Secondary structure representation showing the width of the active site crevasse. **c** Surface structure representation of the model orientation shown in **a**. **d** Surface structure representation of the model orientation shown in B. **e** Active site substructure of the α-1,3-(3,6-anhydro)-d-galactosidase ZGAL_3152 (DagB). Three putative subsites are shown as represented by bound Tris1 (subsite –1), MPD (subsite +1) and Tris2 (subsite +2). Four potential catalytically active acidic residues are shown in light blue belonging to two different monomers of ZGAL_3152. Their site-directed mutagenesis leads to inactive enzymes. The amino acids, colored by element, interact with the Tris and MDP molecules and are conserved at 80% or more within the closest DagB homologs

ZGAL_3152 forms a homodimer and the monomer has a complex architecture with an N-terminal distorted beta-sandwich domain (Pro35-Asp299), a central TIM barrel (Tyr300-Lys620) and a C-terminal immunoglobulin-like domain (Glu621-Asp693) (Fig. 4a–d, Supplementary Fig. 6). The dimeric interface is mainly formed by the interaction of long loops from the TIM-barrel (342–357 and 430–459) with the two helices protruding from the N-terminal domain (η3 and α2) of the other monomer. The dimer is also stabilized by the swapping of the C-terminal strand β36. There are two predicted active sites at either side of the base of an impressive crevasse that is 60 Å long and 40 Å deep (Fig. 4a, b). Numerous trials for obtaining the structure of ZGAL_3152 complexes were performed, but they were unsuccessful; however, buffer molecules found in the active site of each ZGAL_3152 monomer likely mimic monosaccharide units. In chain C, a Tris, an MDP and a second Tris are bound in the potential subsites −1, +1, and +2, respectively (Fig. 4e). The residues interacting with these buffer molecules (Cys198, Lys208, Asn218, His347, Trp455, Leu536, and Gln566) are well conserved in ZGAL_3152 homologs (Supplementary Fig. 7), suggesting their implication in substrate recognition. Four acidic residues are candidate catalytic residues. Three are located in one monomer from the homodimer (Asp486, Glu517, and Glu531), while Asp202 originates from helix α2 of the second monomer. Asp202, Asp486, and Glu517 are strictly conserved. Site-directed mutagenesis of the four candidates yielded soluble inactive enzymes (Fig. 2d); these residues are thus involved in the catalytic machinery, although the structure of a substrate-enzyme complex would be needed to determine their respective roles. Surprisingly, among the two catalytic residues identified in the GH129 alpha-N-acetylgalactosaminidase NagBb^28^, only Asp435 is conserved in ZGAL_3152 homologs (Asp486 in ZGAL_3152, Supplementary Fig. 7). ZGAL_3152 and NagBb display only 16% sequence identity and therefore, considering this extreme sequence divergence and the non-conservation of the catalytic residues, we propose that ZGAL_3152 homologs do not belong to the GH129 family but rather constitute a new GH family.

**Fig. 5:**
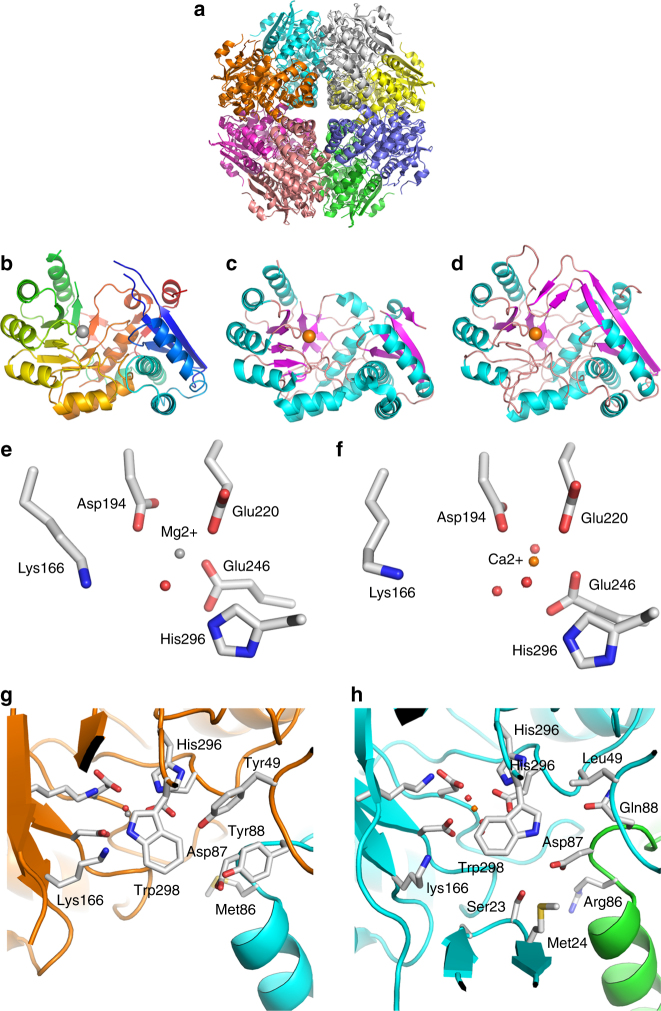
X-ray crystal structure of the 3,6-anhydro-d-galactonate cycloisomerase DauB. **a** Cartoon representation of the octamer of DauB (ZGAL_3156; pdb id 5olc), each chain being colored with a different color. **b** Cartoon representation of the monomer of ZGAL_3156 (chain A) colored according to a blue-red gradient from N- to C-terminal. **c** Cartoon representation of the low-resolution structure (2.0 Å) of the monomer of the d-galactaro-1,4-lactone cycloisomerase *At*GCI from *Agrobacterium tumefaciens* (pdb id 4ggb).The α-helices and β-strands are colored in magenta and cyan, respectively. **d** Cartoon representation of the high-resolution structure (1.6 Å) of the monomer of *At*GCI (pdb id 4hpn) with the entire lid domain visible. **e** and **f** Zoom view of the catalytic machinery of ZGAL_3156 and *At*GCI (pdb id 4hpn), respectively. **g** and **h** Zoom view of the substrate binding pocket of ZGAL_3156 and *At*GCI (pdb id 4hpn), respectively. The chains **h** (in orange) and **b** (cyan) of ZGAL_3156 are shown **g**. Two equivalent chains of the *At*GCI (Cyan and green, respectively) are shown **h**. The crystal corresponding to 4hpn contains only one molecule per asymmetric unit and the biological octamer of *At*GCI was thus generated by the crystallographic symmetry

ZGAL_3156 folds as a (β/α)_7_β TIM-barrel (amino acids 137–340) with an α/β lid domain (amino acids1–136 and 341–377). The crystal structure reveals an octamer, not uncommon in the enolase superfamily (Fig. 5a, Supplementary Fig. 8). A size-exclusion column analysis confirmed that ZGAL_3156 constitutes an octamer in solution (Supplementary Fig. 9). In the enolase superfamily, the active site is located at the interface of the two domains, the lid domain shielding the catalytic machinery from bulk solvent. Two disordered regions (17–26 and 138–143) were not modeled. They are close spatially and constitute the tip of the lid domain (Fig. 5b). Equivalent regions are similarly disordered in the low-resolution structure of the d-galactaro-1,4-lactone cycloisomerase *At*GCI from *Agrobacterium tumefaciens* (41% identity, PDB: 4ggb, Fig. 5c), speaking to the flexibility of the lid region in these enzymes^34^. The residues involved in the coordination of the catalytic cation (Asp194, Glu220, and Glu246) and the predicted general base (Lys166) and acid (His296) of *At*GCI are strictly conserved between *At*GCI and ZGAL_3156 and have similar spatial orientations (Fig. 5e, f). This cation was modeled as an Mg^2+^ in ZGAL_3156 and as a Ca^2+^ in *At*GCI. The residues shaping the substrate-binding pocket in *At*GCI originate from the TIM-barrel and the lid domain of one monomer and from the loop between helices α2 and α3 of the neighboring monomer (Fig. 5h). While Asp87 and Trp298 are conserved in both enzymes, the other residues are substituted and are thus likely involved in 3,6-anhydro-d-galactonate recognition (Fig. 5g).

### PUL-encoded genes are essential for the in vivo utilization of carrageenans

**Fig. 6:**
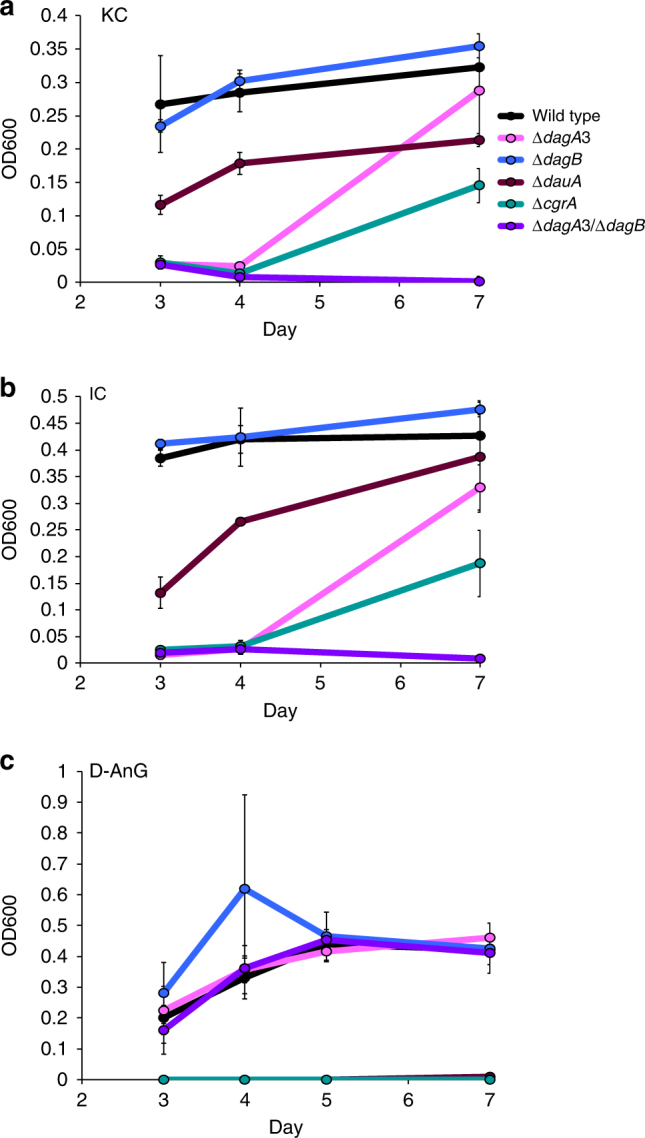
Average growth of wild-type *Z. galactanivorans* and deletion mutants. Growth on **a** kappa-carrageenan, **b** iota-carrageenan and **c** 3,6-anhydro-d-galactose (D-AnG) as sole carbon source over a 7-day period. Error bars represent standard error of the mean between three replicates. Legend: *dagA*3, α-1,3-(3,6-anhydro)-d-galactosidase (*zgal_3150*, *gh127*); *dagB*, α-1,3-(3,6-anhydro)-d-galactosidase (*zgal_3152*, *gh129*-like); *dauA*, 3,6-anhydro-d-galactose dehydrogenase (*zgal_3155*); *crgA*, araC family transcription factor (*zgal_3159*)

To investigate in vivo gene function in *Z. galactanivorans*, we recently developed a genetic technique to construct deletion mutants in this bacterium^35^. Four single deletion mutants [Δ*dagA3* (Δ*zgal_3150*, Δ*gh127-3*), Δ*dagB* (Δ*zgal_3152*, Δ*gh129*-like), Δ*dauA* (Δ*zgal_3155*, Δ*3,6-anhydro-D-galactose dehydrogenase*), Δ*cgrA* (Δ*zgal*_*3159*, Δ*araC family regulator*)] and a double deletion mutant (Δ*dagA3*/Δ*dagB*) were constructed. They all grew comparably to the wild-type strain in Zobell medium and minimum medium supplemented with agar or galactose (Supplementary Fig. 10A, B). The Δ*dagB* mutant had little effect on growth relative to wild type in minimal media containing either KC or IC; however, the Δ*dagA*3 mutant showed a significant growth delay and the double Δ*dagA*3/Δ*dagB* mutant abolished growth altogether, confirming the importance of these α-1,3-(3,6-anhydro)-d-galactosidases in carrageenan degradation (Fig. 6a, b). This sharply contrasts with the biochemical characterization of ZGAL_3150 (DagA3, GH127-3) and ZGAL_3152 (DagB, GH129-like), where there was no discernible difference in specificity (Fig. 2c). We attribute the difference in deletion effect may be due to either an unknown difference in specificity between the enzymes or different cellular localizations of the enzymes. Subtle differences between the GH127 and GH129-like enzymes would not be surprising considering the complex, hybrid structure of carrageenans whose regular structures are masked by multiple substituents (e.g., sulfate, methyl, pyruvate). For instance, such variations in substrate specificities are known in GH16 β-agarases and β-porphyranases which act on other red algal sulfated galactans^36^. Bioinformatic analyzes predict that ZGAL_3150 and ZGAL_3152 are anchored into the outer membrane and secreted in the periplasm, respectively (Fig. 3). The more likely scenario is that ZGAL_3150 is oriented toward the periplasm. Thus, both enzymes would be localized within the periplasm in order to sequentially degrade carrageenan oligosaccharides to produce the monosaccharide D-AnG for uptake by the bacterium. Finally, this deletion effect indicates that the two remaining GH127 enzymes are unable to compensate for the loss of ZGAL_3150 or ZGAL_3152. The Δ*dauA* mutant did not grow on D-AnG and complementation experiments restored growth on this sugar (Fig. 6c, Supplementary Fig. 10C). This indicates that neither ZGAL_4659 (orthologue of the 3,6-anhydro-l-galactose dehydrogenase VEJY3_09240, 68% identity) nor any other sugar dehydrogenases from *Z. galactanivorans* could degrade D-AnG and that ZGAL_3155 (DauA, 3,6-anhydro-d-galactose dehydrogenase) is essential for D-AnG catabolism. Δ*dauA* had diminished growth on both KC and IC (Fig. 6a, b), suggesting that this mutant is still capable of using the d-galactose units released by carrageenan degradation as a carbon source. The deletion mutant of the AraC family regulator ZGAL_3159 (Δ*cgrA*) showed a significant lag phase and reduced growth on carrageenans (Fig. 6a, b), confirming its importance on the positive regulation of the ZGAL_3145-3159 cluster function. Complementation experiments restored growth of Δ*cgrA* on KC (Supplementary Fig. 10D). In both sets of complementation experiments the growth was improved relative to the wild-type strain (Supplementary Fig. 10C, D). This is likely because the complemented genes are under the *Flavobacterium johnsoniae* OmpA promoter and therefore under less stringent transcriptional control than the carrageenan-specific PUL. Phenotyping of Δ*cgrA* on KC or IC solid media indicates a complex mode of regulation (Supplementary Fig. 10E–L, Supplementary Discussion). Finally, the *cgrA* deletion had no effect on growth with agar, indicating that this transcription factor is specific for carrageenan catabolism (Supplementary Fig. 10A).

### RNA-seq analysis unravels a complex carrageenan-related regulon

RNA-seq expression profiling was performed on *Z. galactanivorans* grown in minimal media containing D-AnG, KC, and IC, relative to the growth on d-galactose (Table 1, Supplementary Data 1–9). The entire carrageenan-specific PUL was strongly upregulated in both KC and IC with the exception of the transcriptional regulator *cgrA* in IC which was at the border of significance. Growth on D-AnG induced 10 genes from the PUL suggesting this monosaccharide, unique to red algae, acts as an effector in regulation. The genes that were induced by D-AnG were specific for oligosaccharide and monosaccharide utilization; no genes were induced for the degradation of carrageenan polymers. Unexpectedly, numerous stress-related proteins were also induced (e.g., small heat shock protein, universal stress protein, peptide methionine sulfoxide reductase). Together with the observation that the growth with D-AnG induced cell aggregation, this suggests that free D-AnG is not frequent in the natural environment of *Z. galactanivorans* and that this sugar is normally only an intracellular degradation intermediate and unlikely to be transported by a specific outer membrane transporter.

**Table 1:** Selection of carrageenan-induced genes in *Z. galactanivorans*

Locus_tag	Description (*gene name*/acronym, family)	KC/D-gal		IC/D-gal		D-AnG/D-gal	
		log2-FC*	*padj*	log2-FC	*padj*	log2-FC	*padj*
ZGAL_181	Sulfatase (*cgsB*2, S1_7)	**8.4**	*7.47E-94*	**8.0**	*5.81E-85*	**5.1**	*3.66E-34*
ZGAL_236	Kappa-carrageenase (*cgkA*, GH16)	**10.6**	*2.68E-163*	**8.7**	*7.26E-110*	0.8	*1*
ZGAL_1973	Iota-carrageenase (*cgiA3*, GH82)	**7.6**	*7.15E-56*	**8.7**	*1.12E-72*	0.1	*1*
ZGAL_3145	Sulfatase (*cgsA*, S1_19)	**6.3**	*1.7E-65*	**5.1**	*1.69E-41*	**3.7**	*3.48E-20*
ZGAL_3146	Sulfatase (*cgsB*1, S1_7)	**6.8**	*1.2E-117*	**6.0**	*1.04E-91*	**3.9**	*1.20E-33*
ZGAL_3147	α-1,3-(3,6-anhydro)-d-galactosidase (*dagA*1, GH127)	**7.2**	*7.4E-135*	**6.9**	*5.12E-125*	**3.3**	*2.67E-25*
ZGAL_3148	α-1,3-(3,6-anhydro)-d-galactosidase (*dagA*2, GH127)	**6.6**	*2.7E-61*	**6.8**	*5.52E-65*	**3.5**	*6.80E-16*
ZGAL_3149	Sugar permease (MFS)	**6.7**	*3.0E-65*	**6.2**	*9.61E-55*	**4.7**	*9.94E-31*
ZGAL_3150	α-1,3-(3,6-anhydro)-d-galactosidase (*dagA*3, GH127)	**8.1**	*2.9E-108*	**7.8**	*3.02E-99*	**7.1**	*3.78E-82*
ZGAL_3151	Sulfatase (*cgsC*, S1_17)	**4.8**	*5.4E-44*	**4.1**	*1.50E-31*	0.7	*1*
ZGAL_3152	α-1,3-(3,6-anhydro)-d-galactosidase (*dagB*, new GH)	**3.9**	*6.5E-23*	**2.8**	*5.72E-11*	0.6	*1*
ZGAL_3153	2-keto-3-deoxy-d-galactonate aldolase (*dauD*)	**4.2**	*9.9E-44*	**3.8**	*2.07E-35*	0.4	*1*
ZGAL_3154	2-keto-3-deoxy-d-galactonate kinase (*dauC*)	**3.6**	*2.4E-30*	**3.3**	*1.84E-25*	1.2	*0.1516*
ZGAL_3155	3,6-anhydro-d-galactose dehydrogenase (*dauA*)	**8.1**	*1.3E-80*	**7.3**	*4.01E-65*	**5.6**	*1.40E-37*
ZGAL_3156	3,6-anhydro-d-galactonate cycloisomerase (*dauB*)	**9.7**	*2.0E-89*	**9.3**	*1.21E-81*	**5.2**	*1.07E-23*
ZGAL_3157	Sugar/H^+^ symporter (DMT)	**8.4**	*5.8E-140*	**8.0**	*3.68E-125*	**7.0**	*3.85E-95*
ZGAL_3158	High-affinity sugar transporter	**4.3**	*8.1E-39*	**3.0**	*4.15E-17*	**4.1**	*8.61E-34*
ZGAL_3159	Transcriptional regulator (*cgrA*, AraC family)	**2.3**	*1.8E-14*	**1.2**	*0.0504*	−0.5	*1*
ZGAL_3580	SusD-like lipoprotein (*cgtB*)	**9.1**	*2.04E-124*	**8.3**	*5.65E-104*	**4.2**	*1.17E-24*
ZGAL_3581	SusC-like TonB-dependent receptor (*cgtA*)	**9.6**	*1.93E-103*	**9.5**	*1.31E-99*	**4.3**	*4.99E-19*
ZGAL_3629	Sulfatase (S1_30)	**8.5**	*8.26E-55*	**4.9**	*2.82E-16*	−0.1	*1*
ZGAL_3630	Sulfatase(S1_28)	**7.1**	*5.54E-48*	**4.3**	*6.27E-16*	0.8	*1*
ZGAL_3631	Polygalacturonase, (GH28)	**7.8**	*1.43E-36*	**3.9**	*1.70E-07*	−0.4	*1*
ZGAL_3632	Polygalacturonase, (GH28)	**7.8**	*8.43E-20*	**4.3**	*1.66E-04*	−0.3	*1*
ZGAL_3633	Beta-galactosidase (GH2)	**9.4**	*1.85E-66*	**6.9**	*2.66E-35*	1.0	*1*
ZGAL_3634	Alpha-l-fucosidase (GH29)	**8.3**	*3.04E-28*	**4.8**	*9.25E-08*	1.8	*1*
ZGAL_3637	SusC-like TonB-dependent transporter (TBDT)	**9.2**	*1.81E-51*	***5.1***	*1.14E-14*	0,7	*1*
ZGAL_3638	SusD-like lipoprotein (SGBP)	**9.9**	*3.92E-53*	***5.3***	*8.26E-14*	−0,1	*1*
ZGAL_4265	Iota-carrageenase (*cgiA*1, GH82)	**8.7**	*2.10E-56*	**4.7**	*6.12E-15*	0.0	*1*

^*^Log2-FC: log2-fold change of KC, IC or D-AnG relative to D-gal as unique carbon source. Boldface type indicates significant changes (FWER 5%). Full data set is available in Supplementary Data 1–9

Numerous genes outside the ZGAL_3145-3159 cluster were strongly upregulated by carrageenans. Significantly, the most induced gene when grown on KC was the kappa-carrageenase *cgkA* (*zgal_236*, *gh16*)^22^; this gene was also heavily induced by growth on IC (Table 1, Supplementary Data 1–9). The iota-carrageenase genes *cgiA*1 (*zgal_4265*, *gh82-1*) and *cgiA*3 (*zgal_1973*, *gh82-3*)^23,24^ were upregulated in both KC and IC. Interestingly, *cgiA*2 (*zgal_2155*, *gh82-2*) was the most expressed iota-carrageenase gene in d-galactose but was downregulated in KC and IC. Thus ZGAL_2155 could act as a constitutive sentinel enzyme involved in the initial degradation step releasing signal oligosaccharides inducing the carrageenolytic system. The wild-type kappa-carrageenase CgkA and iota-carrageenase CgiA1 were previously shown to be extracellular enzymes^22,23^. Thus, the role of these enzymes is probably to generate oligosaccharides for transport by *Z. galactanivorans’* SusCD-like transport system. The family S1–7 sulfatase, ZGAL_181, was also among the genes most induced by carrageenans. The corresponding protein displays 65% identity with ZGAL_3146 (CgsB1, S1_7 sulfatase) which desulfates the alpha-carrabiose into beta-carrabiose motifs, suggesting that ZGAL_181 (gene named *cgsB2*) catalyzes the same reaction. These genes are probably the result of relatively recent gene duplication. Two major players missing in the carrageenan-specific PUL are the archetypal *sus*C-like and *susD*-like genes. The most induced gene when grown on IC was the SusC-like TBDT *zgal_3581*; this gene was also highly induced by growth on KC and to a lesser degree by growth on D-AnG. The adjacent gene encodes a SusD-like lipoprotein (ZGAL_3580) which was also substantially upregulated in all three conditions. This *susCD*-like gene pair, distal to the PUL, is a good candidate for the outer membrane transport system associated with the ZGAL_3145-3159 cluster. This hypothesis is supported by genomic comparative analyzes, mutant phenotyping and biochemical experiments (see below). Finally, a second PUL is strongly induced in both KC and IC but not D-AnG (ZGAL_3629-3638). This gene cluster encodes four glycoside hydrolases (1 GH2, 1 GH29, and 2 GH28), two sulfatases (S1_28 and S1_30 subfamilies) and a SusCD-like pair.

### Characterization of key genes distant from the carrageenan PUL

**Fig. 7:**
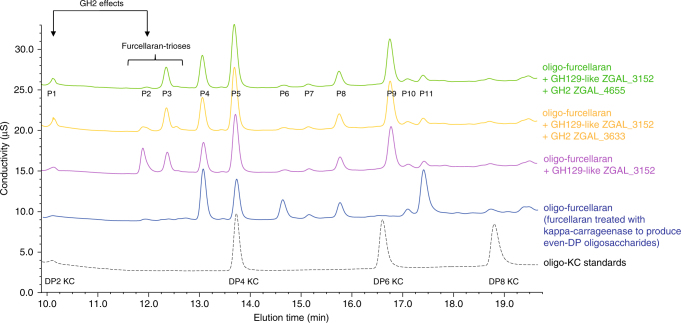
Sequential digestion of furcellaran oligosaccharides by DagB and GH2 enzymes. HPLC results for the ZGAL_3152 (DagB, GH129-like) and GH2 (ZGAL_3633, ZGAL_4655) sequential enzyme digests on furcellaran oligosaccharides. Oligo-kappa-carrageenan standards (DP2, DP4, DP6, and DP8) are shown in black dotted line on the chromatogram. First, oligo-furcellaran (blue line) was treated with ZGAL_3152 (purple line). This reaction was then stopped by heating at 90 °C for 10 min. Following the inactivation of ZGAL_3152 the oligosaccharides were treated with either ZGAL_3633 (orange line) or ZGAL_3655 (green line)

Complete carrageenan catabolism requires the hydrolysis of the beta-1,4-linkage at the non-reducing end of odd-DP oligo-carrageenans produced by the action of the 3,6-anhydro-d-galactosidases. After beta-galactosidase hydrolysis the resulting even-DP oligosaccharides become again substrates for the 3,6-anhydro-d-galactosidases, and so on until the complete degradation into free d-galactose and 3,6-anhydro-d-galactose. We hypothesized that at least one of the 8 predicted GH2 beta-galactosidases encoded in the genome of *Z. galactanivorans* was capable of this activity. ZGAL_3633 stood out as the most probable candidate since its gene expression was induced significantly in both iota- and kappa-carrageenans (Table 1). Thus, we cloned ten GH2 constructs from *Z. galactanivorans*, including ZGAL_3633. All these recombinant enzymes displayed beta-galactosidase activity on an artificial substrate (pNP-beta-d-galactopyranoside, Supplementary Fig. 11). As demonstrated using HPLC (Fig. 7, Supplementary Discussion), two of the GH2s, ZGAL_3633 and ZGAL_4655, are active on odd-DP oligo-carrageenans (furcellaran hydrolyzed by kappa-carrageenase followed by hydrolysis by the 3,6-anhydro-d-galactosidase ZGAL_3152). Both ZGAL_3633 and ZGAL_4655 are constitutively expressed at low levels in *Z. galactanivorans* in minimum medium supplemented with d-galactose (Supplementary Data 1–9); however, ZGAL_4655 is not induced by kappa- and iota-carrageenans, suggesting that ZGAL_3633 is the key carrageenan-specific beta-galactosidase. Thus, *Z. galactanivorans* has all the enzyme activities necessary for the complete degradation of kappa family carrageenans, most of them encoded by the ZGAL_3145-3159 cluster and the others by remote, carrageenan-induced genes (GH16: ZGAL_236; GH82: ZGAL_1973, ZGAL_4265; GH2: ZGAL_3633; S1–7: ZGAL_181).

**Fig. 8:**
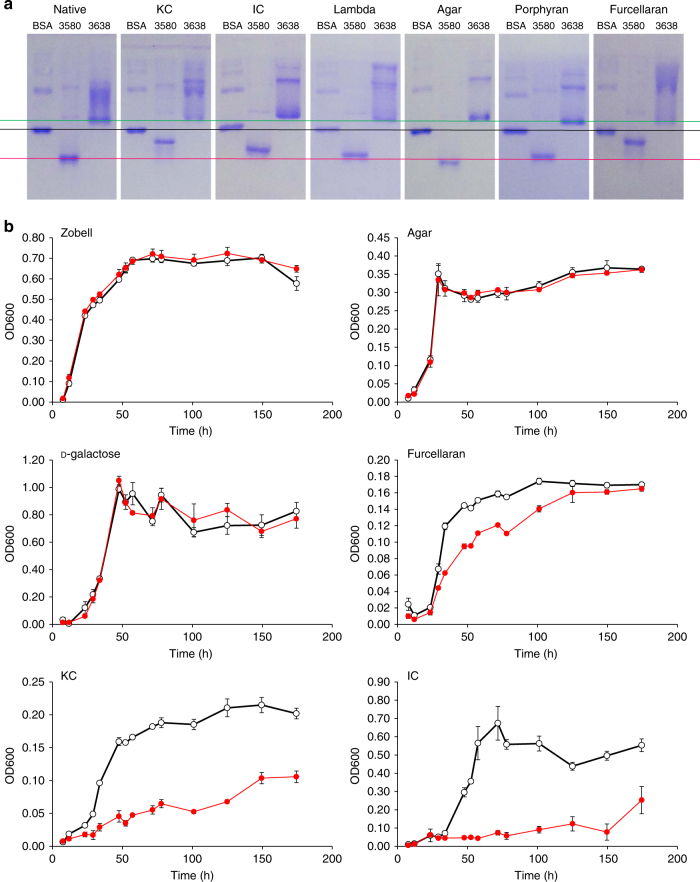
Affinity gel electrophoresis for the SusD-like proteins and phenotyping for the *susCD*-like mutant Δ*cgtA-cgtB*. **a** BSA and recombinant ZGAL_3580 (CgtB, SusD-like) and ZGAL_3638 (SusD-like) migrated at 60 V over 5 h in native-PAGE gels or in gels containing different red algal cell wall polysaccharides. The delay of migration for ZGAL_3580 (red line) and ZGAL_3638 (green line) was compared between the native gels and the polysaccharide containing gels by comparison with BSA (black line). **b** Growth curves for wild-type *Z. galactanivorans* (black curves) and Δ*cgtA-cgtB* (red curves) in rich Zobell media and in minimal media containing different carbohydrate substrates

The carrageenan gene cluster lacks the *susCD*-like gene pair found within canonical PULs; however, the *susCD*-like gene pairs *zgal_3580*/*zgal_3581* and *zgal_3637*/*zgal_3638* are upregulated when grown on iota- and kappa-carrageenans suggesting these are good outer membrane candidates for oligo-carrageenan transport. We succeeded in producing and purifying soluble ZGAL_3580 and ZGAL_3638 (SusD-like proteins) and probed their interaction with red algal cell wall polysaccharides using affinity gel electrophoresis (Fig. 8a). In the native gel without polysaccharide, ZGAL_3580 migrates as a single band while ZGAL_3638 forms a smear. There are changes in the intensity of the different bands corresponding to ZGAL_3638 on all of the polysaccharides tested, suggesting that the polysaccharides may have an effect on the quaternary structure of the protein, but there is no obvious delay in the migration and ZGAL_3638 did not appear to significantly interact with the ligands tested. The absence of ZGAL_3638 specificity for carrageenans and the presence of two putative GH16 beta-porphyranases, ZGAL_3628 (PorD) and ZGAL_3640 (PorE) encoded in the ZGAL_3628-3640 locus suggest that this locus is more likely dedicated to the degradation of another sulfated galactan distinct from carrageenans (e.g., sulfated agars). In contrast, migration of ZGAL_3580 is retarded (as a clear band) by kappa-carrageenan and furcellaran and only slightly by iota carrageenan. No shift is apparent in agar, porphyran or lambda-carrageenan. Therefore, ZGAL_3580 interacts with kappa family carrageenans. Phenotyping experiments on *Z. galactanivorans* deletion mutant Δ*zgal_3580*/Δ*zgal_3581* indicate pronounced inhibition of growth relative to wild type on both KC and IC and moderate inhibition of growth on furcellaran (Fig. 8b). The biochemical (Fig. 8a) and the genetic (Fig. 8b) experiments confirm the affinity of ZGAL_3580 and ZGAL_3581 for family kappa carrageenans, but with a difference in preference depending on the chosen method. However, the genetic approach evaluates the combined properties of ZGAL_3580 and ZGAL_3581, whereas the gel shift assay only characterized ZGAL_3580. This suggests that the TBDT ZGAL_3581 has a strong affinity for iota-carrageenan, which compensates for the reduced affinity of the SusD-like protein ZGAL_3580 for this polysaccharide relative to KC. Overall, these results are consistent with ZGAL_3580/ZGAL_3581 being responsible for carrageenan oligosaccharide import, forming part of the carrageenan regulon and thus acting as the distal *susCD*-like gene pair for the carrageenan PUL. The genes have been named *cgtA* for the SusC-like TBDT (*zgal_3581*) and *cgtB* for the SusD-like lipoprotein (*zgal_3580*).

Based on the biochemical, genetic, and transcriptomic evidence presented here, we have demonstrated for the first time a complete scheme for the catabolism of kappa-, iota- and beta-carrageenans in *Z. galactanivorans*, from the initial action of the GH16 kappa-carrageenase (CgkA)^22^ and GH82 iota-carrageenases (CgiA1-3)^23,24^ to the conversion in four steps of D-AnG into d-glyceraldehyde-3-phosphate and pyruvate (Fig. 3); these latter compounds presumably then enter glycolysis and the citric acid cycle, respectively. The released d-galactose residues are expected to be further converted by the Leloir pathway. The majority of the molecular actors in this pathway and in its regulation are encoded by a discrete genetic locus; however, other key genes are localized remotely on the genome and are part of a carrageenan-induced gene network (Table 1). These genes are thus part of a complex regulon (referred to as the carrageenan utilization system) and the key genes have been named (Table 1).

### Plasticity of carrageenan utilization systems amends the notion of PUL

**Fig. 9:**
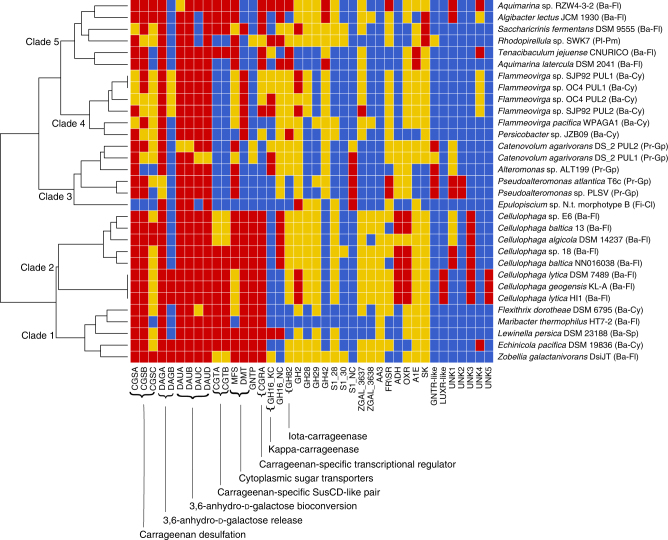
Conservation of the carrageenolytic PUL organization among bacterial genomes. Putative homologs of selected carrageenan-induced genes from *Z. galactanivorans* were found in 29 other bacterial genomes with the conservation of these genes evaluated by a heatmap. Based on these conservation profiles, the bacterial species cluster into 5 main clades. Red coloring indicates the genes are conserved in a PUL, yellow indicates the genes are conserved elsewhere in the genome and blue indicates the genes are absent in the genome. Gene names for the *Z. galactanivorans* carrageenan-utilization locus are found in Table 1 and the legend of Fig. 1. Abbreviations: CGTA, CarraGeenan Transport A (SusC-like, TonB-dependent transporter); CGTB, CarraGeenan Transport B (SusD-like lipoprotein); GH, glycoside hydrolase; S1, family 1 sulfatase; AA3, auxiliary activity 3; FR/SR, fumarate reductase/succinate dehydrogenase; ADH, aldehyde dehydrogenase; OXR, isoquinoline 1-oxidoreductase alpha subunit; A1E, aldose 1-epimerase; SK, sugar kinase; GNTR-like and LUXR-like, transcription factor families GNTR and LUXR; UNK, unknown function. The phylum and class are indicated beside each organism in parentheses: Ba, *Bacteroidetes*; Pl, *Planctomycetes*; Pr, *Proteobacteria*; Fi, *Firmicutes*; Fl, *Flavobacteriia*; Pm, *Planctomycetia*; Cy, *Cytophagia*; Gp, *Gammaproteobacteria*; Cl, *Clostridia*; Sp, *Sphingobacteriia*

We searched the Genbank database for bacteria possessing a potential carrageenan-specific PUL, using BlastP with key enzymes as queries (ZGAL_3150 (DagA3, GH127-3), ZGAL_3155 (DauA, 3,6-anhydro-d-galactose dehydrogenase)). After manual verification of each genomic region, we identified 29 species with a homologous carrageenan-specific PUL (including *Tenacibaculum jejuense* whose genome sequence has been sequenced by Eric Duchaud’s group and deposited at EMBL in the context of this study). These bacteria belong to four phyla: *Bacteroidetes*, *Proteobacteria, Planctomycetes*, and *Firmicutes*. All these microorganisms originate from marine ecosystems: mostly free-living bacteria isolated from seawater, marine sediments or isolated at the surface of macroalgae, but also gut bacteria from animals feeding on macroalgae (surgeon fish, sea urchin, and abalone). The limits of each PUL were manually refined and clusters of orthologous genes from this PUL were subsequently determined (Supplementary Data 10, 11, Supplementary Fig. 12). Homologs of selected carrageenan-induced genes from *Z. galactanivorans* were also searched in the 29 bacterial genomes (Supplementary Data 10, 11) with the conservation of these genes evaluated by a Heatmap; based on these conservation profiles, the bacterial species clustered into 5 main clades (Fig. 9). Clades 1 and 2 include only *Bacteroidetes* (from different classes) and their PUL organizations are the most similar to that of *Z. galactanivorans*. Strikingly, several carrageenan-induced genes remote from the *Z. galactanivorans* carrageenan-specific PUL are found within the carrageenan-specific PULs of other *Bacteroidetes*. This is notably the case of ZGAL_3581 (*cgtA*) and ZGAL_3580 (*cgtB*), which is consistent with the function in *Z. galactanivorans* of this SusCD-like pair in the import of carrageenan degradation-products. *Z. galactanivorans* possesses two S1_7 sulfatases (65% sequence identity), one located in the carrageenan PUL (ZGAL_3146) and the other distal to the PUL (ZGAL_181) but forming part of the carrageenan regulon (Table 1). In contrast, the PULs of several *Bacteroidetes* species contains both orthologous genes of the S1_7 sulfatases (Supplementary Fig. 12), consistent with the hypothesis of recent gene duplication. The GH127 genes are likely another example of gene duplication, since their number varies from 1 to 3 paralogous genes depending on the species. Other *Bacteroidetes* PULs contain GH16 genes distantly related to *zgal_236* (*cgkA*) and most likely forming a new GH16 subfamily. Within clades 1 and 2 we can define a core carrageenan utilization system which includes *dauA*, *dauB*, *dauC, dauD*, *dagA*, *cgsB*, *cgrA*, *cgtA*, and *cgtB*. Unexpectedly, the GH16 kappa-carrageenases and the GH82 iota-carrageenases are not part of the core system. Indeed some species harbor only one type of carrageenase, while others are deprived of any known carrageenases, suggesting that these latter bacteria may have new carrageenase families or that they can only degrade predigested oligosaccharides. Whereas the GH127 genes (*dagA*) are strictly conserved in the PUL, the GH129-like gene (*dagB*) is only found in a few species, suggesting that the ancestral α-1,3-(3,6-anhydro)-d-galactosidase activity was due to the GH127 family.

In clades 3, 4, and 5, the structure of the carrageenan-specific PUL is significantly modified. When considering the 30 bacterial species, the core system is restricted to the genes responsible for the release of D-AnG (*dagA*) and for its catabolism (*dauA*, *dauB*, *dauD*). This may be due to horizontal gene transfer (HGT) events from *Bacteroidetes* to other phyla. Somewhat surprising is the lack of *dauC* in some species. Such bacteria may use a non-phosphorylative variant of the Entner-Doudoroff pathway to degrade 2-keto-3-deoxy-d-galactonate, as observed in the archaeon *Picrophilus torridus* ^37^. The *susD*-like genes are unique to the *Bacteroidetes* ^8^ and transfer of the PUL to species belonging to other phyla has resulted in the loss of the *susCD*-like pair. This phenomenon is observed in the clade 3 Gammaproteobacteria (Fig. 9) and was previously identified in the case of alginolytic operons^19^. However, this does not mean that TBDT are absent in the gene clusters of other phyla. For instance, all clade 3 Gammaprotobacteria species have a TBDT gene in their cluster (CATDS2_v1220055 in *Catenovulum agarivorans* DS-2; JRKG01_v1_110122 in *Pseudoalteromonas* sp. PLSV; Patl_0887 in *P. atlantica* T6c; H978DRAFT_1909 in *Alteromonas* sp. ALT199). They are only distantly related to ZGAL_3581, but their location within the cluster strongly suggests that they play a similar role in the import of the carrageenan degradation product. Such clusters containing polysaccharide-related TBDT genes have already been described in Gammaproteobacteria^38^. The presence of carrageenan-specific PULs in gut bacteria from marine herbivorous animals is also reminiscent of the horizontal acquisition of porphyran/agar-related genes in animal and human intestinal symbionts^39^. This is quite clear for the surgeon fish symbiont *Epulopiscium* sp. (Fig. 9, clade 3), which is also known to have horizontally acquired GH16 porphyranase and GH117 genes from marine flavobacteria^40^. Phylogenetic analysis of the GH127 family unraveled another likely HGT case (Supplementary Fig. 13, Supplementary Data 12). Whereas ZGAL_3148 (DagA2, GH127-2) and ZGAL_3150 (DagA3, GH127-3) cluster only with homologs from marine bacteria harboring the carrageenan-specific PUL, ZGAL_3147 (DagA1, GH127-1) is at the root of a clade including GH127 enzymes from human gut *Bacteroides* species. These *dagA*1-like genes are within PULs including GH genes at first sight unrelated to carrageenans (e.g. GH78, GH95, GH105; Supplementary Fig. 14). Thus, these *Bacteroides* GH127 genes have a marine origin, but have most likely evolved in specificity after their horizontal acquisition.

### Conclusion

Here we have shown that PUL-like structures (lacking the *susCD*-like pair but maintaining other carrageenan-related genes) are found in bacterial phyla other than *Bacteroidetes*. Furthermore, the carrageenan utilization system is not static and can be characterized by gene losses and gene acquisitions with a dedicated core 3,6-anhydro-d-galactose metabolism that is conserved within carrageenolytic bacteria. This core system is essential but not sufficient for carrageenan utilization. Missing functions (e.g., carrageenases, some specific sulfatases) may be assumed by non-orthologous genes in different bacterial species. Therefore, polysaccharide utilization pathways are not always conferred by a single locus, even in *Bacteroidetes*, and may consist of a complex regulon. Moreover, our work experimentally strengthens the recent proposition^41^ that the PUL definition should not be restricted to the presence of a *susCD*-like pair and that this PUL notion should be extended to other bacterial phyla.

## Methods

### Materials

All materials were obtained from Sigma-Aldrich unless stated otherwise. Bacterial strains and plasmids used for phenotyping and complementation studies are listed in Supplementary Table 4.

### Cloning of target genes

All the *Z. galactanivorans* genes were cloned and overexpressed as previously described^42^. Briefly, genes were PCR-amplified using the NEB Q5 High-Fidelity DNA Polymerase system (Supplementary Table 2). PCR reactions were done with 30 cycles (denaturation: 95 °C; annealing: 60 °C; elongation: 72 °C) using 0.5 units of enzyme in a total reaction of 50 μL using the primers shown in Supplementary Table 2. Amplicons were cleaned up using the QIAquick PCR Purification Kit (Qiagen) and digested with the appropriate restriction endonucleases. All ligations were done in the linearized T7 system vector pFO4 (a MCS-modified pET15b) except for ZGAL_3151 which was cloned into pET20b.

### Protein production and purification

In general, *Escherichia coli* BL21(DE3) cells were transformed with the plasmids containing the gene fragment of interest then grown in the autoinduction Zyp-5052 medium^43^ (200 µg mL^−1^ ampicillin, 20 °C, 72 h). ZGAL_3152 (SeMet) was similarly produced in PASM-5052 medium^43^. The sulfatases were produced in 1 L Luria-Bertani medium supplemented with 100 µg/mL ampicillin, until reaching an OD_600_ of about 0.7. Sulfatase gene expression was induced with 0.1 mM isopropyl β-D-1-thiogalactopyranoside (IPTG) (overnight, 20 °C). In all cases, cells were collected by centrifugation at 3063×*g* for 30 min. After chemical cell lysis^44^, the lysate was clarified at 13,865×*g* for 45 min at 4 °C. Using an ÄKTA FPLC, the supernatant was loaded onto a 5 mL GE His Trap HP column, washed with 20 mM Tris pH 8.0 and 100 mM NaCl and eluted with an increasing gradient of 1–100% 20 mM Tris pH 8.0, 100 mM NaCl, and 1 M imidazole. Fractions containing the protein of interest were pooled and concentrated (MWCO 5 kDa) and then loaded onto a Superdex S200 column in 20 mM Tris pH 8.0 and 100 mM NaCl. Fractions were again pooled and concentrated. The ExPASy ProtParam tool^45^ was used to generate an extinction coefficient for calculation of protein concentration using the A280.

### Marine polysaccharide and oligosaccharide substrates

Carrageenan polysaccharides were obtained commercially from CP-Kelco. The kappa/mu- and kappa-carrageenans were extracted from *Kappaphycus alvarezii*, the iota/nu- and iota-carrageenans from *Euchema denticulatum* and the lambda-carrageenan from tetrasporophytes of *Gigartina skottsbergii*. Furcellaran, a beta-carrageenan from *F. lumbricalis* consisting primarily of beta- and kappa-carrabiose motifs, was obtained as a generous gift from CP Kelco (Brian Rudolph). The exception is alpha-carrageenan which was produced using the native sulfatase from *Pseudoalteromonas atlantica* which is active on IC using a previously developed protocol^6^. The oligo-iota-carrageenans were incubated with pure sulfatase ZGAL_3145 to produce oligo-iota/alpha-carrageenans. One volume of oligo-iota-carrageenans (0.5% w/v in H_2_0 mQ) was incubated at 37 °C over 48 h with the same volume of the sulfatase at 1.0 mg mL^−1^ in 50 mM Tris pH 8.0, 200 mM NaCl, 1 mM CaCl_2_. Oligosaccharides of furcellaran, kappa- and iota-carrageenans were produced using the recombinant kappa-carrageenase from *Pseudoalteromonas carrageenovora* ^46^ and iota-carrageenase from *Alteromonas fortis* ^47^. Carrageenans (0.25%, 5 mL) were treated 48 h at 37 °C with kappa-carrageenase or iota-carrageenase (0.3 mg mL^−1^, 50 μL) in 100 mM HEPES pH 7.5 and 25 mM NaCl. After checking the total hydrolysis, oligosaccharides mixtures were fractionated by size-exclusion chromatography (SEC). To this end, 5 mL of hydrolysate (concentrated by rotary evaporation at about 5% w/v) were filtered (0.2 µm, Millipore), and injected on 3 GE Healthcare Superdex 30 prep-grade columns (600 × 26 mm i.d.) mounted in series. The elution was conducted at a flow rate of 1 mL/min at 20 °C using 50 mM (NH_4_)_2_CO_3_ as the eluent. Oligosaccharides were detected by differential refractometry (Spectra System RI-50, Thermo Separation products) and fractions of 5 mL were collected.

### Sulfatase activity assays

*HPLC*. Carrageenan-sulfatase activity was measured by high-performance anion-exchange chromatography (HPAEC), according to a protocol adapted from Préchoux et al.^7^. Carrageenan solutions (0.5 % w/v in H_2_O mQ) were incubated in presence of purified sulfatases (0.5 mg mL^−1^) over 20 h at 37 °C in 25 mM Tris pH 8.0, 0.1 M NaCl, 0.5 mM CaCl_2_. For each reaction, a control sample was realized in similar conditions but with inactivated enzyme (100 °C, 10 min). Reactions were filtered (10 kDa, Amicon Ultra, *Millipore*) then injected (AS-AP Autosampler) onto an AG11-HC guard column (4 × 50 mm) mounted in series with an AS11-HC anion-exchange column (4 × 250 mm) using an ICS5000 system (*Thermo Scientific Dionex*). Elutions for the detection of sulfate were performed with isocratic 12 mM NaOH at a flow rate of 1 mL min^−1^ (Single Pump-5), and the detection of anions was done by an Analytical CD Conductivity Detector associated to a suppressor (ASRS 500, 4 mm) running at 50 mA. Using a standard curve of sulfate and through integration of the peaks, the concentration of sulfate released by the enzymatic reaction was calculated from the difference in the amount of free sulfate (retention time at 4 min) between samples and their associated blanks. For oligosaccharide detection HPAEC analyzes were conducted on the same system described for sulfate quantification. Elutions were performed at a flow rate of 0.5 mL min^−1^ using a NaOH multistep gradient from 8 to 280 mM (40 min). Oligosaccharides were detected by conductivity mode under a current suppression of 50–300 mA.

^*1*^ *H NMR*. 800 µL of carrageenan (1% w/v in H_2_O mQ) was incubated at 37 °C with the purified sulfatase (0.5 mg/ml), over several days. After checking for sufficient desulfation by HPAEC, incubation media were freeze-dried, exchanged twice then dissolved in D_2_O (99.9%) to 10 mg mL^−1^. Samples were transferred into 5-mm NMR tubes and ^1^H-NMR spectra were recorded at 70 °C, on 500 MHz Bruker AVANCE III HD spectrometer, equipped with an indirect 5-mm gradient probehead TBI ^1^H/(BB)/^13^C. Chemical shifts were referenced with respect to trimethylsilylpropionic acid (TSP) used as an external standard, and expressed in ppm. For these experiments, the spectra required 64 scans.

### Glycoside hydrolase activity assays

Small scale GH127 and GH129-like (ZGAL_3152) reactions were set up in two formats. Volume of 100 mL of Zyp5052 autoinduction medium was inoculated with *E. coli* BL21(DE3) transformed with the pFO4 vector alone as a control or with the GH clones and grown 72 h at 20 °C. Cells were collected from 2 mL of the cultures, resuspended in 2 mL 50 mM HEPES pH 7.5 and 150 mM NaCl and lysed using a French pressure cell. The lysate was clarified at 13,865×*g* for 45 min and the supernatant was used for the enzyme activity tests which were set up as follows in a 20 μL reaction volume: 0.5% furcellaran hexasaccharide, 150 mM NaCl, 3.75 mM CaCl_2_, 100 mM HEPES pH 7.5 and 2 μL soluble cell lysate. Alternatively, instead of soluble cell lysate, 1 μL of purified enzyme (1 mg mL^−1^) was added to the same reaction mixture described above. Reactions were left overnight at room temperature. For the GH2 enzymes, the sequential digest reactions were set up with 150 μL 0.25% furcellaran oligosaccharides that had been predigested with the purified GH129-like enzyme, as described above, and then heat denatured for 10 mins in a water bath at 90 °C. The 200 μL reactions were incubated overnight at 37 °C in the following conditions: 5 μg purified GH2, 1 mM CaCl_2_, 1 mM MgCl_2_ and 20 mM HEPES pH 7.5.

### Fluorophore assisted carbohydrate electrophoresis

A speed vacuum was used to dry ~50 μg of oligo- or polysaccharide reaction volume to be destined for FACE^31^. Volume of 2 μL of 0.15 M ANTS (8-aminonaphthalene-1,3,6-trisulfonic acid) in a solution of acetic acid and water (3:17) was added to the dried reaction followed by 5 µL of freshly made 1 M sodium cyanoborohydride in DMSO. The samples were incubated overnight at 37 °C in the dark then resuspended in 20 µL of 20% glycerol. Between 2 and 10 µL of sample was loaded onto a 27% polyacrylamide gel and migrated at 200 V for 2 h in the dark at 4 °C. The gels were visualized under UV light.

### 3,6-Anhydro-d-galactose dehydrogenase activity assay

The enzymatic activity of ZGAL_3155 (DauA, 3,6-Anhydro-d-galactose dehydrogenase) was determined spectrophotometrically as a function of the reduction of NAD^+^ or NADP^+^ using a Spark 10 M microplate reader (Tecan, France). The reaction was performed in 25 mM Tris-HCl (pH 7.5) containing 100 mM NaCl and the reaction mixture (250 µL) contained 0.8–4.0 µg of pure recombinant dehydrogenase, 10 mM D-AnG (Dextra) and 1.5 mM NAD^+^ or NADP^+^. A negative control was performed by adding 4 µg pure ZGAL_3155 denatured for 10 min at 100 °C, all other conditions were the same within the reaction. An additional control using 10 mM d-galactose instead of D-AnG was also included. The absorbance at 340 nm was followed as a function of time until the absorbance reached a plateau. The activity of the enzyme is expressed as the number of µmoles of NADH produced min^−1^ mg^−1^ assuming the ε_340 nm_ NADH = 6220 M^−1^ cm^−1^. Each reaction was performed in triplicate. At the end of the reaction, the reaction mixture was denatured for 10 min at 100 °C. The reaction mixture was then centrifuged for 10 min at 29,000×*g* and the supernatant was used as the substrate for the next enzyme, ZGAL_3156.

### 3,6-Anhydro-d-galactonate cycloisomerase activity assay

The enzymatic activity of ZGAL_3156 (DauB, 3,6-Anhydro-d-galactonate cycloisomerase) was determined using the 2-thiobarbituric assay (TBA)^33,48^. Volume of 250 µL of the reaction mixture from the preceding enzymatic step (the conversion of D-AnG into 3,6-anhydro-d-galactonate through the action of ZGAL_3155) was incubated with 10–40 µg of pure recombinant ZGAL_3156. The reaction (final volume 300 µL) was performed at room temperature in 25 mM MES pH 6.5 containing 100 mM NaCl (pH 7.5) over 20 min. To measure the activity, aliquots (25 µL) were withdrawn at the 20 min time point and the reaction was stopped with 1/10 volume of 12% trichloroacetic acid (TCA). Samples were then centrifuged for 10 min at 29,000×*g*. Volume of 25 µL of the supernatant was then incubated for 20 min at room temperature in the dark with 62.5 µL 25 mM periodic acid in 250 mM H_2_SO_4_ to oxidize 2-keto-3-deoxy-d-galactonate. Oxidation was terminated by the addition of 125 µL 2% (w/v) sodium arsenite in 500 mM HCl. 500 µL 0.3 % (w/v) TBA was then added and the reaction mixture was incubated for 10 min in a boiling water bath. After cooling down to room temperature, a sample of the solution was removed. The color was intensified by the addition of an equal volume of dimethylsulfoxide and the absorbance was measured at 549 nm. To produce the substrate for the next reaction, the incubation with ZGAL_3156 was performed for 1 h with 50 µg of enzyme at room temperature. The enzyme was then inactivated for 10 min at 100 °C and centrifuged for 10 min at 29,000×*g* and 150 μL of supernatant was used as the substrate for ZGAL_3154.

### 2-keto-3-deoxy-d-galactonate kinase activity assay

The enzymatic activity of ZGAL_3154 (DauC, 2-keto-3-deoxy-d-galactonate kinase) was determined indirectly as a function of the oxidation of NADH using an NADH coupled assay. Reactions were performed in 25 mM MES buffer (pH 6.5). A typical reaction mixture (total volume 193 µL) contained 150 µL of reaction mixture from the preceding step, 2 µg pure recombinant ZGAL_3154, 0.97 mM adenosine-5-triphosphate (ATP), 9.7 mM MgCl_2_ (Acros organics), 0.8 mM Phospho(enol)pyruvic acid tri(cyclohexylammonium) salt, 0.16 mM β-NADH (Applichem), 8 mM KCl, and 0.95 µL of mix of pyruvate kinase and lactic dehydrogenase enzymes from rabbit muscle (1.1 Units of pyruvate kinase and 0.8 Units of lactate dehydrogenase, respectively). The reactions were performed at room temperature in 96-wells UV-Star plates (Greiner) and the decrease of absorbance at 340 nm was read every 5 s with a Spark 10 M microplate reader (Tecan, France). The absorbance at 340 nm was followed as a function of time until it stabilized. A blank was performed using 2 µg pure ZGAL_3154 denatured for 10 min at 100 °C with all other reaction components the same.

### 2-keto-3-deoxy-d-galactonate aldolase activity assay

ZGAL_3153 (DauD, 2-keto-3-deoxy-d-galactonate aldolase) activity was verified using the TBA assay (as described above) to measure cleavage or synthesis of 2-keto-3-deoxy-6-phospho-d-galactonate (D-KDPGA). Degradation was measured using 15 µl of product of ZGAL_3154 incubated 10 min with 5 µg of ZGAL_3153. Second, synthesis of D-KDPGA from pyruvate and d-Glyceraldehyde-3-phosphate or d-glyceraldehyde was tested as follows: 30 µl of MES 25 mM pH 6.5, pyruvate 50 mM and d-glyceraldehyde-3-phosphate or d-glyceraldehyde 20 mM were incubated with 5 µg of ZGAL_3153 for 10 min. Both reactions were done in triplicate at room temperature and stopped by addition of 10% (v/v) of TCA 12%. The negative control was done with the same quantity of enzyme, previously boiled for 10 min.

### Affinity gel electrophoresis

Interaction of the SusD-like proteins ZGAL_3580 and ZGAL_3638 with polysaccharides was tested by affinity gel electrophoresis. Kappa-carrageenan (from *Kappaphycus alvareizii*), iota-carrageenan (*Eucheuma denticulum*), lambda-carrageenan (Dupont batch 2321914837), agar (Sigma), porphyran (water extraction from *Porphyra* sp.) and furcellaran (CP Kelco) were incorporated at a final concentration of 0.21% in native 10% acrylamide gels. ZGAL_3580 (4 µg), ZGAL_3638 (8 µg) and bovine serum albumin (BSA, 6 µg) were loaded into the wells and migrated at 60 V over 5 h. Gels were stained with Coomassie Blue solution.

### Protein crystallization

Initial hits were obtained using hanging drop vapor diffusion in the JCSG + screen (Qiagen) for ZGAL_3156 and the PACT screen (Qiagen) for ZGAL_3152. Optimized conditions were a 1:1 ratio of 0.14 M Na/K-tartrate, 12% PEG 3350 with 9 mg mL^−1^ ZGAL_3152 and 0.2 M tri potassium citrate, 20% PEG 3350 with 25 mg mL^−1^ ZGAL_3156.

### X-ray crystallography data collection and processing

SeMet ZGAL_3152 diffraction data were collected at the ESRF on beamline ID-14-4 at the selenium peak with a wavelength of 0.97936 Å (*f*′ = −7.40, *f*″ = 6.38) at 100 K. The cryoprotectant used was 15% MPD in mother liquor. The data were processed using MOSFLM^49,50^, Pointless^51^ was used to determine the spacegroup and the data were scaled using SCALA^52^ within the CCP4 suite of programs^53^. PrepHAData was used to convert the mtz to SHELXS format and SHELX_CDE^54^ was used to identify the Se subsites through SAD. Finally, CRANK^55^ was used to construct the initial model of ZGAL_3152 through substructure refinement and model building. This model was then used to solve the structure of the native ZGAL_3152 by molecular replacement using MOLREP^56^ (pdb id 5opq, Supplementary Table 1). The ZGAL_3152 structure went through an iterative process of refinement using REFMAC5^57^ and model building using WinCoot^58^. Ramachandran statistics were 96.6% most favored, 3.2% additional allowed and 0.2% disallowed.

Native diffraction data for ZGAL_3156 was collected on the Proxima2 beamline at SOLEIL using an EIGER detector (Dectris), with a wavelength of 0.98 Å and an oscillation range of 0.1° at 100 K. The crystals were cryoprotected by short immersion in mother liquor supplemented with 15% Glycerol. The data were processed and scaled using XDS and XSCALE^59,60^, Pointless was used to determine the spacegroup^51^. The structure (pdb id 5olc, Supplementary Table 1) was solved by molecular replacement using PHASER^61^ and pdb id 4hpn using the biological assembly (octamer) as the primary model. The ZGAL_3156 structure went through an iterative process of refinement using BUSTER^62^ and model building using WinCoot^58^. Ramachandran statistics were 94.4% most favored, 5.3% additional allowed and 0.3% disallowed.

### MALDI-TOF-MS analysis

MALDI-TOF-MS spectra were acquired in negative ionization mode and reflector detection in the m/z range 500–1500 on an AutoflexSpeed TOF/TOF mass spectrometer (Bruker Daltonics, Bremen, Germany), equipped with a Smartbeam Laser (355 nm, 1000 Hz). Samples were solubilized in water (500 µg mL^−1^). Volume of 1 µL of the samples were mixed with 1 µL of a DMA-DHB matrix solution prepared as described^63^ directly on a polished steel MALDI target plate. Acquisition parameters (laser power, pulsed ion extraction, and so on) were optimized on the sample without any enzyme process. Spectra were recorded using FlexControl 3.4 and processed using FlexAnalysis 3.4 (Bruker Daltonics, Bremen, Germany).

### Construction of dagA3, dagB, dauA, and cgrA deletion mutants

Single deletion mutants for *dagA*3 (ZGAl_3150, GH127-3), *dagB* (ZGAL_3152, GH129-like), *dauA* (ZGAL_3155, 3,6-Anhydro-d-galactose dehydrogenase), and *cgrA* (ZGAL_3159, AraC family regulator), and a double deletion mutant for *dagA*3/*dagB* were constructed following the previously described method^35^, based on a *sacB* system (Supplementary Tables 3, 4). To delete *dagA*3, a 2.1-kbp fragment including the first 33 bp of *dagA*3 and 2,058 bp of upstream sequence was amplified using primers OFT0001 and OFT0003. The fragment was digested with PstI and SalI and ligated into pYT313 that had been digested with the same enzymes, to generate pFT2. A 2.1 kb fragment including the final 33 bp of *dagA*3 and 2,013 bp of downstream sequence was amplified using primers OFT0002 and OFT0004. The fragment was cloned into SalI and BamHI sites of pFT2 to generate the *dagA*3 deletion construct pFT5. To delete *dagB*, a 2.1-kbp fragment including the first 48 bp of ZGAL_3152 and 2047 bp of upstream sequence was amplified using primers ORL664 and ORL665. The fragment was digested with BamHI and SalI and ligated into pYT313 that had been digested with the same enzymes, to generate pRF8. A 2.2 kb fragment including the final 3 bp of *dagB* and 2172 bp of downstream sequence was amplified using primers ORL666 and ORL667. The fragment was cloned into SalI and SphI sites of pRF8 to generate the *dagB* deletion construct pRF9. To delete *dauA*, a 2.0-kbp fragment including the first 81 bp of *dauA* and 1887 bp of upstream sequence was amplified using primers ORL670 and ORL671. The fragment was digested with BamHI and SalI and ligated into pYT313 that had been digested with the same enzymes, to generate pRF10. A 2.2 kb fragment including the final 69 bp of *dauA* and 2116 bp of downstream sequence was amplified using primers ORL672 and ORL673. The fragment was cloned into SalI and PstI sites of pRF10 to generate the *dauA* deletion construct pRF11. To delete *cgrA*, a 2.1-kbp fragment including the first 5 bp of *cgrA* and 2030 bp of upstream sequence was amplified using primers ORL676 and ORL677. The fragment was digested with BamHI and SalI and ligated into pYT313 that had been digested with the same enzymes, to generate pRF12. A 2.2 kb fragment including the final 73 bp of *cgrA* and 2064 bp of downstream sequence was amplified using primers ORL678 and ORL679. The fragment was cloned into SalI and PstI sites of pRF12 to generate the *cgrA* deletion construct pRF13. Plasmids pFT5, pRF9, pRF11 and pRF13 were introduced individually into the wild-type *Z. galactanivorans* Dsij^T^ by conjugation from *E. coli* S17_1 strains. Conjugants with plasmids integrated in the genome were isolated on *Cytophaga*-agar containing 50 µg mL^–1^ erythromycin. Single erythromycin-resistant colonies were grown overnight in *Cytophaga* medium in the absence of antibiotics at 30 °C. The cells where a second recombination event resulted in loss of the plasmid were selected on *Cytophaga*-agar containing 5% sucrose. Isolated colonies were checked for erythromycin sensitivity. Deletions were confirmed by PCR on isolated colonies using primer pairs OFT0005-OFT0006 to identify the *dagA*3 deletion mutant (mZG_0026), ORL668-ORL669 to identify the *dagB* deletion mutant (mZG_0007), ORL674-ORL675 to identify the *dauA* deletion mutant (mZG_0008) and ORL680-ORL681 to identify the *cgrA* deletion mutant (mZG_0011). A double deletion mutant for both *dagA*3 and *dagB* (mZG_0047) was constructed by introducing pRF9 into Δ*dagA*3 by conjugation, followed by sucrose selection and PCR confirmation as described above.

### Construction of cgtA-cgtB double gene deletion mutant

To delete *cgtA-cgtB* [*zgal_3581* (*susC-like*)-*zgal_3580* (*susD-like*)], a 2.1-kbp fragment including the first 54 bp of *cgtA* and 2060 bp of upstream sequence was amplified using primers 2088 and 2089 (Supplementary Tables 3, 4). The fragment was digested with SacI and SpeI and ligated into pYT354 that had been digested with the same enzymes, to generate pYT381. A 2.2 kb fragment including the final 54 bp of *cgtB* and 2,169 bp of downstream sequence was amplified using primers 2090 and 2091. The fragment was cloned into SpeI and SphI sites of pYT381 to generate the *cgtA-cgtB* deletion construct pYT382. Primers 2102 and 2103 were used to identify the *cgtA-cgtB* deletion mutant (mZG_0054).

### Complementation of dauA and cgrA deletion mutants

Deletion mutants Δ*dauA* and Δ*cgrA* were complemented by ectopic plasmid integration at a neutral site, as previously described^35^ (Supplementary Tables 3 and 4). To complement Δ*dauA*, promoterless *dauA* was amplified with primers 1978 and 1979 and cloned into the XbaI and SphI sites of pYT356 to generate pYT360. Plasmid pYT360 was inserted into Δ*dauA* by conjugation to obtain the complemented strain Δ*dauA* + CP (complementation plasmid) (mZG_0039). To complement Δ*cgrA*, promoterless *cgrA* was amplified with primers 1980 and 1981 and cloned into the XbaI and SphI sites of pYT356 to generate pYT361. Plasmid pYT361 was inserted into Δ*cgrA* by conjugation to obtain the complemented strain Δ*cgrA* + CP (mZG_0043). Control strains with the empty vector pYT356 inserted into the chromosome were generated in a similar way. In all cases, cells with integration of the plasmid at the neutral site were selected on *Cytophaga*-agar containing 50 µg mL^−1^ erythromycin and screened by PCR.

### Liquid growth tests

*Z. galactanivorans* strains were routinely grown from glycerol stocks in Zobell medium 2216E (5 g L^−1^ tryptone, 1 g L^−1^ yeast extract, filtered seawater)^64^. Before use, all pre-cultures were collected by centrifugation 10 min at 2844×*g*, washed in 2 volumes of sterile saline solution, and resuspended in sterile saline solution to the same OD_600_. Fifty microliters of bacterial suspension were inoculated into triplicate 40 mL flasks containing 5 mL of Zobell medium or marine mineral medium (MMM) composed for 1 L of 24.7 g NaCl, 6.3 g MgSO_4_·7H_2_O, 4.6 g MgCl_2_·H_2_O, 2 g NH_4_Cl, 0.7 g KCl, 0.6 g CaCl_2_, 200 mg NaHCO_3_, 100 mg K_2_HPO_4_, 50 mg yeast extract, and 20 mg FeSO_4_·7H_2_O^19^ and supplemented with 4 g L^−1^ kappa-carrageenan, iota-carrageenan, agar, d-galactose or D-AnG. Growth at 20 °C under 160 r.p.m. was followed by measuring the absorbance at 600 nm in a Spark 10 microplate reader (Tecan) on 200 µL aliquots. Erythromycin (10 µg mL^−1^) was added to all experiments with strains mZG_0031, mZG_0037, mZG_0039, mZG_0041, and mZG_0043 (Supplementary Table 4).

### Solid growth tests

Degradation of carrageenans was tested on either ZoBell or MMM medium solidified with 10 g L^−1^ kappa-carrageenan or 20 g L^−1^ iota-carrageenan. Two microliters of cell suspension prepared as described above were spotted in the center of the Petri dish, and incubated at 30 °C. Degradation was evidenced by the formation of a hole for kappa-carrageenan or liquefaction for iota-carrageenan.

### RNA-seq expression profiling

*Bacterial strain and culture conditions.* The type strain Dsij^T^ of *Z. galactanivorans* was routinely grown in Zobell medium 2216E (Difco) at 28 °C, 170 r.p.m. For transcriptome profiling, cells were cultivated in synthetic Marine Mineral Medium (MMM)^65^. MMM was supplemented with different carbon sources: d-galactose (Sigma-Aldrich #G0750), iota-carrageenan (Danisco, 2544-88-20), kappa-carrageenan (Coffoni ×6913) or D-AnG (Dextra Laboratories #G0002). Briefly, overnight cultures performed in Zobell medium were diluted at OD_600_ 0.05 in triplicate MMM containing 0.5% glucose as C source and incubated at 28 °C until reaching the stationary phase. These cultures were then used to inoculate 10 mL of MMM containing 0.4 g L^−1^ d-galactose, D-AnG, kappa- or iota-carrageenan. When cell density reached an OD_600_ of 0.7 (±0.1) bacteria were collected by centrifugation for 3 min at 4 °C after addition of ½ volume of frozen killing buffer (20 mM Tris-HCl pH 7.5, 5 mM MgCl_2_, 20 mM NaN_3_) to the culture sample. The cell pellets were frozen in liquid nitrogen and stored at −80 °C until RNA extraction.

*RNA extraction*. The cell pellets were resuspended into 800 μL of lysis buffer (4 M guanidine thiocyanate, 25 mM sodium acetate pH 5.2, 5 g L^−1^ N-laurylsarcosinate), immediately mixed to 1 mL hot acid phenol (Sigma #P4682) and incubated during 5 min at 65 °C for efficient cell lysis. The aqueous phases were recovered after addition of 1 mL chloroform and centrifugation at 16,000×*g* during 10 min at room temperature. The samples were extracted at least four times with an equal volume of acid phenol:chloroform:IAA (25:24:1, (pH 4.5)) and once with chloroform. Total RNAs were ethanol precipitated at room temperature and the pellets resuspended in RNase-free water. The RNA concentration of samples was measured using a NanoDrop 1000 Spectrophotometer (NanoDrop Technologies, Inc.). The quality of RNA preparations was assessed by capillary electrophoresis using RNA Nano chips with a Bioanalyzer Agilent 2100 (Agilent Technologies, Palo Alto, USA). Total RNA extracts were treated using DNase I (Qiagen) to remove residual genomic DNA.

*mRNA enrichment, RNA-seq library preparation and sequencing*. mRNA enrichment was performed using the Illumina Ribo-Zero rRNA Removal Kit (Bacteria). The rRNA-depleted samples were purified using a RNeasy MinElute Cleanup Kit (Qiagen) and efficient rRNA depletion was confirmed by Bioanalyzer 2100. Strand-specific RNA-seq libraries were generated with the ScriptSeq V2 RNA-Seq Library Preparation Kit (Illumina), according to the manufacturer’s instructions. Briefly, 2–4 µg of RNA samples were fragmented, di-tagged cDNAs were synthetized by successive random priming with terminal-tagging oligos and then purified with the Ampure bead XP system. Enrichment of purified cDNAs was done by no more than 14 PCR cycles. The sequencing was performed with a NextSeq 500 platform using a single-end 75 bases run. A total of 3.5–11 million passing filters reads were obtained per sample.

*Read mapping and differential expression analysis*. Sequencing reads were pre-processed for trimming of adapter sequences with cutadapt-1.9.1, then using PRINSEQ^66^ for read quality with filtering (-min_len 20 -min_qual_mean 20 -min_qual_score 10) and trimming (trim_qual_right 10) options. Mapping was performed on *Z. galactanivorans* Dsij^T^ reference genome (retrieved from MicroScope “zobellia_gal_DsiJT_v2”; Refseq NC_015844.1) by Bowtie2 software with the very-sensitive option^67^. Alignments files were then converted to BAM files using SAMtools^68^, log2-genome coverage files were computed as described^69^ and these expression profiles can be visualized in the Artemis viewer^70^. The number of reads mapping to each predicted CDS was calculated for the 12 data sets by HTSeq-count^71^ with the -m union option. Differential expression analysis (normalization and statistical tests) was performed using the SARTools with DESeq2 software^72^ and a bonferroni *p*-value adjustment was used to correct for multiple testing. Genes with an adjusted *p*-value <0.05 were considered as differentially expressed. RNA-seq data are presented in Table 1, Supplementary Data 2.

### PUL identification and comparison

Similar PULs were detected in 29 organisms using synteny results of the MicroScope platform^73^ (Supplementary Data 10–11, Supplementary Fig. 12). The proteins from identified PULs and from *Z. galactanivorans* regulon were then manually grouped in homolog clusters based on sequence similarity. From these 40 clusters, presence/absence of homolog proteins encoded outside the PULs was determined by blastP alignments with at least 35% of amino acid identity and 80% coverage threshold. Results were then gathered in a matrix to indicate, for each organism, if a protein cluster homolog is encoded in the PUL (value 2), elsewhere on the genome (value 1) or absent (value 0). From this matrix, a heat map and a hierarchical classification of the organisms were made using heatmap.2 function and the ward.D2 algorithm with Manhattan distances from gplots and hclust R packages, respectively (Fig. 9).

### GH127 phylogeny reconstruction

549 proteins having a GH127 catalytic module, which aligned on more than 60% of the DBCAN domain, were extracted from the MicroScope platform. Protein sequences were aligned using MAFFT v7.307^74^, then ambiguous and saturated regions were removed with BMGE v1.12 (with the gap rate parameter set to 0.5)^75^. The best fitting model of amino acid substitution for this data set was selected with ProtTest v3.4.2^76^. A Maximum-Likelihood phylogenetic tree was generated with the alignment using PhyML 3.1.0.2^77^ using the LG amino acid substitution model with gamma-distributed rate variation (four categories), estimation of the proportion of invariable sites and exploring tree topologies. 100 bootstrap replicates were performed. The phylogenetic tree was displayed and annotated using the interactive tree of life (iTOL) online tool^78^ (Supplementary Figs. 13, 14, Supplementary Data 12).

### Data availability

The coordinates and structure factors for the proteins described above have been deposited in the Protein Data Bank (pdb id: 5opq and 5olc). The RNA-seq transcriptomic data have been deposited in the GEO database (GEO accession number: GSE101142). The sequence of the *Tenacibaculum jejuense* genome (used in the comparative genomic analysis) has been deposited at EMBL (accession: GCA_900198195). All other relevant data are available in this article and its Supplementary Information files, or from the corresponding author upon request.

## Electronic supplementary material

Supplementary Information

Peer Review File

Description of Additional Supplementary Files

Supplementary Data 1

Supplementary Data 2

Supplementary Data 3

Supplementary Data 4

Supplementary Data 5

Supplementary Data 6

Supplementary Data 7

Supplementary Data 8

Supplementary Data 9

Supplementary Data 10

Supplementary Data 11

Supplementary Data 12
