# Supplementary material for: Carrageenan catabolism is encoded by a complex regulon in marine heterotrophic bacteria
Source: Nat Commun. 2017 Nov 22;8:1685. doi: 10.1038/s41467-017-01832-6 (PMC5698469; doi:10.1038/s41467-017-01832-6)
Supplement: Supplementary file 1 — Supplementary Information [file 41467_2017_1832_MOESM1_ESM.pdf]

Supplementary Discussion

Initial Glycoside Hydrolase Activity Assays.

The PUL ZGAL_3145-3159 encodes three GH127s and one GH129-like enzyme (ZGAL_3147, ZGAL_3148, ZGAL_3150 and ZGAL_3152, respectively). The closest characterized homologues are found in enteric bacteria: BT1003 (*Bt*GH127) from *Bacteroides thetaiotaomicron* is a 3-C-carboxy-5-deoxy-L-xylose (aceric acid)-hydrolase involved in depolymerization of rhamnogalacturonan-II[1]; *Bl*GH127 from *Bifidobacterium longum* is an exo-β-L-arabinofuranosidase acting on the plant glycoproteins extensins[2]; *Bb*GH129 from *B. bifidum* hydrolyses alpha-linked N-acetyl-D-galactosamine from Ser/Thr (the Tn antigen), a component of intestinal mucin[3]. Within our group we have recently identified arabinogalactan-like proteins (AGPs) in brown algae[4]. They are important in development in plants and have been very well studied; however, this was the first time such proteins have been described in algae. In plants AGPs consist of primarily beta-1,3-galactan main chains with beta-1,6-galactan side chains often with arabinose at the termini. The protein backbone contains repeating motifs predominantly consisting of hydroxyproline, proline, alanine, threonine and serine residues with the hydroxyproline residues as the principal sites for O-glycosylation. Based on these observations we initially hypothesized that the PUL was specific for AGPs in brown algae with ZGAL_3152 enzyme specific for cleavage of alpha-linked D-galactose from hydroxyproline. In order to test this hypothesis we purchased the Tn-Antigen (D-galactose-alpha-1-L-serine) and synthesized the following compounds: D-galactose-1-(alpha/beta)-L-hydroxyproline, D-galactose-1-(alpha/beta)-L-serine and D-galactose-1-(alpha/beta)-L-threonine[5]. ZGAL_3152 did not demonstrate activity on any of these compounds, leading us to refine our overall hypothesis for the PUL.

Determination of Sulfatase Specificities.

Sulfatase ZGAL_3146 (CgsB1)

The ^{1}H NMR spectrum of kappa-carrageenan (Supplementary Fig. 2CI) presents the characteristic signal of the anomeric proton (DA-H1) at 5.12 ppm (Nomenclature of Knutsen and coworkers, Supplementary Fig. 2A)[6]. After incubation with the sulfatase ZGAL_3146 a new signal very close to the kappa signal appears at 5.10 ppm (Supplementary Fig. 2CII). This assignment is attributed to the anomeric proton of beta-carrabiose residue[7] confirming the desulfation of kappa-carrageenan and the activity of ZGAL_3146 as a kappa-carrageenan G4S-sulfatase.

Sulfatase ZGAL_3145 (CgsA)

The sulfatase ZGAL_3145 demonstrated activity on iota-carrageenan, both on polymer and on oligosaccharides (Supplementary Fig. 2B, 3A and 4A and 4BII). The ^{1}H NMR analyses shows the conversion of iota- into alpha-carrageenan. On the 1H NMR spectra of iota-carrageenan (Supplementary Fig. 3AI) we can observe the characteristic assignment of the signal corresponding to the anomeric proton (DA2S-H1) at 5.32 ppm. After incubation of this polymer with ZGAL_3145, we observe the emergence of a new signal at 5.26 ppm (Supplementary Fig. 3AII), due to the production of alpha-carrabiose residues[8]. Thus, ZGAL_3145 removes the sulfate group on position 4 of the β-linked galactose (G unit) of iota-carrabiose moieties (G4S-DA2S). The desulfation of iota/nu-carrageenan (Supplementary Fig. 3B) by ZGAL_3145 reinforces this result. Indeed, we can see the diminution of the G4S-H4 proton of iota-carrabiose at 4.92 ppm, and unambiguously the appearance of G-H1 and G-H4 at 4.62 and 4.13 ppm (Supplementary Fig. 3BII), due to the removal of the sulfate group. Furthermore, nu-carrageenan has a characteristic signal for its alpha-anomeric proton, D2S,6S-

H1 at 5.5 ppm[9]; the nu-carrabiose residues are not affected by treatment with ZGAL_3145. Therefore, ZGAL_3145 is active as an iota-carrageenan G4S-sulfatase.

Sulfatase ZGAL_3151 (CgsC)

ZGAL_3151 did not demonstrate activity on alpha-carrageenan polymer (Supplementary Fig. 2B); however, using HPLC, the enzyme showed activity on alpha/iota-carratetraoses, which were produced by the incubation of iota-carratetraoses with CgsA (ZGAL_3145) (Supplementary Fig. 2B, 4A and 4BIII). Briefly, the ^{1}H NMR spectra of the untreated iota-carratetraose shows the characteristic anomeric signals of around 5.30-5.35 ppm (Supplementary Fig. 4BI)[10]. The signals of the non-reducing DA2S unit and of the reducing disaccharide unit G4S-DA2S are found on the right and left of the 5.30-5.35 ppm region, respectively. After incubation with CgsA, we observe a slight shift of the signal of the non-reducing DA2S proton H1(Supplementary Fig. 4BII), corresponding to the removal of the sulfate group in position 4 of the non-reducing unit indicating that the product of the CgsA reaction with iota-carratetraose is an alpha/iota-carratetraose with the alpha-disaccharide unit on the non-reducing side. After incubation of the alpha/iota-carratetraose with ZGAL_3151, the signals from the iota-carrabiose on the reducing side are conserved (at 5.35, 5.34 and 5.32 ppm), but the signal from the D-AnG on the non-reducing side (5.26 ppm) are strongly shifted (5.09 ppm) due to its desulfation in position 2 (Supplementary Fig. 4BIII). This NMR profile reveals the conversion of the alpha/iota-carratetraose into a hybrid of iota/beta-carratetraose with the beta disaccharide unit on the non-reducing side. Thus, ZGAL_3151 is active as an alpha-carrageenan DA2S-sulfatase, catalyzing the removal of the ester-sulfate group on the C2 of the DA2S moiety of the alpha-carrabiose motif.

Phenotyping of the AraC-family regulator CgrA (ZGAL_3159)

Wild type *Z. galactanivorans* grew well on Marine Mineral Medium (MMM) and Zobell medium gelified by kappa-carrageenan and in both cases created a depression due to kappa-carrageenan degradation (Supplementary Fig. 10E,F). Thus, kappa-carrageenase production is not repressed by the presence of tryptone and yeast extract as additional carbon sources. In contrast, Δ*cgrA* was unable to grow on kappa-carrageenan alone (Supplementary Fig. 10G), but did grow without creating a depression on kappa-carrageenan in Zobell media (Supplementary Fig. 10H). Thus, kappa-carrageenase expression is under control of CgrA. Wild type *Z. galactanivorans* grew well on MMM and Zobell medium gelified by iota-carrageenan. In both cases the iota-carrageenan gel was liquefied by the organism indicating iota-carrageenase production is not repressed by the presence of tryptone and yeast extract as additional carbon sources (Supplementary Fig. 10I,J). The mutant Δ*cgrA* showed severely impaired growth compared to the wild type on iota-carrageenan alone (MMM) with a small amount of liquefaction (Supplementary Fig. 10K); however, Δ*cgrA* grew on and liquefied the Zobell medium gelified by iota-carrageenan (Supplementary Fig. 10L). Thus, at least one of the three iota-carrageenases is not under strict control of CgrA. Examination of the RNAseq data shows an up-regulation of the iota-carrageenases CgiA1 and CgiA3 and a strong down-regulation of CgiA2 in iota-carrageenan. Therefore, the differential regulation of the iota-carrageenases is confirmed and *cgiA2* is not under strict control of CgrA.

GH2 beta-1,4-galactosidase activity on carrageenan oligosaccharides

The carrageenan PUL does not encode an enzyme dedicated to the hydrolysis of the beta-1,4-linkage in carrageenan. We hypothesized that *Z. galactanivorans* produces a GH2 beta-galactosidase enzyme for the hydrolysis of the non-reducing exo-D-galactose beta-1,4-linkage. One GH2 in particular,

ZGAL_3633, was highly upregulated in kappa- and iota-carrageenan (Table 1, Supplementary Data 1-9) and therefore stood out as a top candidate. To test this hypothesis we cloned 10 different *Z. galactanivorans* GH2 constructs, including ZGAL_3633 (Supplementary Figure 1). All the purified enzymes showed beta-galactosidase activity on the artificial substrate pNP-beta-D-galactose (Supplementary Fig. 11); however, only ZGAL_3633 and ZGAL_4655 demonstrated activity on furcellaran oligosaccharides pre-incubated with ZGAL_3152 (see below, and Fig. 7).

The furcellaran oligosaccharides were produced by incubating furcellaran with the kappa-carrageenase from *Pseudoalteromonas carrageenovora*[11] to produce even-DP oligosaccharides with 3,6-anhydro-D-galactose residues on the non-reducing terminus and D-galactose-4-sulfate on the reducing terminus. Furcellaran oligosaccharides were incubated with the 3,6-anhydro-D-galactosidase ZGAL_3152 and then separated chromatographically by HPAEC (Fig. 7), resulted in the loss of peaks P6 and P11 and the appearance of P2, P3 and P9 (odd-DP oligosaccharides). Incubation of the ZGAL_3152 pre-treated oligosaccharides with the GH2 enzymes ZGAL_3633 and ZGAL_4655 resulted in the depletion of P2 and the appearance of P1. As a standard we also ran a simple mixture of kappa-carrageenan oligosaccharides. A kappa-DP2 (with one sulfate) elutes at the same time as P1, a kappa-DP4 (with two sulfates) elutes at the same time as P5. Thus, P2 and P3, which are produced by the activity of ZGAL_3152, must be odd number oligosaccharides between DP2 and DP4, hence, a trisaccharide. This also supports that P1 is kappa-DP2. Based on this reasoning, we hypothesize that P2 is the following trisaccharide: D-galactose-beta-(1,4)-3,6-anhydro-D-galactose-alpha-(1,3)-D-galactose-4-sulfate. Thus, treatment with ZGAL_3633 and ZGAL_4655 results in the products D-galactose and kappa-DP2 (P1). The furcellaran oligosaccharides are a complex mixture of sugars from a natural source, the trisaccharide P3 likely has the same backbone structure as P2, but may be modified even further, such as with another sulfate moiety, a methyl or a pyruvate group.

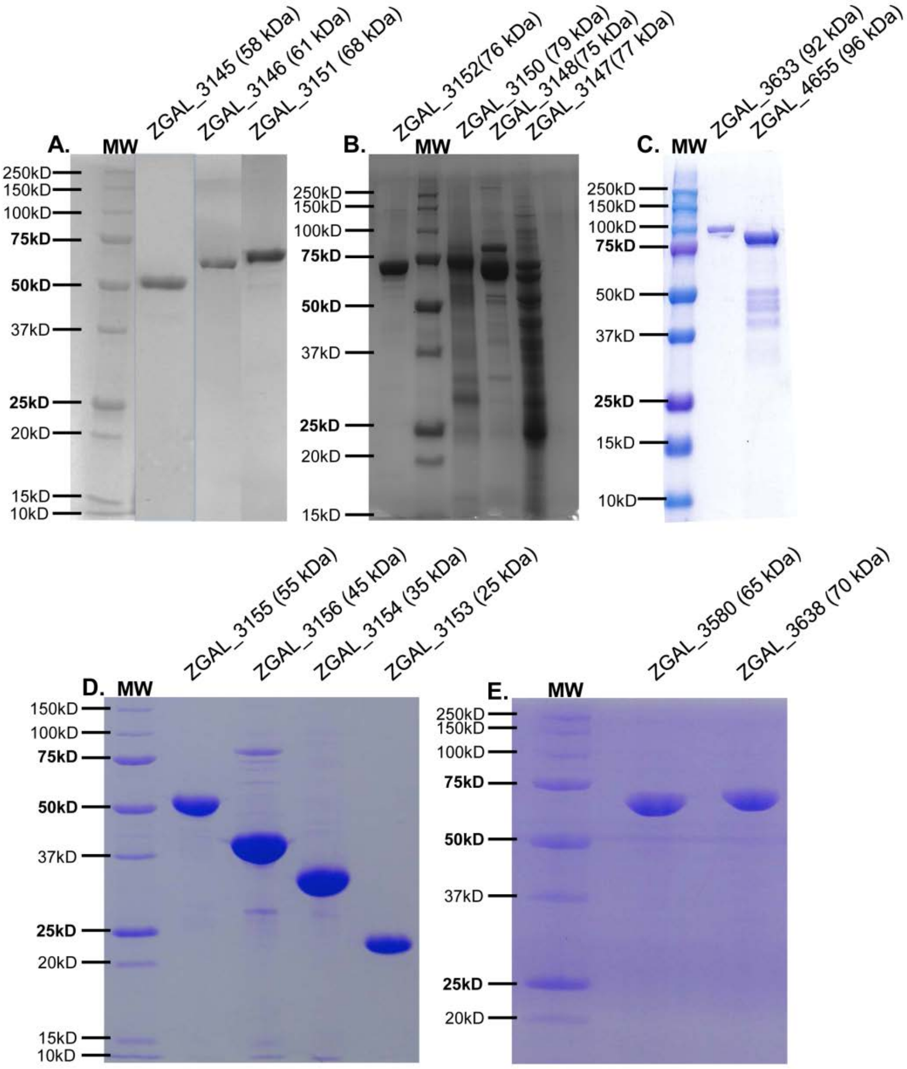

Supplementary Figure 1. Soluble proteins produced from the ZGAL_3145-ZGAL_3159 locus and select proteins whose genes are found distal to the locus after purification by nickel-affinity chromatography. For each protein, the expected size is given in parentheses. MW: molecular weight marker. **(A)** The carrageenan PUL sulfatase enzymes. **(B)** The 3,6-anhydro-D-galactosidases. **(C)** The GH2 enzymes active on furcellaran oligosaccharides. **(D)** The enzymes involved in conversion of D-AnG into D-glyceraldehyde-3-phosphate and pyruvate. **(E)** The SusD-like proteins induced by kappa- and iota-carrageenan.

A.

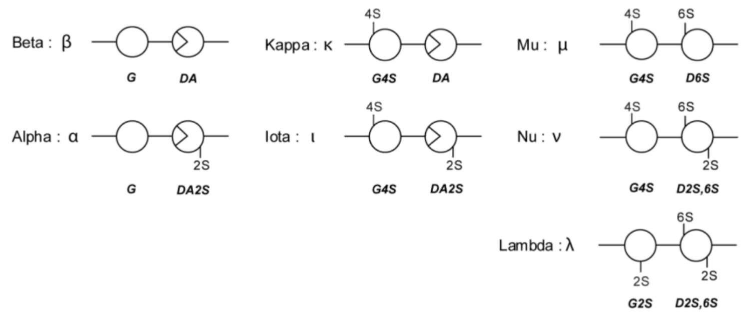

B.	SUBSTRATES								
Sulfatases	Polymers						Oligosaccharides		
	κ/μ	κ	ι/ν	ι	λ	α	κ	ι	ι/α
**ZGAL_3145**	0	5	372	276	0	10	-	125	0
**ZGAL_3146**	4	28	2	0	0	2	0	-	-
**ZGAL_3151**	3	1	14	0	0	7	0	-	114

C.

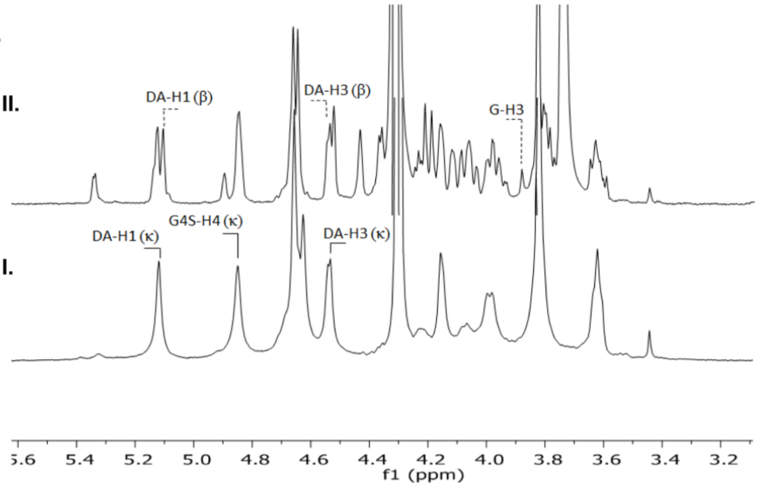

Supplementary Figure 2. (A) Nomenclature of the main carrageenan structures according to Knutsen and coworkers[6]. **(B)** The 3 sulfatases ZGAL_3145 (CgsA), ZGAL_3146 (CgsB1) and ZGAL_3151 (CgsC) were screened against a carrageenan collection consisting of polymers and oligosaccharides. After incubation, filtrates were analyzed by HPAEC revealing the amount of free sulfate produced during the reaction. Data is expressed in µg of sulfate released by ml of reaction mixture. **(C)** ^{1}H-NMR spectrum of kappa-carrageenan before (spectrum I) and after (spectrum II) incubation with the sulfatase ZGAL_3146 (CgsB1), resulting in the production of hybrid kappa/beta carrageenan.

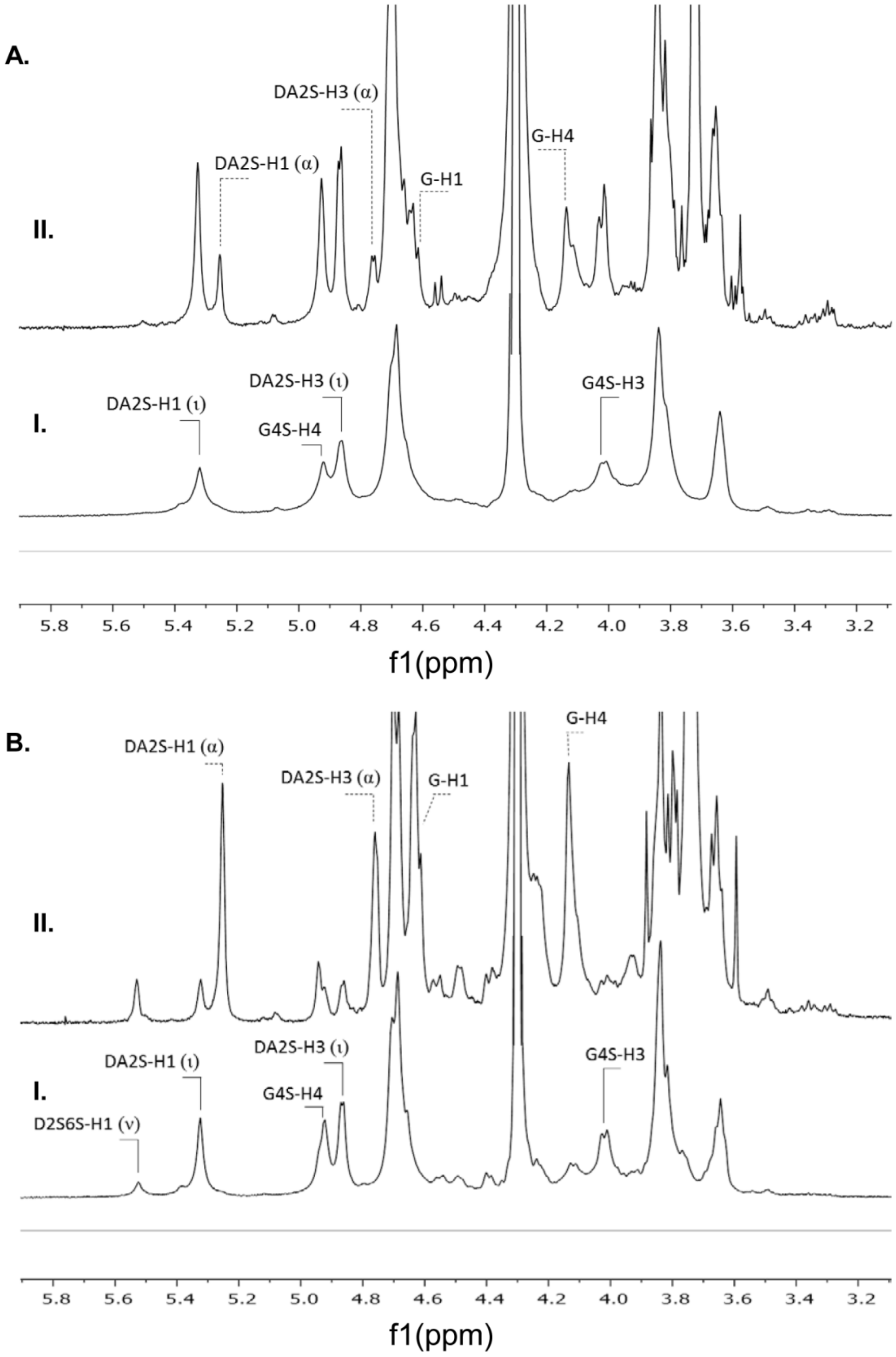

Supplementary Figure 3. ^{1}H-NMR spectrum of iota-carrageenan **(A)** or hybrid iota/nu-carrageenan **(B)** before (spectrum I) and after (spectrum II) incubation with the sulfatase ZGAL_3145 (CgsA), resulting in the conversion of the substrate into alpha-carrageenan (AII) or hybrid alpha/nu-carrageenan (BII).

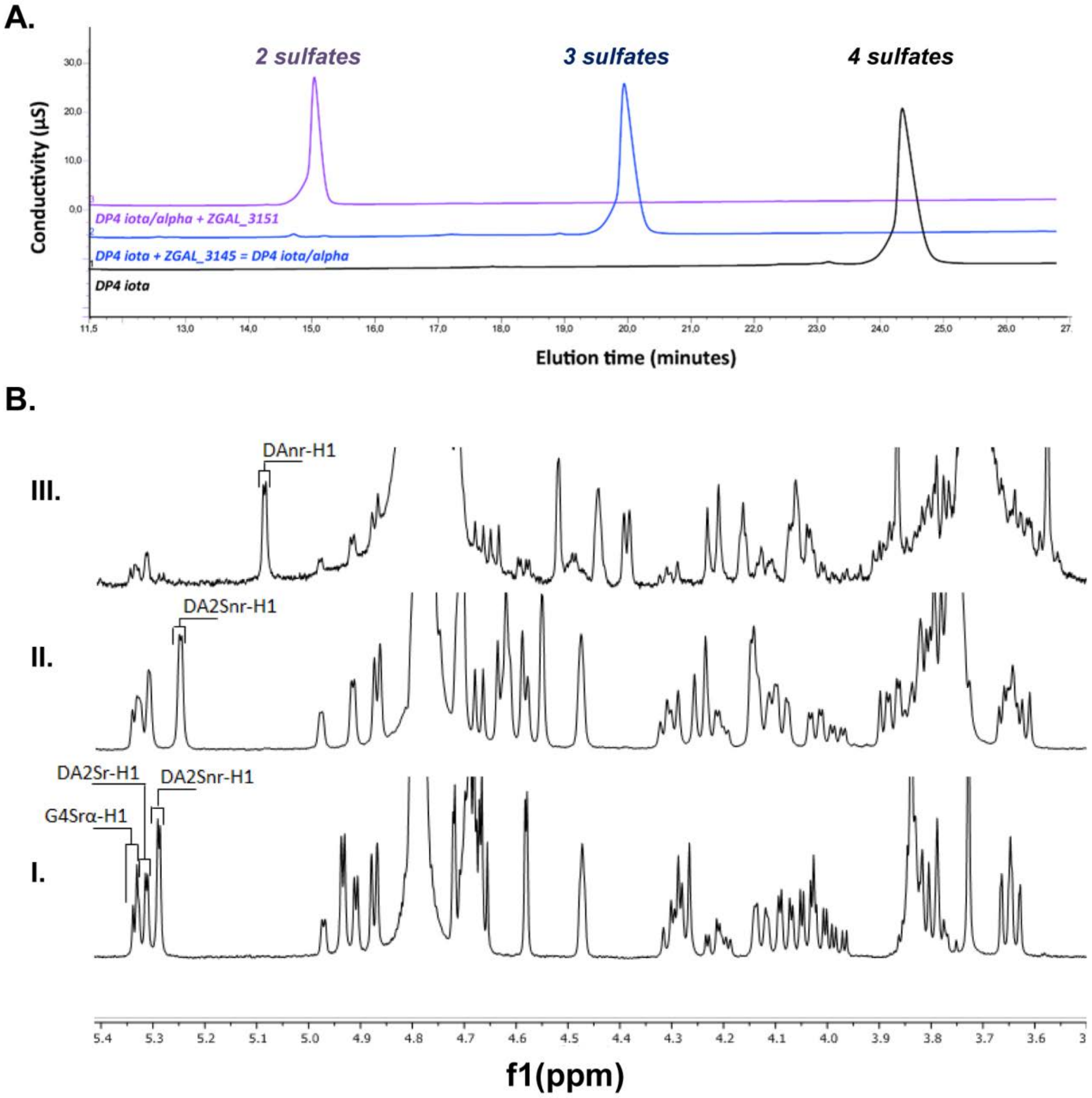

Supplementary Figure 4. Sequential action of the sulfatases ZGAL_3145 (CgsA) and ZGAL_3151 (CgsC) on iota-carratetraose (DP4). (A) HPLC profile of DP4 iota before (black line) and after treatment with ZGAL_3145 (blue line) resulting in the loss of one sulfate. The alpha/iota DP4 oligosaccharide produced by ZGAL_3145 was then incubated with ZGAL_3151 (violet line) resulting in the removal of one more sulfate. **(B)** ^{1}H-NMR spectrum of iota-carratetraose (DP4) (I) incubated with the sulfatase ZGAL_3145 which produces alpha-carrageenan on the non-reducing (nr) end (II) followed by treatment with ZGAL_3151 (III) which produces beta-carrageenan on the nr end.

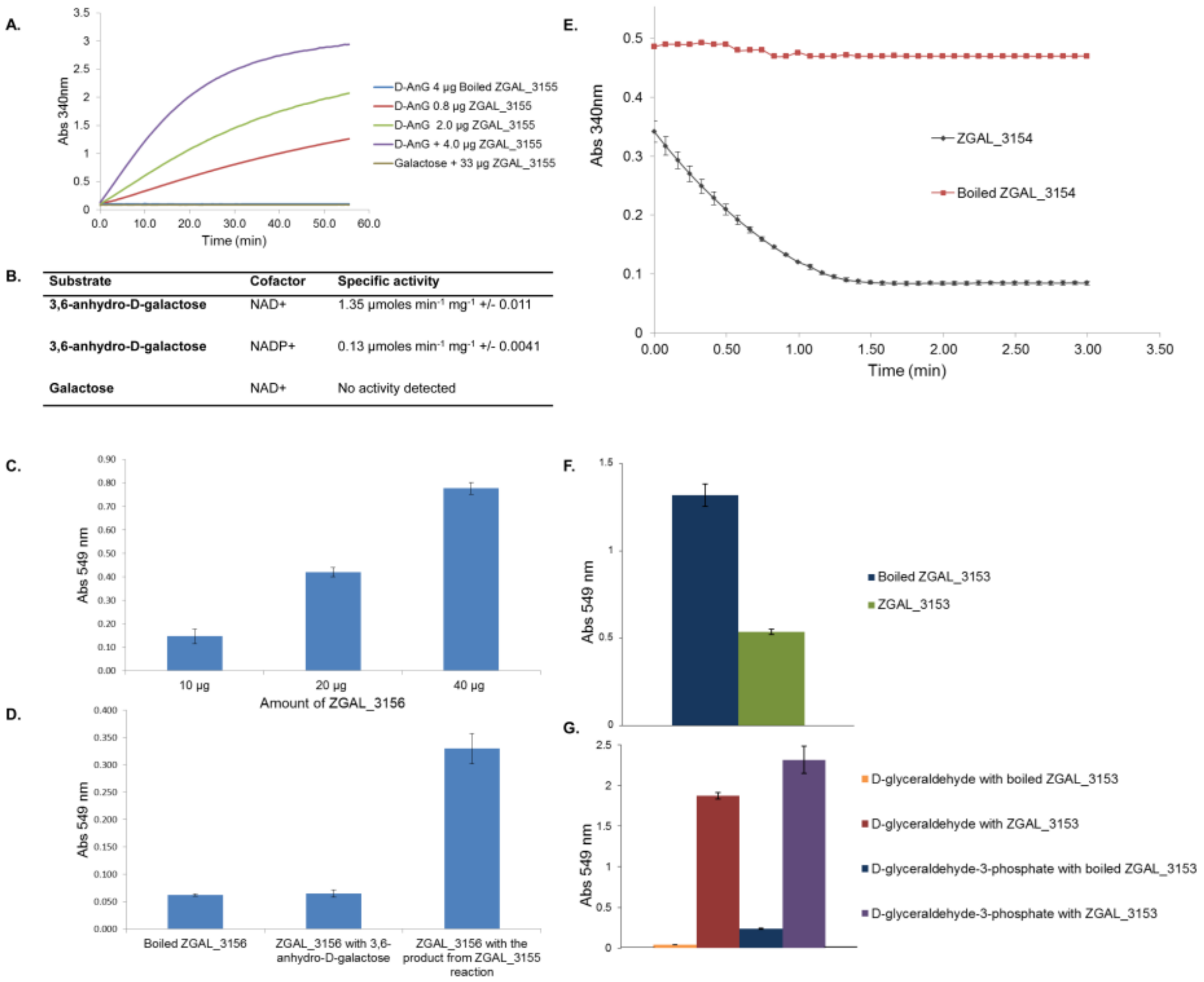

Substrate	Cofactor	Specific activity
3,6-anhydro-D-galactose	NAD+	1.35 µmoles min^{-1} mg^{-1} +/- 0.011
3,6-anhydro-D-galactose	NADP+	0.13 µmoles min^{-1} mg^{-1} +/- 0.0041
Galactose	NAD+	No activity detected

Supplementary Figure 5 (the legend is on the next page).

Supplementary Figure 5. Biochemical characterization of enzymes processing 3,6-anhydro-D-galactose (DauA-D). (A) Activity test for ZGAL_3155 (DauA, 3,6-anhydro-D-galactose dehydrogenase), determined spectrophotometrically as a function of the reduction of NAD^+. Different amounts of enzyme (0.8-4 µg) were added to 10 mM 3,6-anhydro-D-galactose (D-AnG) that was converted to 3,6-anhydro-D-galactonate. Controls with boiled enzyme on D-AnG or fresh enzyme on D-galactose showed no activity. **(B)** Activity of ZGAL_3155 (DauA) as a function of the substrate and cofactor (mean ± s.d, n=3). **(C)** Activity test for ZGAL_3156 (DauB, 3,6-anhydro-D-galactonate cycloisomerase), on the product of ZGAL_3155. The conversion of 3,6-anhydro-D-galactonate into 2-keto-3-deoxy-D-galactonate was determined using the thiobarbituric assay (TBA), with various amounts of purified recombinant enzyme (10-40 µg). Values are mean ± s.d (n=3). **(D)** Comparison of the activity of ZGAL_3156 on 3,6-anhydro-D-galactose or the product from ZGAL_3155 reaction, measured using the TBA assay. A control with only boiled ZGAL_3156 was performed. All reactions were conducted with 50 µg of purified recombinant enzyme. Values are mean ± s.d (n=3). **(E)** Activity test for ZGAL_3154 (DauC, 2-keto-3-deoxy-D-galactonate kinase). Reactions contained 2 µg of pure recombinant enzyme and the product of ZGAL_3156. The conversion of 2-keto-3-deoxy-D-galactonate into 2-keto-3-deoxy-6-phospho-D-galactonate was determined indirectly as a function of the oxidation of NADH. A control with boiled enzyme was performed for comparison. Values are mean ± s.d (n=3). **(F)** Activity test for ZGAL_3153 (DauD, 2-keto-3-deoxy-D-galactonate aldolase) in the forward direction. The TBA assay was used to measure the degradation of 2-keto-3-deoxy-6-phospho-D-galactonate (product of ZGAL_3154) into D-glyceraldehyde-3-phosphate and pyruvate by 2 µg of purified recombinant ZGAL_3153. A control with the same amount of boiled enzymes was performed for comparison. Values are mean ± s.d (n=3). **(G)** Activity test for ZGAL_3153 in the reverse direction. The synthesis of 2-keto-3-deoxy-6-phospho-D-galactonate from pyruvate (50 mM) and D-glyceraldehyde-3-phosphate or D-glyceraldehyde (20 mM) with 5 µg of purified recombinant enzyme was measured using the TBA assay. Controls with the same amount of boiled enzymes were performed for comparison. Values are mean ± s.d (n=3).

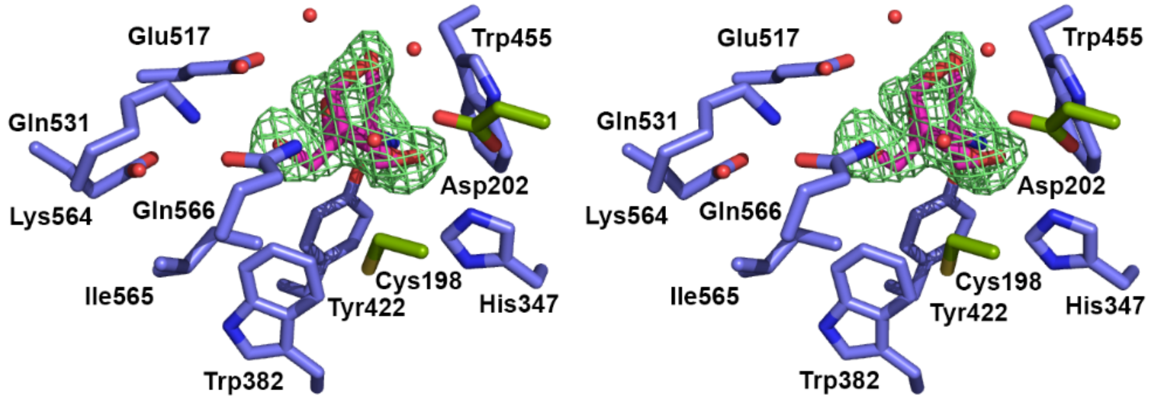

Supplementary Figure 6. Omit map stereo image of the X-ray crystal structure of the active site of ZGAL_3152 (DagB). The omit map was generated by omitting the Tris molecule (in pink) within the active sites of monomer A and B from the refinement. The resulting maps are maximum-likelihood σ_A-weighted F_{obs}-F_{calc} maps contoured in green at 2σ (0.09 $e^-/Å^3$). Active site residues from monomer A are coloured lime green. Active site residues from monomer B are coloured in blue-violet. Three ordered water molecules within the active site are represented by red spheres.

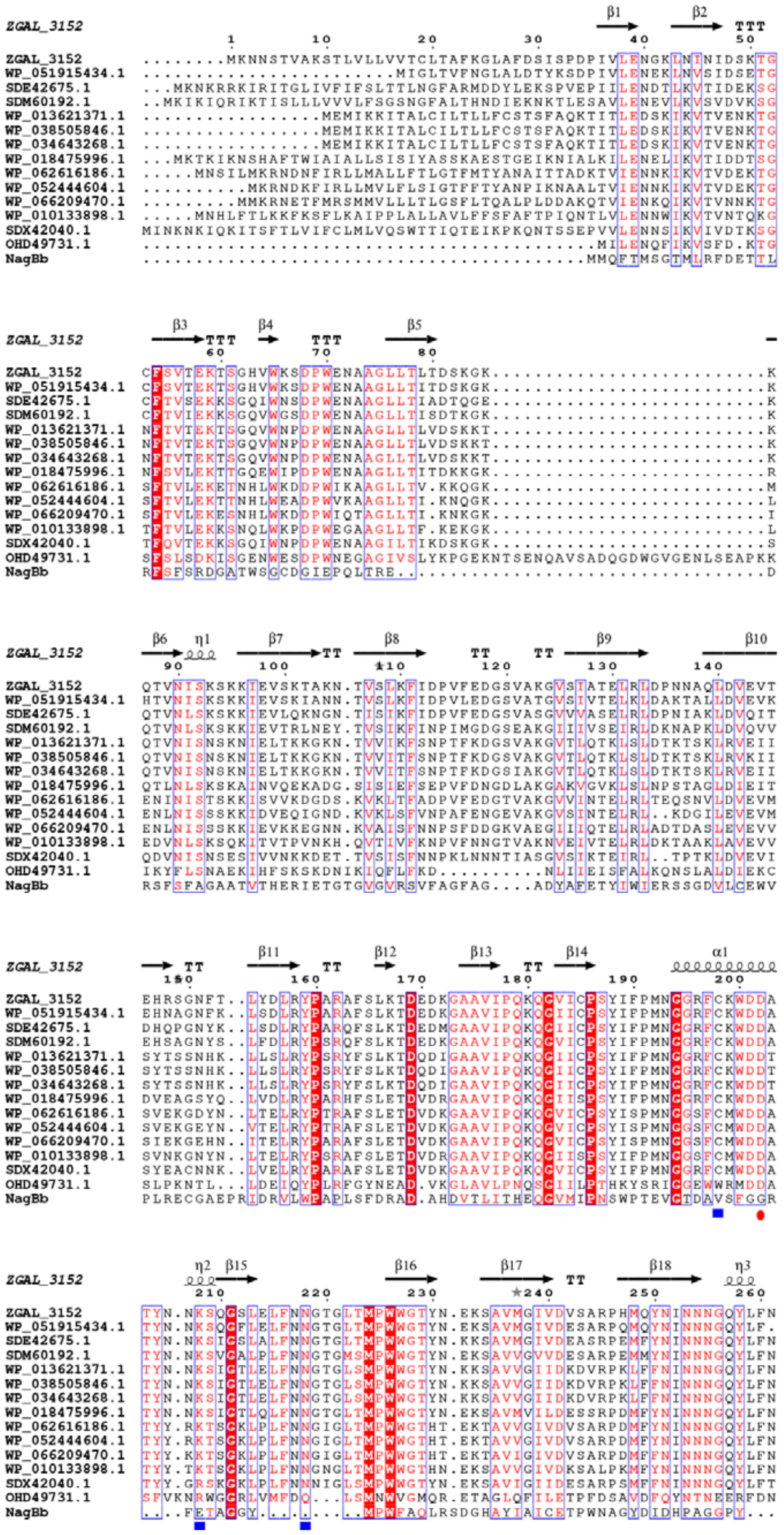
ZGAL_3152
WP_051915434.1
SDE42675.1
SDM60192.1
WP_013621371.1
WP_038505846.1
WP_034643268.1
WP_018475996.1
WP_062616186.1
WP_052444604.1
WP_066209470.1
WP_010133898.1
SDX42040.1
OHD49731.1
NagBb

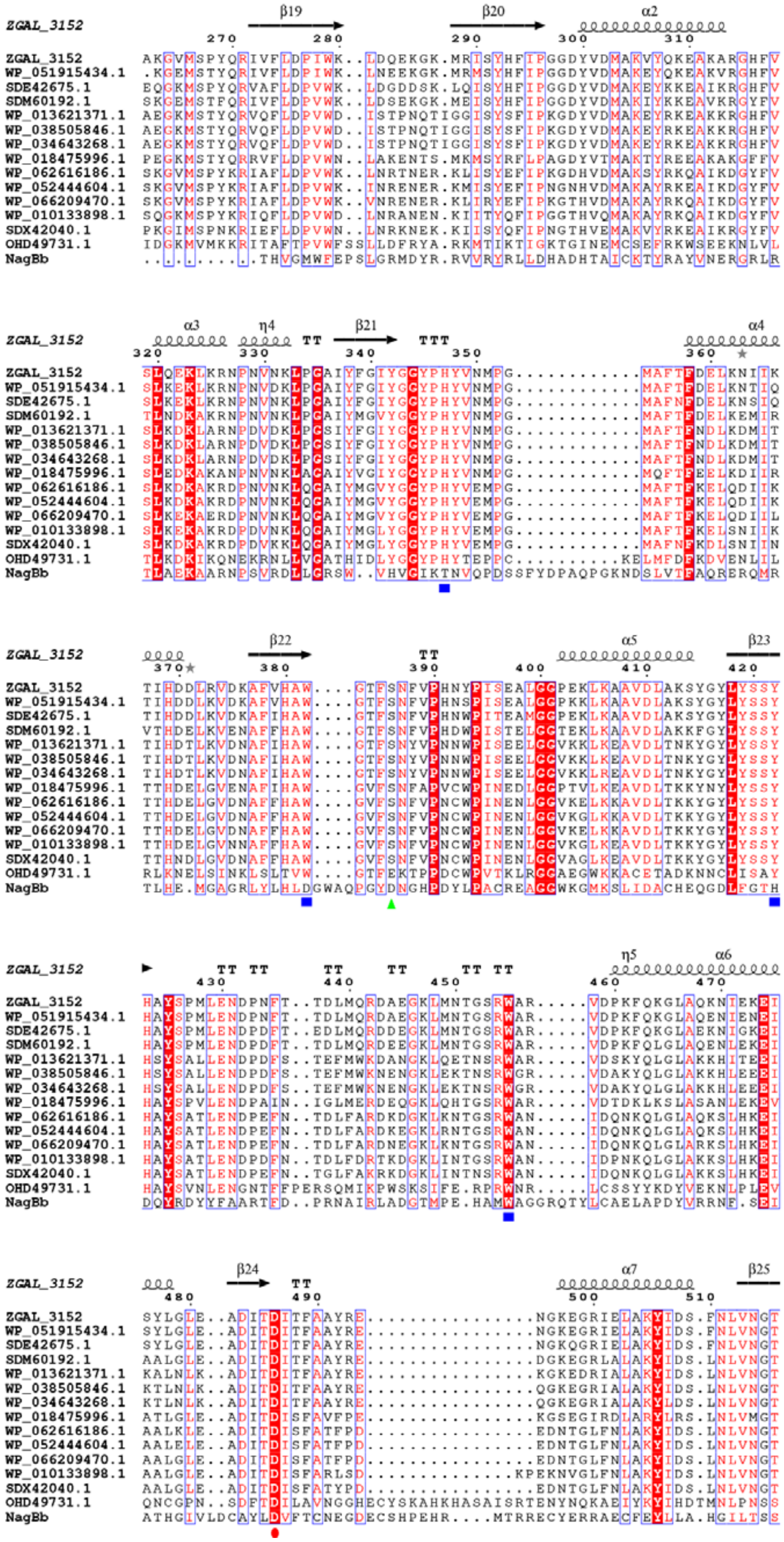

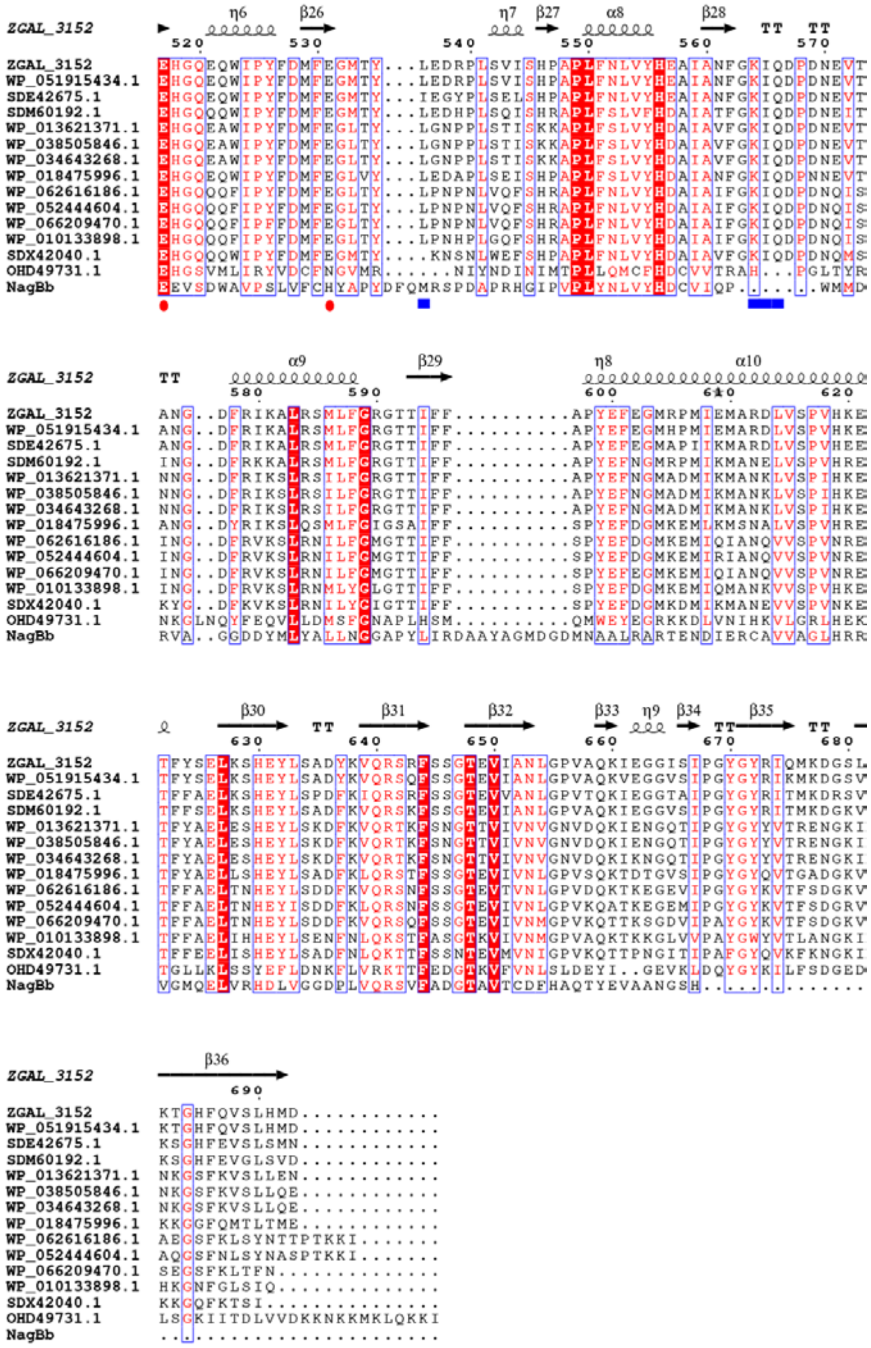

Supplementary Figure 7. Sequence alignment of the GH129-like α-1,3-(3,6-anhydro)-D-galactosidase DagB (ZGAL_3152) with its close homologues and with the family GH129 alpha-N-acetylgalactosaminidase NagBb from *Bifidobacterium bifidum*. The secondary structure representation of ZGAL_3152 is shown above the sequence alignment. The α-helices and β-strands are represented as *helices* and *arrows*, respectively, and β-turns are marked with *TT*. *Dark shaded boxes* enclose invariant positions, and *light shaded boxes* show positions with similar residues. Blue squares indicate the residues involved in the binding of Tris and MDP molecules in the active site (potentially responsible for substrate recognition). Red circles mark the potential catalytic residues of ZGAL_3152. The green triangle indicates the catalytic "fixer" residue of NagBb (Asp330)[3]. The figure was prepared by ESPript 3.0 (http://espript.ibcp.fr)[12].

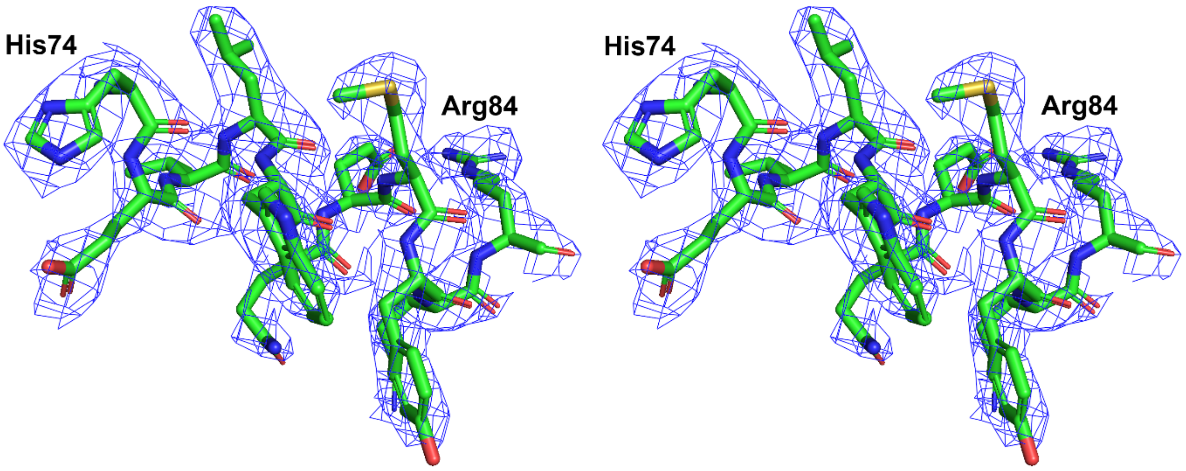

Supplementary Figure 8. 2Fo-Fc map stereo image of the X-ray crystal structure of the active site of ZGAL_3156 (DauB). The 2Fo-Fc map is contoured in blue at 1σ. The residues from His74 to Arg84 are represented as sticks coloured in green.

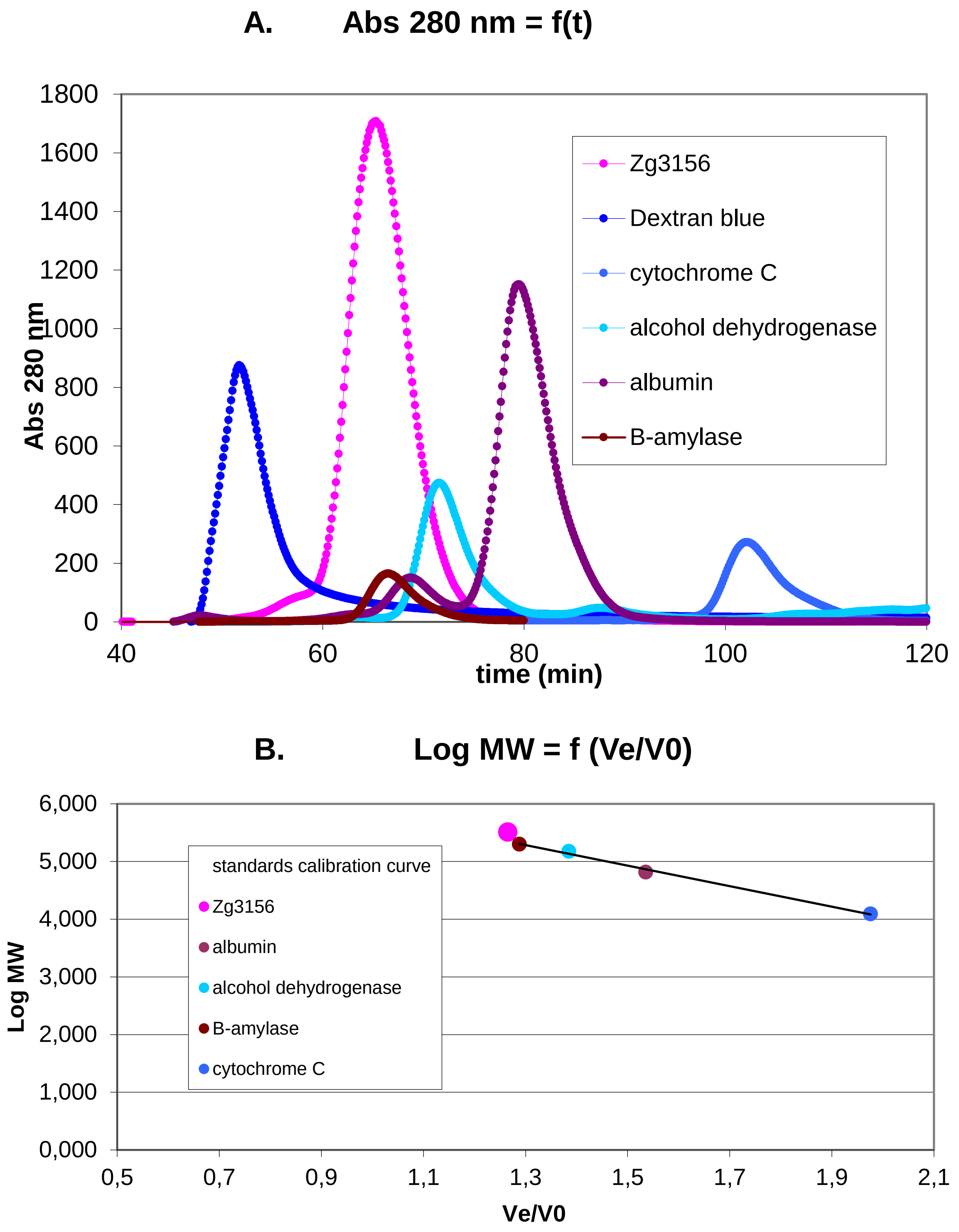

Supplementary Figure 9. A. Size exclusion chromatography (Superdex 200, GE healthcare) of the 3,6-ahydro-D-galactonate cycloisomerase ZGAL_3156. This chromatogram is superposed with the chromatograms of standard biomolecules analyzed in identical conditions (dextran blue: 2000 kDa; beta-amylase: 200 kDa; alcohol dehydrogenase: 150 kDa; albumin: 66 kDa; cytochrome C: 1.24 kDa). B. Superdex 200 calibation curve. MW: molecular weight (Da); Ve: Elution volume; V0: void volume. The measured molecular weight of ZGAL_3156 is 327024 Da (monomer theoretical MW: 44690 Da) indicating a octameric form in solution (octamer theoretical MW: 357520 Da).

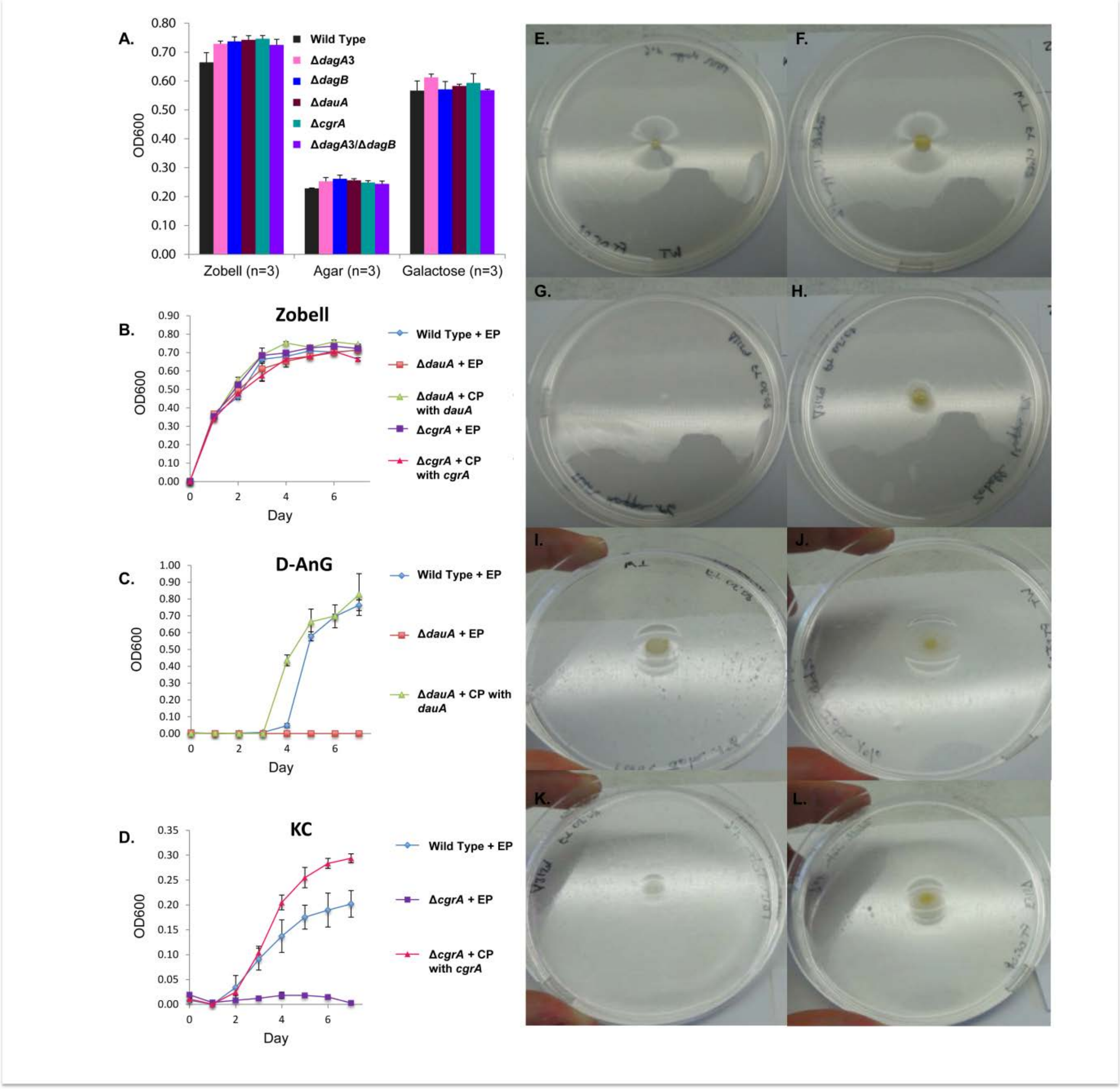

Supplementary Figure 10. Phenotyping of *Z. galactanivorans* mutant and complemented strains for genes in the carrageenolytic PUL. (A) Average growth of wild type *Z. galactanivorans* and deletion mutants on Zobell media, agar and D-galactose on day three of growth. **(B)** Growth curve for complemented wild type and mutant strains Δ*dauA* and Δ*cgrA* in Zobell medium. The *Z. galactanivorans* genome has been modified distal to the carrageenan PUL incorporating an EP (empty plasmid) or CP (complementation plasmid containing the complemented target gene). **(C)** Growth curve for complemented wild type and mutant strains Δ*dauA* (Δ*zgal_3155*, Δ*3,6-anhydro-D-galactose dehydrogenase*) in minimum medium supplemented with 3,6-anhydro-D-galactose (D-AnG). **(D)** Growth curve for complemented wild type and mutant strains Δ*cgrA* (Δ*zgal_3159*, Δ*araC family regulator*) in minimum medium supplemented with kappa-carrageenan (KC). In panels A-D, error bars represent standard error of the mean between three replicates. **(E-H)** Growth of the wild type (E, F) and Δ*cgrA* (G, H) strains on gels containing kappa-carrageenan as a sole carbon source (E, G) or kappa-carrageenan supplemented with Zobell (F, H). The degradation of kappa-carrageenan was evidenced by a depression in the gel (E, F). **(I-L)** Growth of the wild type (I,J) and Δ*cgrA* (K, L) strains on gels containing iota-carrageenan as sole carbon source (L, K) or iota-carrageenan supplemented with Zobell (J, L). The degradation of iota-carrageenan was evidenced by a liquefaction of the gel. See supplementary discussion section 3 for an extended discussion on these results.

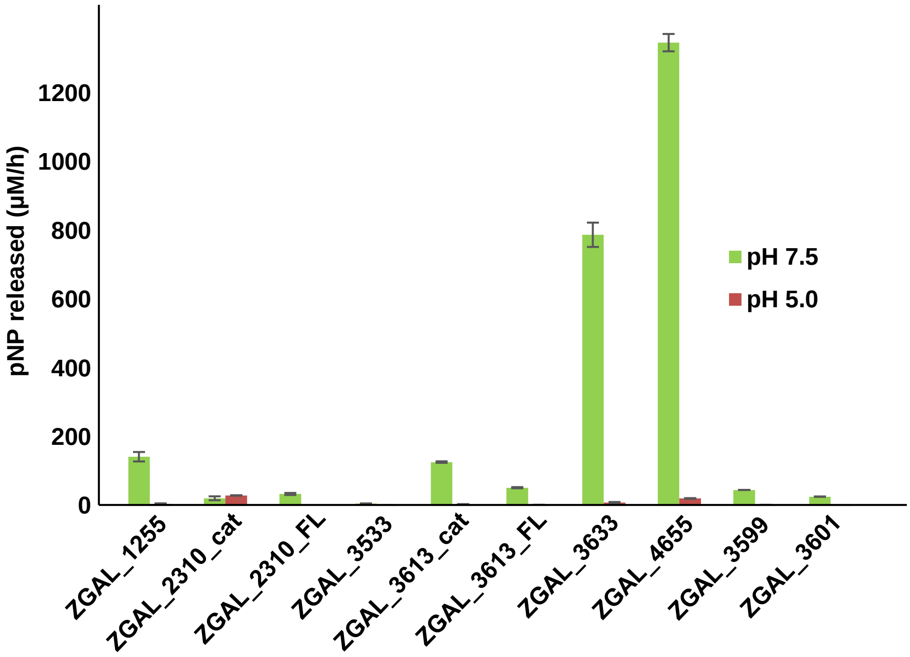

Supplementary Figure 11. Activity test of the 10 GH2 polypeptide constructs at 0.1 µg/µL on 2.5 mM pNP-β-galactopyranoside. Activities are expressed in µM of pNP released per hour on kinetics realised in 0.1M sodium acetate pH 5.0 and 0.1M Hepes pH 7.5 followed by spectrophotometry (410 nm). FL refers to the full length enzyme and cat refers to just the catalytic module of the enzyme.

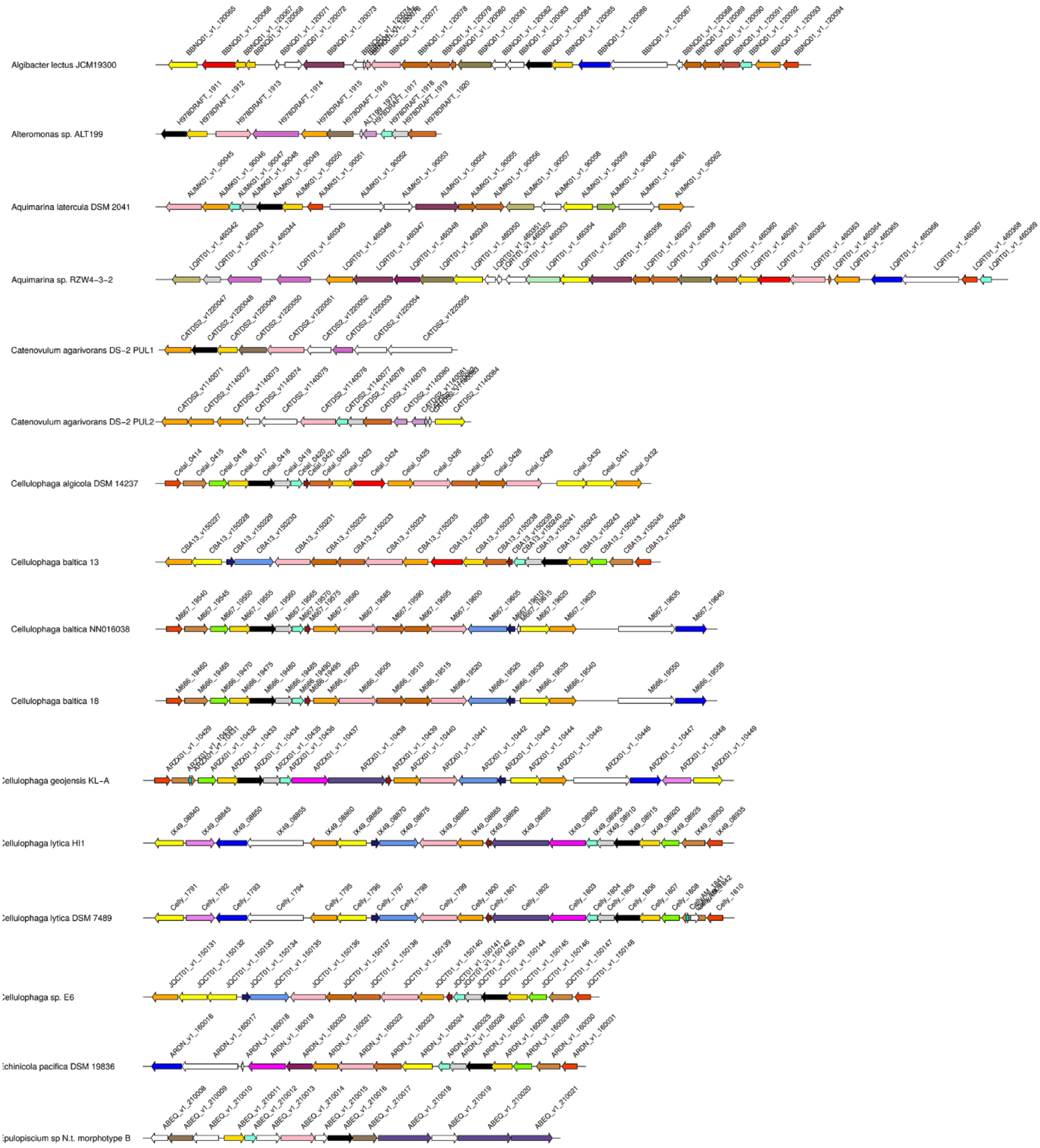
Algibacter lectus JCM19300
Alteromonas sp. ALT199
Aquimarina latercula DSM 2041
Aquimarina sp. RZW4-3-2
Catenovulum agarivorans DS-2 PUL1
Catenovulum agarivorans DS-2 PUL2
Cellulophaga algicola DSM 14237
Cellulophaga baltica 13
Cellulophaga baltica NN016038
Cellulophaga baltica 18
ellulophaga geojensis KL-A
ellulophaga lytica HI1
ellulophaga lytica DSM 7489
ellulophaga sp. E6
chinicola pacifica DSM 19836
pulopiscium sp N.t. morphotype B

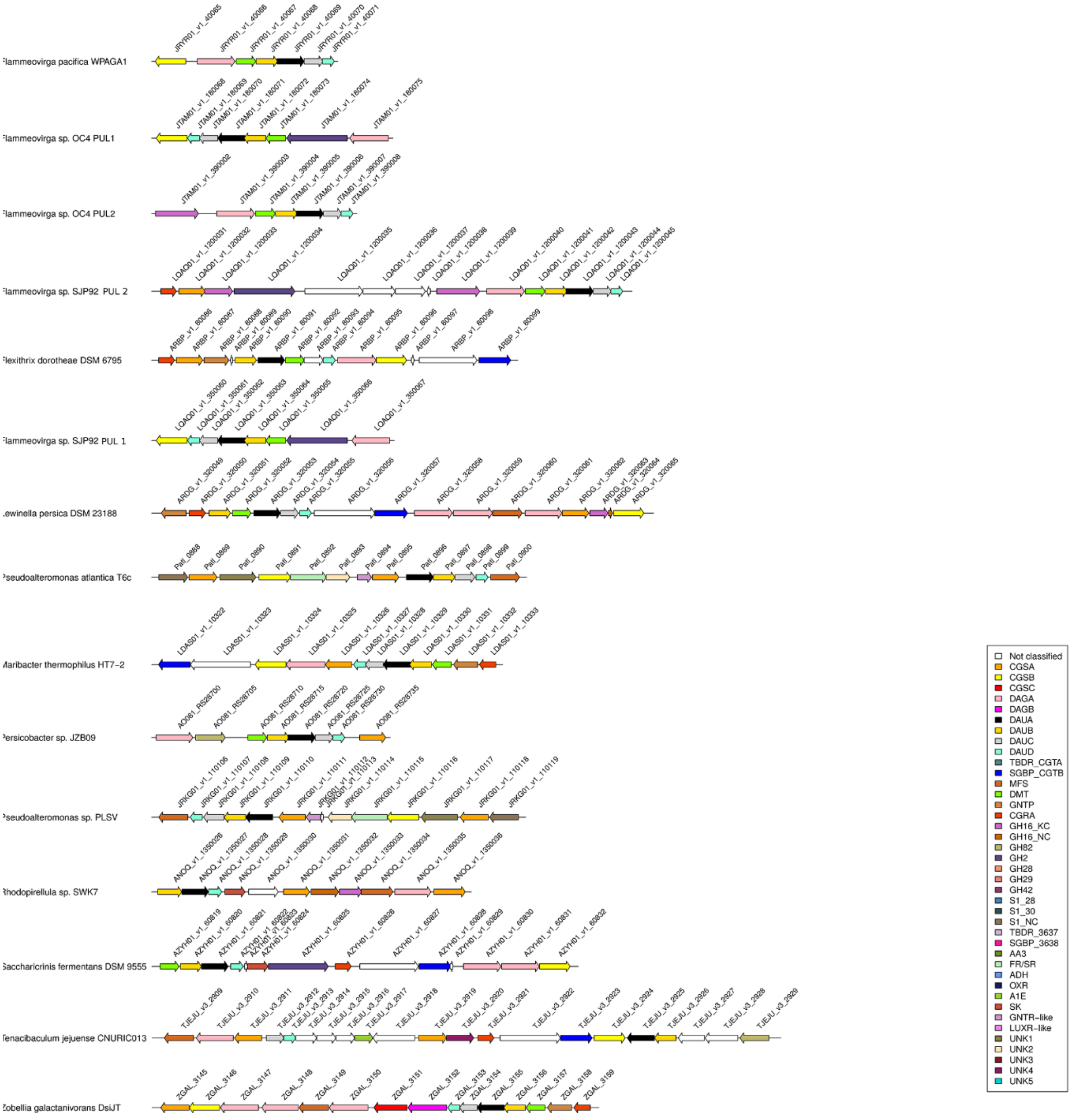

Supplementary Figure 12. Gene composition of the carrageenan-specific PULs that were used in the clustering experiment and to generate the heatmap in Figure 9.

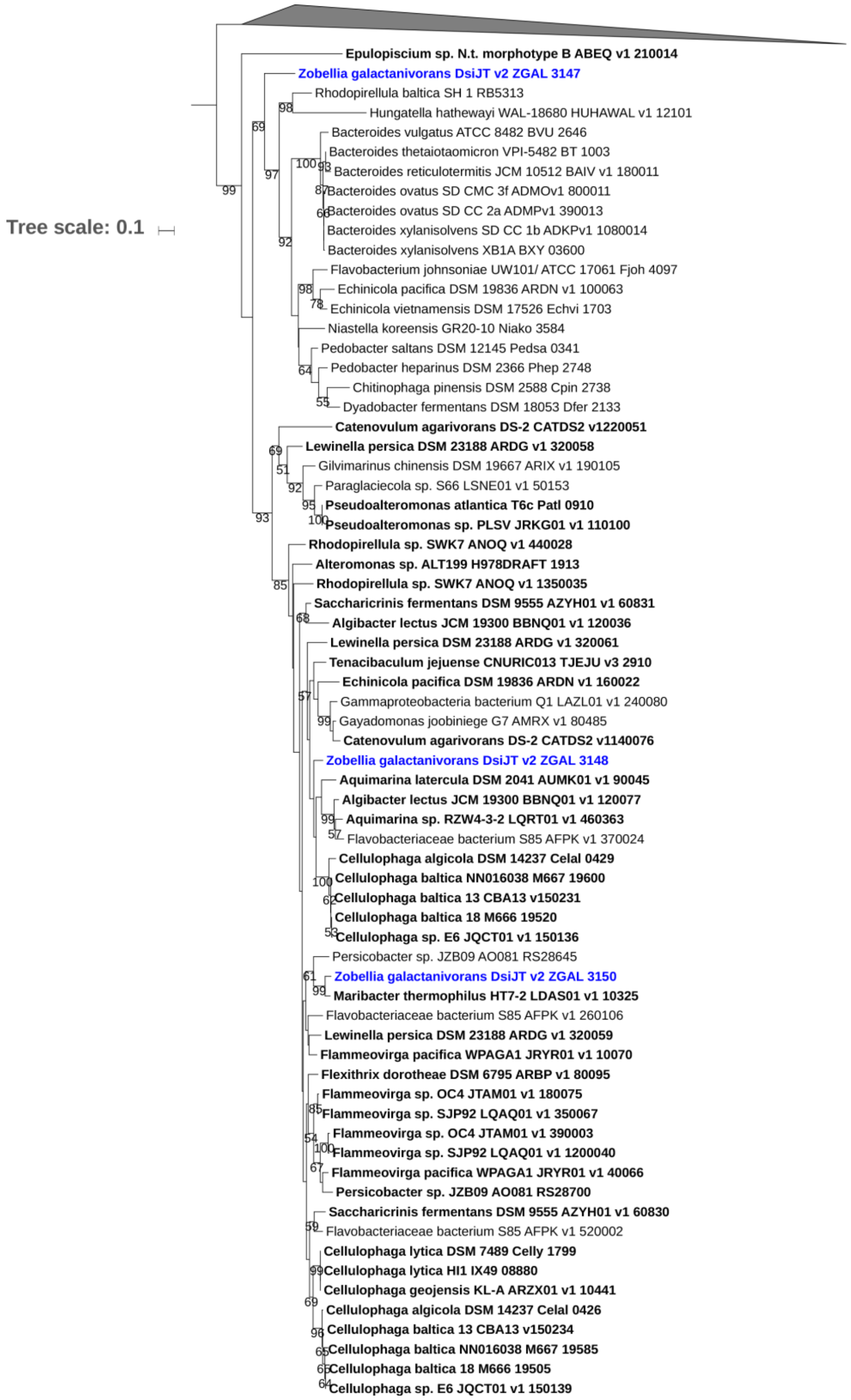

Supplementary Figure 13. **Phylogenetic relationship of GH127 (DagA) domains close to *Zobellia galactanivorans* GH127 PUL proteins.** The tree was generated with 549 domain protein sequences using a maximum likelihood method with 100 bootstrap replicates. Only the clade gathering *Z. galactanivorans* PUL sequences is represented here, the rest of the tree being collapsed. This clade is supported by 99% bootstrap. Bootstrap values superior or equal to 50% are shown. *Zobellia galactanivorans* GH127 PUL proteins are highlighted in blue. Proteins belonging to a similar PUL structure are highlighted in bold. The represented clade is divided into Clade 1, which includes only sequences from marine bacteria, and Clade 2, which contains sequences from bacteria originating from marine, human gut and soil ecosystems.

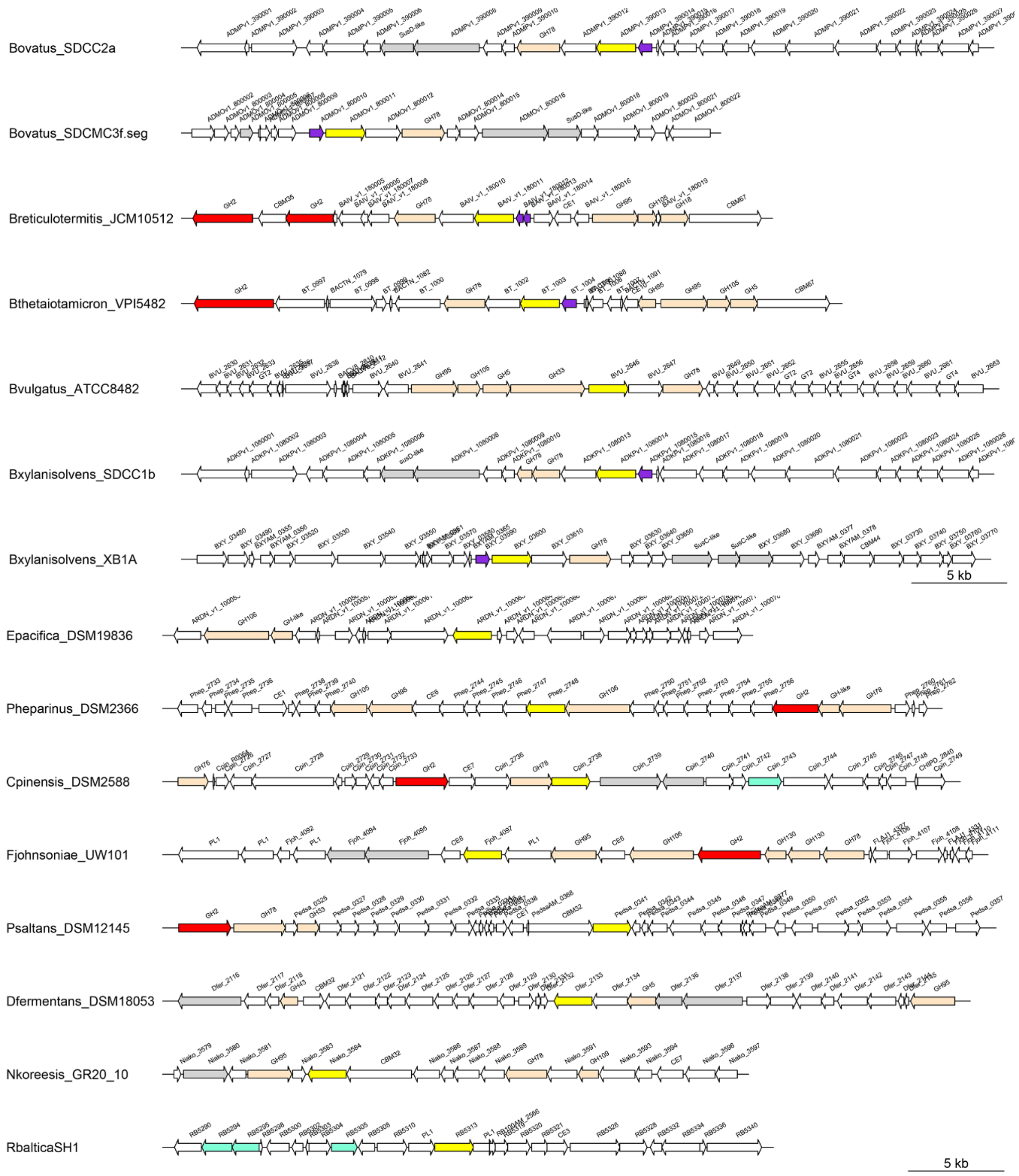

Supplementary Figure 14. Genomic context of the clade 2 enzymes of the family GH127 (DagA) phylogenetic tree represented in Supplementary Figure 11. The genes encoding GH127, GH2 and GH from other families are colored in yellow, red and bisque, respectively. The phage/transposase genes are colored in blueviolet. The *susCD*-like gene pairs are colored in light grey.

Supplementary Table 1. X-ray crystallography data collection statistics.

	ZGAL_3152*	ZGAL_3156*
**Data collection**		
Space group	C121	P21
Cell dimensions		
a, *b*, *c* (Å)	222.00 107.50 165.80	85.84 154.07 150.87
α, β, γ (°)	90.00 114.00 90.00	90.00 104.38 90.00
Resolution (Å)	46.70-1.70 (1.74-1.70)	49.17-2.79 (2.87-2.79)
R_{sym} or R_{merge}	4.8 (38.3)	22.7 (129.1)
$I / \sigma I$	16.1 (3.1)	3.97 (0.87)
Completeness (%)	99.6 (98.5)	99.0 (95.6)
Redundancy	3.1 (3.1)	3.6 (3.6)
CC1/2	99.9 (83.2)	97.8 (47.3)
**Refinement**		
Resolution (Å)	46.70-1.70 (1.74-1.70)	49.17-2.79 (2.87-2.79)
Total reflections	1226588	333244
Unique reflections	389746	93118
R_{work} / R_{free}	14.7/17.6	23.1/26.6
No. atoms		
Protein Chain A	5244	2721
Protein Chain B	5256	2709
Protein Chain C	5224	2717
Protein Chain D	5228	2711
Protein Chain E		2735
Protein Chain F		2741
Protein Chain G		2730
Protein Chain H		2745
Metal ions	4	5
Water	2841	92
B-factors		
Protein Chain A	18.4	70.6
Protein Chain B	18.8	71.1
Protein Chain C	18.7	71.7
Protein Chain D	19.6	71.7
Protein Chain E		66.6
Protein Chain F		66.3
Protein Chain G		67.3
Protein Chain H		66.7
Metal ions	23.5	72.2
Water	29.5	43.7
R.m.s. deviations		
Bond lengths (Å)	0.024	0.010
Bond angles (°)	2.289	1.19

* Data was collected on one crystal for each protein. R_{merge} data for for ZGAL_3156 is not relevant as it was collected on an Eiger detector.

Supplementary Table 2. Primer sequences used for cloning genes for recombinant protein production.

Gene Locus	*Nucleotides*	*Forward primer sequence*	*Reverse primer sequence*
ZGAL_1255	67-2844	AAAAAAGGATCCATGCAATCGAATACGGAACCCAAC	TTTTTTCTGCAGCTAAAATTCAAAGCTCAGATGTATAGT
ZGAL_1920	85-3210	AAAAAAGGATCCCAGGCGCCTAAATGGGAAAATCC	TTTTTTCTCGAGCTATTTCACCGGTTGTATCATAAAAC
ZGAL_2310	85-3081	AAAAAAGGATCCAGTCCGCCCAAATCGGACAAGC	TTTTTTCTGCAGTTAACGAACTGAAATCTCGTCAATAAA
ZGAL_2310cat	85-2445	AAAAAAGGATCCAGTCCGCCCAAATCGGACAAGC	TTTTTTCTGCAGTTAAGTGATTAATTTTTTCTCTCCCAAGAT
ZGAL_3145	130-1531	AAAAAAGGATCCCAAGAAACACGTCCGAACATCCTTG	TTTTTTGAATTCTTATTTGTGATAAGGACTGTCGACTA
ZGAL_3146	55-1632	TTTTTTAGATCTTGCAGCTCGGCACAAAACAGTATC	AAAAAAGAATTCTTAGTTTAGGGAATGTTGCTCTTTTTC
ZGAL_3147	64-2041	TTTTTTGGATCCCACGGGGTATTAAACAACGCCAAA	TTTTTTCAATTGTTAATATTGTAAAGGCAACCAAACGGAC
ZGAL_3148	4-1966	TTTTTTGTCGACAAACAAGCCACCCCTGCCAATAG	TTTTTTCTGCAGTTAATCGTTCCAAATTACAGGCATAAAT
ZGAL_3150	67-2080	TTTTTTCTCGAGAATCCGGACGCCTTAAAACACGAA	TTTTTTATGCATTTAATCCCAGATTACCGGTAAGAAAA
ZGAL_3151*	118-1788	AAAAAAAGATCTCGATCGCCCCAATGTATTGATCATG	TTTTTTCTCGAGTAGGTCCAATCGCTCAACATCCAC
ZGAL_3152	94-2077	TTTTTTGTCGACAGCCCAGACCCTATTGTTTTGGAG	TTTTTTCTGCAGTTAATCCATATGTAAACTCACTTGGAA
ZGAL_3153	4-643	TTTTTTGGATCCCAGAATAATTCATTTTCTTGGAAAAAATA	TTTTTTGAATTCTTATTCTTTTATAAAATCACGTATTTCATT
ZGAL_3154	4-949	TTTTTTGGATCCAAAATACCGAATACTTTTATTAGTTGTG	TTTTTTGAATTCTTATTCTGCATAGGTCTCCAGTATTT
ZGAL_3155	4-1447	TTTTTTCTCGAGGAGCAACTAAAATTCGGTCATAAAAAA	TTTTTTATGCATTCATTGGCGCAATAAGGCTTTGGT
ZGAL_3156	4-1162	TTTTTTGGATCCAAAATTAAAAAGATAGAACCCTATGTCA	TTTTTTGAATTCTTATGACCGAATTTTAGTTGCTCCAT
ZGAL_3533	70-2490	AAAAAAGGATCCGAAAATTCTAATGAACTAGAGGTAAAG	TTTTTTCTGCAGTTAAATTGATTTTGCCGAAACCGTGG
ZGAL_3580	78-1752	AAAAAACTCGAGGAAAGCTTGGATATAGACGTAAGGG	AAAAAAATGCATTAATATCCTTCGTTTTGAATAAGTGC
ZGAL_3599	64-3057	AAAAAAGGATCCACCGAAAAAGTCGACCTCCCCG	TTTTTTCTGCAGTTAGTTTCTATATCTCTCCTTGTTTCT
ZGAL_3601	52-2289	AAAAAAGGATCCTGTTCACCTAAAAAAGCTGATACGG	TTTTTTCTGCAGTTACTCTATGAGTTGTTCTGTAGTGG
ZGAL_3613	58-2913	AAAAAAAGATCTCAAAATGCAAACTTGCCAGAAGGTT	TTTTTTCTGCAGTTAATGCCACTCGCTCAAAATAAAAC
ZGAL_3613cat	58-2625	AAAAAAAGATCTCAAAATGCAAACTTGCCAGAAGGTT	TTTTTTCTGCAGTTAGGCATGATGCACCGTATTCAATTTG
ZGAL_3633	60-2478	AAAAAAGGATCCCAACTGAATACCGAGGCCGCTTA	TTTTTTCTGCAGTTACTTTGTCCTTACTATGATTTTTGAG
ZGAL_3638	96-1911	AAAAAACTCGAGAGCGATGACTTTTTAGATCAAACGAA	AAAAAAATGCATTATTGAACAAACTCGTCCCAATTCA
ZGAL_4655	51-2538	AAAAAAGGATCCTGTGGCCGCGAGCCGTCAAATG	TTTTTTCTGCAGCTAGTCCACTGCCTTGAGCTTCA

*Cloned into pET20b, all other constructs were cloned into pFO4

Supplementary Table 3. Primers used in the deletion and complementation study. Restriction sites in the primer sequences are underlined.

Primer	Sequence (5' → 3')	Restriction site
OFT0001	TTCAACAGTATTTTCGTCGTCCCC	PstI upstream
OFT0002	TCTGAAGGATCCGTCGTTTTCTTGGGCTTTGG	BamHI
OFT0003	TTTTTTGTCGACCAGCGTGCAGAGCGAAAGGGTT	SalI
OFT0004	TTTTTTGTCGACATGACGGTTTTCTTACCGGTAATCT	SaI
OFT0005	TAACAATTTGGGGTAAAATAGTGCC	
OFT0006	TTTACCGTCCCCGATATTAAAGCG	
ORL664	GAACAAGGATCCAATTTACGATATGGAGGAAAGGCT	BamHI
ORL665	CAAACAGTCGACAACCAAGAGTACAAGGGTGC	SalI
ORL666	TTACATGTCGACTAACAGACGTATTCTCCAGAATACAA	SalI
ORL667	CGAGCAGCATGCAGAGCGAAAGGGTTAATTTTTTC	SphI
ORL668	CGACGAAGAGGTCATCAAACATTG	
ORL669	GGAGCATTGTCGCCTAAGTGCC	
ORL670	ACCGGTGGATCCATAACATACGGTATTGTAATGGG	BamHI
ORL671	AGGGCAGTCGACTTCAAACGTTTTATTGTTAGAGG	SalI
ORL672	ATAGGGGTCGACTGCTCTACTTACGCGCTAGATGAC	SalI
ORL673	GGATAACTGCAGGTCGTAGAGGGTAAAATTGC	PstI
ORL674	ACGACCATACCCATATCGGGCG	
ORL675	AGTGGTAAAACCTTTAAAGTTTCCCT	
ORL676	TGCCCTGGATCCATTTACCGAAACGCCCCTAT	BamHI
ORL677	TTGTACGTCGACTTCATAGTCTATTTCTTTGGTTAATT	SalI
ORL678	ACTTCAGTCGACGTTCAAAGCCTTGATGAATATTACCC	SalI
ORL679	CAGTACCTGCAGTTTTATAGATTTACTTGTAGTGGA	PstI
ORL680	GTCGGTGCTTTAGGAAACGCTGG	
ORL681	CCGAATGGCCCGATTTTTTCGAC	
1978	GCTAGTCTAGAATGGAGCAACTAAAATTCGGTCATA	XbaI
1979	GCTAGGCATGCCAAGCGCAATCTGAAATTAGAGGTA	SphI
1980	GCTAGTCTAGAATGAAGTTGACGTACAAACAGACTG	XbaI
1981	GCTAGGCATGC GGAAGACACGTGAATTATCTCGTA	SphI
2088	GCTAGGAGCTCAAGATGTCACCGAACTCAAAATGG	SacI
2089	GCTAGACTAGTTCTCTTGATGGAGTTGCTTGGG	SpeI
2090	GCTAGACTAGTCCGCAATCAGAAATTAATAGAACTG	SpeI
2091	GCTAGGCATGCAAGTATTGTACGCATGGGCCTTAG	SphI
2102	ACATCGATATGCCGGTTATGGAC	
2103	GTTTTCGGATTCTTGTGCTTTAGG	

Supplementary Table 4. Bacterial strains and plasmids used for phenotyping and complementation studies.

Strain or plasmid	Relevant characteristics or phenotype[a]	Reference
Strains		
E. coli		
S17_1 λpir	F^- RP4-2-Tc::Mu *aphA*::Tn7 *recA λpir*; Sm^R	13
Z. galactanivorans		
$Dsij^T$	Wild type	14
mZG_0007	ΔZGAL_3152, obtained from $Dsij^T$	This study
mZG_0008	ΔZGAL_3155, obtained from $Dsij^T$	This study
mZG_0011	ΔZGAL_3159, obtained from $Dsij^T$	This study
mZG_0026	ΔZGAL_3150, obtained from $Dsij^T$	This study
mZG_0031	Wild type (pYT356), obtained from $Dsij^T$; (Em^R)	This study
mZG_0037	ΔZGAL_3155 (pYT356), obtained from mZG_0008; (Em^R)	This study
mZG_0039	ΔZGAL_3155 (pYT360), obtained from mZG_0008; (Em^R)	This study
mZG_0041	ΔZGAL_3159 (pYT356), obtained from mZG_0011; (Em^R)	This study
mZG_0043	ΔZGAL_3159 (pYT361), obtained from mZG_0011; (Em^R)	This study
mZG_0047	ΔZGAL_3150 / ΔZGAL_3152, obtained from mZG_0026	This study
mZG_0054	ΔZGAL_3581-ZGAL_3580, obtained from $Dsij^T$	This study
Plasmids		
pYT313	*sacB*-containing suicide vector; Ap^R (Em^R)	15
pYT354	*sacB*-containing suicide vector; Ap^R (Em^R)	15
pYT356	Suicide vector used for complementation; 2-kbp fragment inside the ZGAL_4579-4596 region fused with *F. johnsoniae ompA* promoter, and cloned into BamHI/XbaI sites of pLYL03; Ap^R Em^R	15
pFT2	2.1-kbp region upstream ZGAL_3150 cloned into PstI and SalI sites of pYT313; Ap^R (Em^R)	This study
pFT5	Construct used to delete ZGAL_3150 in $Dsij^T$; 2-kbp region downstream ZGAL_3150 cloned into SalI and BamHI sites of pFT2; Ap^R (Em^R)	This study
pRF8	2-kbp region upstream ZGAL_3152 cloned into BamHI and SalI sites of pYT313; Ap^R (Em^R)	This study
pRF9	Construct used to delete ZGAL_3152 in $Dsij^T$ and mZG_0026; 2.2-kbp region downstream ZGAL_3152 cloned into SalI and SphI sites of pRF8; Ap^R (Em^R)	This study
pRF10	1.9-kbp region upstream ZGAL_3155 cloned into BamHI and SalI sites of pYT313; Ap^R (Em^R)	This study
pRF11	Construct used to delete ZGAL_3155 in $Dsij^T$; 2.1-kbp region downstream ZGAL_3155 cloned into SalI and PstI sites of pRF10; Ap^R (Em^R)	This study
pRF12	2-kbp region upstream ZGAL_3159 cloned into BamHI and SalI sites of pYT313; Ap^R (Em^R)	This study
pRF13	Construct used to delete ZGAL_3159 in $Dsij^T$; 2.1-kbp region downstream ZGAL_3159 cloned into SalI and PstI sites of pRF12; Ap^R (Em^R)	This study
pYT360	Promoterless ZGAL_3155 cloned into XbaI and SphI sites of pYT356; Ap^R (Em^R)	This study
pYT361	Promoterless ZGAL_3159 cloned into XbaI and SphI sites of pYT356; Ap^R (Em^R)	This study
pYT381	2.1-kbp region upstream ZGAL_3581 cloned into SacI and SpeI sites of pYT354; Ap^R (Em^R)	This study
pYT382	Construct used to delete ZGAL_3581-ZGAL_3580 in $Dsij^T$; 2.2-kbp region downstream ZGAL_3580 cloned into SpeI and SphI sites of pYT381; Ap^R (Em^R)	This study

[a] Antibiotic resistance phenotypes: Ap^R, ampicillin resistance; Em^R, erythromycin resistance. Antibiotic resistance phenotypes without parentheses are expressed in *E. coli*. Antibiotic resistance phenotypes in parentheses are expressed in *Z. galactanivorans* but not *E. coli*.

Supplementary References

1. Ndeh D*, et al.* Complex pectin metabolism by gut bacteria reveals novel catalytic functions. *Nature* 544, 65-70 (2017).

2. Fujita K, Takashi Y, Obuchi E, Kitahara K, Suganuma T. Characterization of a novel beta-L-arabinofuranosidase in *Bifidobacterium longum*: functional elucidation of a DUF1680 protein family member. *J Biol Chem* 289, 5240-5249 (2014).

3. Kiyohara M*, et al.* alpha-N-acetylgalactosaminidase from infant-associated bifidobacteria belonging to novel glycoside hydrolase family 129 is implicated in alternative mucin degradation pathway. *J Biol Chem* 287, 693-700 (2012).

4. Herve C*, et al.* Arabinogalactan proteins have deep roots in eukaryotes: identification of genes and epitopes in brown algae and their role in *Fucus serratus* embryo development. *New Phytol* 209, 1428-1441 (2016).

5. Lacombe JM, Pavia AA, Rocheville JM. New agent of glycosylation - trifluoromethanesulfonic anhydride - synthesis of alpha and beta-O-glycosyl-L-serine, alpha and beta-O-glycosyl-L-threonine and alpha and beta-O-glycosyl-L-hydroxyproline. *Can J Chem* 59, 473-481 (1981).

6. Knutsen S, Myslabodski D, Larsen B, Usov A. A modified system of nomenclature for red algal galactans. *Bot Mar* 37, 163-169 (1994).

7. Kolender AA, Matulewicz MC. Desulfation of sulfated galactans with chlorotrimethylsilane. Characterization of beta-carrageenan by 1H NMR spectroscopy. *Carbohydr Res* 339, 1619-1629 (2004).

8. Zablackis E, Santos GA. The carrageenan of *Catenella nipae* Zanaard., a marine red alga. *Bot Mar* 29, 319-322 (1986).

9. van de Velde F, Pereira L, Rollema HS. The revised NMR chemical shift data of carrageenans. *Carbohydr Res* 339, 2309-2313 (2004).

10. Jouanneau D, Boulenguer P, Mazoyer J, Helbert W. Enzymatic degradation of hybrid iota-/nu-carrageenan by Alteromonas fortis iota-carrageenase. *Carbohydr Res* 345, 934-940 (2010).

11. Michel G*, et al.* The kappa-carrageenase of *P. carrageenovora* features a tunnel-shaped active site: a novel insight in the evolution of Clan-B glycoside hydrolases. *Structure* 9, 513-525 (2001).

12. **Robert X, Gouet P. Deciphering key features in protein structures with the new ENDscript server. *Nucleic Acids Res* 42, W320-324 (2014).**

13. **de Lorenzo V, Timmis KN. Analysis and construction of stable phenotypes in gram-negative bacteria with Tn5- and Tn10-derived minitransposons. *Methods Enzymol* 235, 386-405 (1994).**

14. **Barbeyron T, L'Haridon S, Corre E, Kloareg B, Potin P. *Zobellia galactanovorans* gen. nov., sp. nov., a marine species of *Flavobacteriaceae* isolated from a red alga, and classification of. *Int J Syst Evol Microbiol* 51, 985-997 (2001).**

15. **Zhu Y*, et al.* Genetic analyses unravel the crucial role of a horizontally acquired alginate lyase for brown algal biomass degradation by *Zobellia galactanivorans*. *Environ Microbiol* in the press, (2017).**
