# Supplementary material for: Carrageenan catabolism is encoded by a complex regulon in marine heterotrophic bacteria
Source: Nat Commun. 2017 Nov 22;8:1685. doi: 10.1038/s41467-017-01832-6 (PMC5698469; doi:10.1038/s41467-017-01832-6)
Supplement: Supplementary file 3 — Description of Additional Supplementary Files [file 41467_2017_1832_MOESM3_ESM.pdf]

Description of Additional Supplementary Files

File Name: Supplementary Data 1
Description: Nomenclature for RNA-seq analysis (supplementary data 2-9).

File Name: Supplementary Data 2
Description: List of genes significantly up-regulated in kappa-carrageenan relative to D-galactose growing condition.

File Name: Supplementary Data 3
Description: List of genes significantly up-regulated in iota-carrageenan relative to D-galactose growing condition.

File Name: Supplementary Data 4
Description: List of genes significantly up-regulated in 3,6-anhydro-D-galactose relative to D-galactose growing condition.

File Name: Supplementary Data 5
Description: List of genes significantly down-regulated in kappa-carrageenan relative to D-galactose growing condition.

File Name: Supplementary Data 6
Description: List of genes significantly down-regulated in iota-carrageenan relative to D-galactose growing condition.

File Name: Supplementary Data 7
Description: List of genes significantly down-regulated in 3,6-anhydro-D-galactose relative to D-galactose growing condition.

File Name: Supplementary Data 8
Description: List of all the genes differentially expressed in at least one biological condition (KC, IC or D-AnG relative to D-gal) and sorted by chromosomal position. Genes were
STATION BIOLOGIQUE UMR 8227- Place Georges Teissier - 29682 ROSCOFF Cedex, France
considered differentially expressed with a Family-Wise-Error-Rate controlled at 5% (Bonferroni adjusted p-value <0.05).

File Name: Supplementary Data 9
Description: Full results for all annotated CDS (expression values before and after normalization, statistical analysis results).

File Name: Supplementary Data 10
Description: Limits of the borders for the carrageenolytic PULs considered for analysis for clustering based on sequence similarity. The organism numbering is from the MicroScope Microbial Genome Annotation and Analysis Platform (https://www.genoscope.cns.fr/agc/microscope/home/index.php).

File Name: Supplementary Data 11
Description: : List of the genes that were included in the clustering experiments (Fig. 9)

File Name: Supplementary Data 12
Description: Source file of the GH127 (DagA) phylogenetic tree represented in Supplementary Figure 13 (phylip format).
