# Supplementary material for: Carrageenan catabolism is encoded by a complex regulon in marine heterotrophic bacteria
Source: Nat Commun. 2017 Nov 22;8:1685. doi: 10.1038/s41467-017-01832-6 (PMC5698469; doi:10.1038/s41467-017-01832-6)
Supplement: Supplementary file 2 — Peer Review File [file 41467_2017_1832_MOESM2_ESM.pdf]

Reviewers' comments:

Reviewer #1 (Remarks to the Author):

The paper by Ficko-Blean et al. describes the identification and characterisation of a locus from a marine bacterium Zobelia galactanivorans involved in carrageenan breakdown. Overall while I think the work is of interest and some significance I think the paper is quite hard to follow due mainly to the inconsistent labelling of genes in the text and figures. Furthermore while there is plenty of data here, the characterisation is somewhat incomplete leaves several questions unanswered.

Main points:
1. The authors suggest that they have shown for the first time that a PUL can be composed of distal genes (in this case susC and susD homologues), but there is no experimental evidence that the C and D genes identified are part of the carrageenan PUL. If 3580 and 81 are the SusC-like and SusD-like for CG import there should be some functional data to support this. Either KO one or both genes, or at least show binding of the SusD-like to carrageenan, or ideally both.
2. ZGAL_3637 and 3638 are also a SusC and SusD activated by CG – what do they do? Again ideally a KO or binding tests with the D to test role.
3. Why have 4 enzymes (3 x GH127s and the GH129-like) doing exactly the same thing (page 3)? Later in the paper the authors point out that the GH129-like (3152) is not well conserved in other putative carrageenan utilising species, but this still doesn't answer what the other enzymes doing in Z. galactanivorans? Deletion studies show that 3147 (GH127) is the main enzyme needed for KC and IC breakdown suggesting the others have different roles. The authors should attempt to further characterise these enzymes or at least speculate on what their role may be.
4. Linked to point 3 - why would you need an An-Gal'ase on the cell surface as you propose for the reason why Zg has the GH129-like enzyme (p6, line 3)? This doesn't make sense to me as there is no obvious reason why you would need to remove and An-Gal on the surface. In addition a lipoprotein could be in the OM but facing the periplasm. Some sort of explanation as to why you might need this enzyme on the surface given and ideally cellular location studies should be performed to see where it is. In addition the cellular location of all of the enzymes in the pathway should at least be stated based on signal peptide predictions.
5. Is it not possible to do kinetics with the GH127 and 129-like enzymes vs oligosaccharides to see if there is a preference for different sizes and what the difference in activity is for these apparently biochemically identical enzymes? An-Gal release could be coupled up to conversion to An-galactonate using ZGAL_3155 and monitor reduction of NAD+.
6. You need a B-1,4-galactosidase to fully degrade carrageenan and this is shown as possibly a GH2 on Fig. 2. The authors should look at ZGAL_3633 to see if this enzyme can do this. Otherwise you're missing a key component of carrageenan breakdown ie. with current data you can't fully break it down and in the abstract you say 'complete catabolic pathway' but without the B-gal you have not shown this.
7. Related to this you define a 'core carrageenan utilization system' (p7, L43), but this only has one sulphatase (cgsB) and no b-galactosidase. How do you propose this then is the core system?
8. What do the enzymes in locus ZGAL_3629-34 do if they are activated by carrageenan but are not needed for degradation (except maybe the GH2)? The authors should ideally express and test these enzymes, or at the very least speculate on what they might be doing based on their Cazy family activities and the known structural heterogeneity of CG.
9. The structural data presented is fine, but why is there no attempt to get a substrate (or product) complex to provide insight into specificity? This is mentioned (p5, L14) but no explanation is given as to why this was not attempted. This would be especially relevant/interesting for the GH129-like DagB An-Gal'ase where this activity hasn't been described before.
10. Page 8 Line 25. I don't think it's a good idea to extend the PUL name to other phyla as this is specifically used to describe Bacteroidetes based system which requires SusCD. Other phyla may cluster polysaccharide utilisation genes, but without SusCDs they are not PULS. I think this reclassification is unhelpful and confusing to the field.

11. Importantly - be consistent and clear with names of proteins! Ideally name with locus tag and Cazy family at all times. Naming as dagA3 etc doesn't help – why not call GH127-1 or GH127a? Very confusing. At least name consistently throughout. Also the figures where the enzymes are used should be more clearly labelled with Cazy families e.g. Fig ED1.

Minor points:
12. The GH127s shown on the gels in supplementary data (p7) are very impure compared to the other proteins. Please at least comment on this as it is hard to see if there is any protein present of the correct size with two of the enzymes.
13. Sulfatase specificity should be more clearly stated e.g. in Suppl discussion and Figure 2 ie. ZGAL_3146 is a Gal4S sulfatase etc.
14. In ED Fig 2B what does k/u, l/v and l/a mean in the table? It is not explained and is confusing as there are only 4 carageenans shown in panel A.
15. ED Fig1
a. Panel D. Poor quality figure and hard to see any bands at all. Improve contrast for clarity.
b. Panels E and F - label oligosaccharide alone lanes consistently e.g. for E 'DP6 kappa/beta + empty vector' and for F 'DP6 kappa/beta no enzyme'.
c. Panel F – why do you get different products caused by sulfate loss and HEPES adducts here but not with the GH127s? Is this data just from a single run?
16. Please call these SusC-like and SusD-like rather than SBGP or TBDT in text (e.g. p2, line 32). TBDT is correct but SusC-likes are a specific family of TBDTs.
17. Fig 3 shows only the active site of DagB. It would be better (clearer) to combine this with parts of ED6 (A-D) showing the overall fold and location of active site in context of the whole enzyme. ED6 can then just be the omit map.

Reviewer #2 (Remarks to the Author):

In this article, the authors characterize the catabolic pathway of carrageenans in Z. galactanivorans using biochemical and genetic methods. Their characterization of the pathway for carrageenan utilization includes an atypical polysaccharide utilization locus (PUL) – atypical in the absence of tandem susD-like and susC-like genes. Further, RNA-seq expression profiling is used to evaluate the carrageenan related regulon. Differential gene expression is assayed comparing Z. galactanivorans grown in marine minimal medium with D-galactose (reference) with cells using kappa-carrageenan, iota-carrageenan, or 3,6-anhydro-D-galactose as the unique carbon sources. Three biological replicates are used for each condition. The transcriptome analyses describe carrageenan specific PUL regulation in the assayed conditions, largely consistent with the authors' hypotheses and providing evidence for the extensive carrageenan-induced gene network. The transcriptomic methods are sound and rigorous. The authors also investigate the genomic conservation of the carrageenan utilization system, identifying strong conservation in Bacteroidetes with some weaker conservation in more distant phyla. Altogether, this work describes a model system for carrageenan utilization by marine heterotrophic bacteria.

Reviewer #3 (Remarks to the Author):

This paper describes the genetic and biochemical basis for carrageenan, a major maine (macroalgal). While there have been several studies on glycan degradation by human gut Bacteroidetes, there remains a paucity of information on how the equivalent marine polysaccharides are metabolised. This is important as the recycling of marine polysaccharides has a substantial impact on the bacterial ecology of this substantial and critical ecosystem. Furthermore, many of the enzymes involved in macroalgal metabolism are finding increasing industrial applications.

The data presented here reveal the keystone locus (and orphan genes) involved in carrageenan metabolism. The approach uses extensive biochemical analysis, genetics and some very interesting structural biology. The work clearly represents a substantial advance in our knowledge of an important marine glycan, which differs substantially from the normal structure of terrestrial plant polysaccharides (i.e. the marine glycan comprises a disaccharide backbone, extensive sulfation and a complex anhydro-sugar), and thus recruits a very different repertoire of enzymes to those deployed by human gut bacteria to degrade plant terrestrial polysaccharides. Furthermore the degree and nature of sulfation varies between the different carrageenans. The paper could easily be published unmodified; however, I have a few queries that the authors may wish to consider.

Figure 2: This figure shows the degradation of iota and kappa carrageenan but not lambda, maybe this glycan should be included for completeness. Are the carrageenases on the surface and is their role to generate oligosaccharides of the correct size for import. Maybe Figure 2 could be modified to clarify this issue.

It is suggested that DagA3 is important because it is proposed to be surface located. Why does this make this enzyme particularly important? Is it because the non-reducing terminus of imported oligosaccharides is required to be D-galactose? Maybe this point could be discussed a bit more.

A recent report has shown that GH127 contains an aceric acidase in addition beta-L-arabinofuranosidase. Indeed the enzyme is BT1003 (shown in phylogenetic tree in Data Fig. 5) and contributes to the degradation of the pectin rhamnogalacturonan II (Ndeh et al. (2017 Nature 544, 65-70).

Response to reviewers

Reviewer 1.

Major Points

Queries 1. and 2

"Q1. The authors suggest that they have shown for the first time that a PUL can be composed of distal genes (in this case susC and susD homologues), but there is no experimental evidence that the C and D genes identified are part of the carrageenan PUL. If 3580 and 81 are the SusC-like and SusD-like for CG import there should be some functional data to support this. Either KO one or both genes, or at least show binding of the SusD-like to carrageenan, or ideally both".

"Q2. ZGAL_3637 and 3638 are also a SusC and SusD activated by CG – what do they do? Again ideally a KO or binding tests with the D to test role."

Responses R1 and R2: We cloned the genes encoding the SusD-like protein ZGAL_3580 and ZGAL_3638. We produced and purified these SusD-like proteins (new Supp Fig 1) and tested binding using affinity gel electrophoresis on iota-carrageenan, kappa-carrageenan, lambda-carrageenan, furcellaran, agar and porphyran (new Figure 8). In the native gel without polysaccharide, ZGAL_3580 migrates as a single band, while ZGAL_3638 forms a smear, with at least two major bands. There are changes in the migration profile for ZGAL_3638 on all of the polysaccharides tested, suggesting that the polysaccharides may have an effect on the quaternary structure of the protein. But there is no obvious delay of ZGAL_3638, indicating that this SusD-like protein is unlikely to specifically interact with any of the tested polysaccharides.

In contrast, migration of ZGAL_3580 is retarded (as a clear band) strongly by kappa-carrageenan and furcellaran and moderately by iota carrageenan, no shift is apparent in agar, porphyran or lambda-carrageenan. Therefore, ZGAL_3580 interacts specifically with all the carrageenans except lambda. This is unsurprising, structurally lambda-carrageenan differs from kappa-, iota- and furcellaran as it lacks the 3,6-anhydro-D-galactose residue and in its place has a D-galactose-6-sulfate moiety.

We have also successfully produced a double mutant of *Z. galactanivorans* with the deletion of the *susCD*-like gene pair ZGAL_3580-3581. Phenotyping experiments of this ΔZGAL_3580/ ΔZGAL_3581 mutant demonstrates that bacterial growth is strongly diminished on both kappa- and iota-carrageenan and to a lesser degree on furcellaran. Growth is not affected in Zobell medium, or in minimal medium supplemented by D-galactose or agar. These *in vivo* experiments confirm the importance of this SusCD-like pair to carrageenan utilization by Z. galactanivorans.

Altogether, the biochemical evidence for ZGAL_3580 binding carrageenan, the phenotyping experiments confirming the importance of ZGAL_3580/ZGAL_3581 being implicated in carrageenan utilization, the RNAseq data (Table 1) showing a strong induction of the ZGAL_3580 and ZGAL_3581 genes in both kappa- and iota- carrageenan and finally, the synteny studies showing the conservation of this SusCD-like gene pair within the PULs of other *Bacteroidetes* (Figure 9), support that ZGAL_3580 and ZGAL_3581 form the key outer membrane transport system for the uptake of carrageenan degradation product in *Z. galactanivorans*, and this despite the fact that this susCD-like gene pair is distal from the carrageenan-PUL. The biochemical evidence provided here excludes a significant role of the SusCD-like pair ZGAL_3637-3638 in carrageenan utilization, although its exact specificity remains to be established.

All these new experimental data are described in the new paragraph “Characterization of additional key genes distant from the carrageenan PUL” (page 8 lines 14-36)

Query 3: “Why have 4 enzymes (3 x GH127s and the GH129-like) doing exactly the same thing (page 3)? Later in the paper the authors point out that the GH129-like (3152) is not well conserved in other putative carrageenan utilising species, but this still doesn’t answer what the other enzymes doing in Z. galactanivorans? Deletion studies show that 3147 (GH127) is the main enzyme needed for KC and IC breakdown suggesting the others have different roles. The authors should attempt to further characterise these enzymes or at least speculate on what their role may be.”

Response 3. Our research results (biochemical, combined with growth curves for the *Zobellia galactanivorans* mutants) suggest that the 3 GH127s and the GH129-like do not have exactly the same functions in the bacterium. We speculate that the three GH127 enzymes likely have specificities for different carrageenan motifs. Carrageenans are complex polysaccharides, they may have varying sulfation patterns and other modifications such as methylation or addition of pyruvate groups. In order to determine the fine specificities of these enzymes, the extraction, purification and structural characterization (1H-NMR or/and Mass spectrometry) of natural carrageenans from different algae would be needed as a first step before even characterizing the subtle differences between the 3 GH127s and the GH129-like enzymes. We have here demonstrated unequivocally the novel activity for all these enzymes as 3,6-anhydro-D-galactosidases and while deciphering the fine specificities of these enzymes is important (and work we are continuing) we believe that the detailed studies on the fine specificities of these enzymes belongs in a more specialized journal and is beyond the scope of the present work.

We have nonetheless more developed the discussion about the potential differences between these enzymes:

(page 6 lines 17-21): “Subtle differences between the GH127 and GH129-like enzymes would not be surprising considering the complex, hybrid structure of carrageenans whose regular structures are masked by multiple substituents (e.g. sulfate, methyl, pyruvate). For instance, such variations in substrate specificities are known in GH16 β-agarases and β-porphyranases which act on other red algal sulfated galactans (Hehemann et al, 2012, JBC, 287, 30571-30584).”

Query 4: “Linked to point 3 - why would you need an An-Gal’ase on the cell surface as you propose for the reason why Zg has the GH129-like enzyme (p6, line 3)? This doesn’t make sense to me as there is no obvious reason why you would need to remove and An-Gal on the surface. In addition a lipoprotein could be in the OM but facing the periplasm. Some sort of explanation as to why you might need this enzyme on the surface given and ideally cellular location studies should be performed to see where it is. In addition the cellular location of all of the enzymes in the pathway should at least be stated based on signal peptide predictions.”

Response 4. The predicted cellular location of all the carrageenan-related enzymes has been now predicted using SignalP, Lipop and TMHMM; these predicted localizations have been added to the new Figure 3 (former figure 2). We agree with Reviewer 1 that it is unlikely ZGAL_3150 faces outside releasing D-AnG to the extra-cellular milieu. Thus we have added the following to the text (Page 6, lines 21-25):

"Bioinformatic analyses predict that ZGAL_3150 and ZGAL_3152 are anchored into the outer membrane and secreted in the periplasm, respectively (Fig. 3). The more likely scenario is that ZGAL_3150 is oriented towards the periplasm. Thus, both enzymes would be localized within the periplasm in order to sequentially degrade carrageenan oligosaccharides to produce the monosaccharide D-AnG for uptake by the bacterium."

Query 5. "Is it not possible to do kinetics with the GH127 and 129-like enzymes vs oligosaccharides to see if there is a preference for different sizes and what the difference in activity is for these apparently biochemically identical enzymes? An-Gal release could be coupled up to conversion to An-galactonate using ZGAL_3155 and monitor reduction of NAD+. »

Response 5. We are continuing our studies on the kinetics and fine specificities of these enzymes (see response 3) and believe that the detailed studies on these interesting enzymes belong in a more specialized journal. Determining the fine specificities is not trivial as we need to extract the polysaccharides from different red algae we harvest ourselves, degrade the polysaccharides with the toolbox of marine enzymes we have available in our laboratory and purify the oligosaccharides using different chromatographic methods. Furthermore, the specificity differences are unlikely to be only in the length of the oligosaccharide, but also in the complex sulfation or even methylation or pyruvation patterns on the carrageenan and thus require NMR and Mass Spectrometry to determine the precise substrate/product. We agree with the reviewer that determining the kinetics and fine specificities of these novel enzymes is important and we are continuing to work on this aspect of the project for future publications.

Query 6. "You need a B-1,4-galactosidase to fully degrade carrageenan and this is shown as possibly a GH2 on Fig. 2. The authors should look at ZGAL_3633 to see if this enzyme can do this. Otherwise you're missing a key component of carrageenan breakdown ie. with current data you can't fully break it down and in the abstract you say 'complete catabolic pathway' but without the B-gal you have not shown this."

Response 6. We successfully cloned all the GH2 enzymes from *Z. galactanivorans* (ten constructs in total), including ZGAL_3633. All the soluble GH2 enzymes demonstrated beta-galactosidase activity using the artificial substrate pNP-beta-D-galactopyranoside at pH 5 and pH 7.5. All enzymes had improved catalysis at pH 7.5 compared to pH 5. We prepared odd-DP oligosacarrageenans (presenting a beta-1,4-linked galactose at the non-reducing end) by successively hydrolysing furcellaran (beta/kappa-carrageenan) by the kappa-carrageenase CgkA from *P. carrageenovora* and then by the 3,6-anhydro-D-galactosidase ZGAL_3152 (GH129-like). Using High-Performance Anion-Exchange Chromatography (HPAEC) we have demonstrated that the GH2 ZGAL_3633 and ZGAL_4655 were active on these odd-DP oligo-carrageenans (new Figure 7). Both ZGAL_3633 and ZGAL_4655 are constitutively expressed at low levels in *Z. galactanivorans* in minimum medium supplemented with D-galactose (Supplementary data 2); however, ZGAL_4655 is not induced by kappa- and iota-carrageenans whereas ZGAL_3633 is strongly upregulated and part of the carrageenan regulon, supporting ZGAL_3633 as the key carrageenan-specific beta-galactosidase.

We have described these additional experiments in the new paragraph "Characterization of additional key genes distant from the carrageenan PUL" (from page 7 line 39 to page 8 line 9). We have also added the following text to reflect that we have identified all the enzymes activities necessary for the complete degradation of carrageenan by *Z. galactanivorans* (Page 8 lines 9-13): "Thus, *Z. galactanivorans* has all the enzyme activities necessary for the complete degradation of kappa family carrageenans, most of them encoded by the ZGAL_3145-3159 cluster and the others by remote, carrageenan-induced genes (GH16: ZGAL_236; GH82: ZGAL_1973, ZGAL_4265; GH2: ZGAL_3633; S1-7: ZGAL_181).".

Query 7: "7. Related to this you define a 'core carrageenan utilization system' (p7, L43), but this only has one sulphatase (cgsB) and no b-galactosidase. How do you propose this then is the core system?"

Response 7. The use of the phrase 'core carrageenan utilization system' does not necessarily mean 'complete carrageenan utilization system'; it refers to the shared common carrageenan utilization enzymes between the bacteria. This precision has been added in the text (page 10, lines 22-24). Regarding the absence of the sulfatases for iota-carrageenan suggests the organisms are only able to use kappa-carrageenan or that the organism has accrued a different sulfatase that complements the activity. In some cases the bacteria are lacking a known iota-carrageenase and a kappa-carrageenase, again it's possible that other as yet unidentified enzymes are capable of acting as kappases or iotases. Such an example is found with cellulases, where several different CAZy families have evolved cellulolytic activities. Another possibility is that in the bacteria's niche, other organisms secrete the kappases or iotases necessary for initial degradation of carrageenan outside the periplasm.

Query 8. "What do the enzymes in locus ZGAL_3629-34 do if they are activated by carrageenan but are not needed for degradation (except maybe the GH2)? The authors should ideally express and test these enzymes, or at the very least speculate on what they might be doing based on their Cazy family activities and the known structural heterogeneity of CG".

Response 8. Many genes/regions of *Z. galactanivorans* have been upregulated by carrageenan and all of these remain potential targets for characterization. In many cases the upregulated genes are obviously not implicated in carrageenan degradation, for example the glycosyl transferases GT2 (ZGAL_2991) and GT4 (ZGAL_2990) (See Supplementary Data 2). In the additional experiments requested by Reviewer 1, we demonstrated that the GH2 ZGAL_3633 is indeed a beta-galactosidase active on odd-DP-carrageenan (new Figure 7). Proximal to the ZGAL_3629-34 region is the SusCD-like pair ZGAL_3637/ZGAL_3638. We overproduced the SusD-like protein ZGAL_3638 and demonstrated it does not specifically bind to carrageenan (new Figure 8). Examination of the nearby gene context reveals two predicted GH16 beta-porphyranases: ZGAL_3628 (PorD) and ZGAL_3640 (PorE). Both of these beta-porphyranase genes were also highly induced in iota- and kappa-carrageenan. However beta-porphyranases are not active on carrageenans (Hehemann et al, Nature, 2010; Hehemann et al, JBC, 2012). We did not include them in Table 1 because the genes are not found in synteny with other carrageenan-degrading marine bacteria. To summarize, we have provided additional experimental data on the genetic region ZGAL_3629-3638. Although ZGAL_3633 is a beta-galactosidase active on carrageenans, the SusD-like ZGAL_3638 does not interact specifically with carrageenans. One hypothesis is that ZGAL_3633 may have a broader specificity and could also be active on other galactans. Considering the presence of nearby beta-porphyranases, this cluster is more likely dedicated to the degradation of another sulfated galactan from red algae, distinct from carrageenans (e.g. porphyrans or another sulphated agar).

We have added a sentence to discuss the potential function of the locus ZGAL_3628-3640 (page 8, lines 24-28): "The absence of ZGAL_3638 specificity for carrageenans and the presence of two putative GH16 beta-porphyranases, ZGAL_3628 (PorD) and ZGAL_3640 (PorE) encoded in the ZGAL_3628-3640 locus suggest that this locus is more likely dedicated to the degradation of another sulfated galactan distinct from carrageenans (e.g. sulfated agars)."

Query 9. "The structural data presented is fine, but why is there no attempt to get a substrate (or product) complex to provide insight into specificity? This is mentioned (p5, L14) but no explanation is given as to why this was not attempted. This would be especially relevant/interesting for the GH129-like DagB An-Gal'ase where this activity hasn't been described before"

Response 9. We have tried several times to get a complex by soaking the crystals with just D-AnG alone and by soaking attempts with several different oligosaccharides that we produced and purified chromatographically ourselves. We have also tried soaks with the oligosaccharides to try and get a Michaelis-complex with 3 different GH129-like active site mutants. We have not been fortunate enough to obtain a complex at this time; these experiments are on-going. We have added this sentence in the text to state this situation (page 5, lines 13-15): "Numerous trials for obtaining the structure of ZGAL_3152 complexes were performed, but they were unsuccessful."

Query 10. "Page 8 Line 25. I don't think it's a good idea to extend the PUL name to other phyla as this is specifically used to describe Bacteroidetes based system which requires SusCD. Other phyla may cluster polysaccharide utilisation genes, but without SusCDs they are not PULS. I think this reclassification is unhelpful and confusing to the field."

Response 10. We disagree with Reviewer 1's position to restrict the use of the PUL name solely based on the presence of the SusCD gene pair and thus to the Bacteroidetes for several reasons:

(1) Thanks to Reviewer 1's incitation to characterize our susCD candidates, we have now demonstrated experimentally, both biochemically and with a knock out mutant, that *Z. galactanivorans* has a carrageenan-specific *susCD* gene pair (ZGAL_3580-3581) which is not localized in the main cluster containing the genes responsible for the utilization of carrageenan. In our bioinformatics analysis (new figure 9, former figure 5), we show that other *Bacteroidetes* have carrageenan-specific PUL which contains ZGAL_3580-3581 orthologous genes. In this context, it won't make sense to not refer to the ZGAL_3145-3159 cluster as a PUL, on the sole basis that the *susCD* gene pair is distal from the main cluster. The demonstration of a functional *susCD* gene pair distal from the PUL, but induced as part of the carrageenan regulon, is now a main general take-home message from our work.

(2) Our bioinformatics analysis also shows that other bacterial phyla have similar carrageenan-specific gene clusters (with relatively good conservation in gene composition and synteny), less the *susCD-like* gene pair. For unknown reason *susD*-like genes are unique to the *Bacteroidetes* phylum. But it does not mean that TonB-dependent receptors are absent in polysaccharide-related gene clusters of other phyla. For instance, all clade 3 Gammaprotobacteria species (Fig. 9) have a TonB-dependent receptor gene in their carrageenan-related cluster (CATDS2_v1220055 in *Catenovulum agarivorans* DS-2; JRKG01_v1_110122 in *Pseudoalteromonas* sp. PLSV; Patl_0887 in *P. atlantica* T6c; H978DRAFT_1909 in *Alteromonas* sp. ALT199). They are distantly related to the carrageenan-specific TonB-dependent receptor ZGAL_3581, but their location within the cluster strongly suggests that they play a similar role in the import of carrageenan degradation products (we have now added these information in the text, from page 9 , line 44 to page 10, line 5). Such clusters containing a

TonB-dependent receptor gene and dedicated to the use of polysaccharides have been already described in *Gammaproteobacteria* ten year ago (Blanvillain et al, Plant Carbohydrate Scavenging through TonB-Dependent Receptors: A Feature Shared by Phytopathogenic and Aquatic Bacteria 2007, PLOSone, 2, e224). The authors have named these gene clusters “Carbohydrate Utilization Loci” (CUTL), but visibly this term has gained much less popularity than the term PUL. In our opinion, these loci are fundamentally similar: (i) they contain genes dedicated to the use of polysaccharides; (ii) they contain TonB-dependent receptor for the transport of the degradation products; (iii) they are exchanged by horizontal gene transfers and thus have often common evolutionary origin. Since these biological objects are similar, we believe it is more important to standardize the name of these loci (using the more popular term PUL) than to maintain distinct nomenclatures which tends to separate communities (e.g. gut microbiota versus plant phytopathologist) that in fact work on equivalent issues in glycobiology.

(3). Finally, the formal proposition of the extension of the PUL nomenclature, strengthened by the experimental and bioinformatics results presented here, is very timely, because things are moving quickly in the field. Recently a review article has also put forward the reflection that PULs are found in organisms other than *Bacteroidetes* (Grondin et al, 2017, Polysaccharide Utilization Loci: Fuelling microbial communities. J. Bacteriol., doi:10.1128/JB.00860-16). References can also be found in the literature in which the authors are already using the PUL term for describing gene clusters dedicated to polysaccharide utilization in organisms that are not *Bacteroidetes* (e.g. Sheridan et al, 2016, Polysaccharide utilization loci and nutritional specialization in a dominant group of butyrate-producing human colonic *Firmicutes*. Microb Genom. 2:e000043; Zhu et al. Complete genome sequence and transcriptomic analysis of a novel marine strain *Bacillus weihaiensis* reveals the mechanism of brown algae degradation. (2016) Scientific Reports ; 6:38248; Ulaganathan et al, 2017, New Ulvan-Degrading Polysaccharide Lyase Family: Structure and Catalytic Mechanism Suggests Convergent Evolution of Active Site Architecture. ACS Chem Biol. 12(5):1269-1280). Thus, even in the literature there is a natural convergence to name genetic regions dedicated to the utilisation of polysaccharides as PULs.

For all these reasons, we have decided to maintain our proposition of extending the PUL nomenclature.

Query 11: “Importantly - be consistent and clear with names of proteins! Ideally name with locus tag and Cazy family at all times. Naming as dagA3 etc doesn’t help – why not call GH127-1 or GH127a? Very confusing. At least name consistently throughout. Also the figures where the enzymes are used should be more clearly labelled with Cazy families e.g. Fig ED1.”

Response 11. We standardized the naming so that the name referenced in the text conforms to the locus tag as asked (e.g. ZGAL_3152). Where appropriate we have also indicated the CAZy numbering (e.g. GH127-1, GH127-2, GH127-3) and the enzyme name (e.g. 3,6-anhydro-D-galactose dehydrogenase) as we realize that with all the different nomenclatures for the proteins it is easy to lose track. We have also added an explication of the gene naming in Figure 1 and new Figure 9 (former Figure 5) for clarity.

Minor Points

Query 12: “The GH127s shown on the gels in supplementary data (p7) are very impure compared to the other proteins. Please at least comment on this as it is hard to see if there is any protein present of the correct size with two of the enzymes.”

Response 12. Only ZGAL_3147 is very impure. The other GH127 enzymes were quite pure after purification by IMAC. We acknowledge the impurity of ZGAL_3147; however, we were still able to demonstrate activity as a 3,6-anhydro-D-galactosidase (an activity *E. coli* doesn’t have), therefore this has reassured us that the enzyme is present solution.

Query 13. “Sulfatase specificity should be more clearly stated e.g. in Suppl discussion and Figure 2 ie. ZGAL_3146 is a Gal4S sulfatase etc.”

Response 13. We have changed the supplementary section on the sulfatases to clearly indicate the specificities. We have also updated new Figure 3 (former figure 2) to reflect the nomenclature.

Query 14. “In ED Fig 2B what does k/u, l/v and l/a mean in the table? It is not explained and is confusing as there are only 4 carageenans shown in panel A.”

Response 14. We have updated the representations of the carrageenan disaccharide structures to reflect all the different carrageenans mentioned within the table.

Query 15. “5. ED Fig1 a. Panel D. Poor quality figure and hard to see any bands at all. Improve contrast for clarity. b. Panels E and F - label oligosaccharide alone lanes consistently e.g. for E ‘DP6 kappa/beta + empty vector’ and for F ‘DP6 kappa/beta no enzyme’. c. Panel F – why do you get different products caused by sulfate loss and HEPES adducts here but not with the GH127s? Is this data just from a single run?”

Response 15: We have updated the former Extended Data Fig. 1 (new figure 2) to answer Reviewer 1’s critics:

a) Panel D- we have improved contrast for clarity.

b) The labels have been changed for clarity.

c) This data is not from a single run, therefore the buffer compositions vary between the GH127 enzymes and the GH129 enzyme. The GH129 enzyme was stored in HEPES and therefore we see HEPES adducts in the GH129 mass spectrum.

Query 16. “Please call these SusC-like and SusD-like rather than SBGP or TBDT in text (e.g. p2, line 32). TBDT is correct but SusC-likes are a specific family of TBDTs.”

Response 16. We now use the term “SusC-like” and “SusD-like” throughout the text and figures. We have suppressed the term SBGP. We have conserved the term TBDT, but just as an abbreviation of TonB-dependent transporter.

Query 17. “Fig 3 shows only the active site of DagB. It would be better (clearer) to combine this with parts of ED6 (A-D) showing the overall fold and location of active site in context of the whole enzyme. ED6 can then just be the omit map.”

Response 17. We have merged the active site of DagB with the figure ED6 to be included as a single figure in the main text (new figure 4).

Reviewer 2: no query.

Reviewer 3.

Query 1. "Figure 2: This figure shows the degradation of iota and kappa carrageenan but not lambda, maybe this glycan should be included for completeness. Are the carrageenases on the surface and is their role to generate oligosaccharides of the correct size for import. Maybe Figure 2 could be modified to clarify this issue."

Response 1: The reason we did not include lambda-carrageenan is that its structure is different from that of kappa- and iota-carrageenan. Kappa- and iota-carrageenan have the repeating alpha 1,3-linked disaccharide unit [D-galactose-beta(1,4)-3,6-Anhydro-D-galactose]; Lambda-carrageenan has the repeating alpha 1,3-linked disaccharide unit [D-galactose-beta(1,4)-D-galactose-6-sulfate]. Thus, lambda-carrageenan does not contain 3,6-Anhydro-D-galactose and its degradation requires a different set of enzymes which remain to be identified. Therefore, we have changed our text to summarize this (page 2, lines 43-46).

"Here we describe in *Z. galactanivorans* the discovery and the integrative characterization of the complete pathway for utilization of the kappa family carrageenans (containing 3,6-anhydro-D-galactose units, mainly kappa-, iota- and beta-carrageenans; Craigie, 1990. Cell Walls. In: Biology of the red algae. Cambridge University Press),…."

We have modified the new figure 3 (former Fig. 2) to show the predicted localizations of the proteins within the kappa- and iota-carrageenan degradation pathway. The kappa- (ZGAL_236) and iota- (ZGAL_4265) carrageenases have been previously demonstrated experimentally to be extracellular. Thus, we have add the following text (page 7, lines 21-24): "The wild-type kappa-carrageenase CgkA and iota-carrageenase CgiA1 were previously shown to be extracellular enzymes (Barbeyron et al, 1998, Mol Biol Evol; Barbeyron et al, 2000, J Biol Chem). Thus, the role of these enzymes is probably to generate oligosaccharides for transport by *Z. galactanivorans*'s SusCD-like transport system."

Query 2. "It is suggested that DagA3 is important because it is proposed to be surface located. Why does this make this enzyme particularly important? Is it because the non-reducing terminus of imported oligosaccharides is required to be D-galactose? Maybe this point could be discussed a bit more."

Response 2: RNAseq data shows the induction of stress-related genes when *Z. galactanivorans* is grown on just D-AnG, this suggests that there is no dedicated outer membrane transport system for D-AnG. We find it unlikely that the outer-membrane DagA3 (GH127-3) is oriented toward the extracellular medium if there is no dedicated outer membrane transporter for import of D-AnG (see response 4 to Reviewer 1); however, we cannot entirely discount the possibility. Therefore we have changed the text to the following (page 6, lines 15-25):

“We attribute the difference in deletion effect may be due to either an unknown difference in specificity between the enzymes or different cellular localizations of the enzymes. … Bioinformatic analyses predict that ZGAL_3150 and ZGAL_3152 are anchored into the outer membrane and secreted in the periplasm, respectively (Fig. 3). The more likely scenario is that ZGAL_3150 is oriented towards the periplasm. Thus, both enzymes would be localized within the periplasm in order to sequentially degrade carrageenan oligosaccharides to produce the monosaccharide D-AnG for uptake by the bacterium.”

Query 3: “A recent report has shown that GH127 contains an aceric acidase in addition beta-L-arabinofuranosidase. Indeed the enzyme is BT1003 (shown in phylogenetic tree in Data Fig. 5) and contributes to the degradation of the pectin rhamnogalacturonan II (Ndeh et al. (2017 Nature 544, 65-70).”

Response 3: We have updated the text to include the GH127 BT1003 activity and to cite the corresponding article (page 3, lines 15-17):

“The GHs have distant homologues in enteric bacteria: BT1003 (BtGH127) from *Bacteroides thetaiotaomicron* is a 3-C-carboxy-5-deoxy-L-xylose (aceric acid)-hydrolase involved in rhamnogalacturonan-II depolymerization 24; …”

REVIEWERS' COMMENTS:

Reviewer #1 (Remarks to the Author):

Overall I am satisfied that the authors have addressed most of my comments in a detailed and careful manner and I am happy to recommend publication of the revised MS.

I have only a couple of additional comments regarding the interpretation of new SusCD-like data shown in Fig 8:

1. In the main text (line 326) the authors state that 'In contrast, migration of ZGAL_3580 is retarded (as a clear band) strongly by kappa-carrageenan and furcellaran and moderately by iota carrageenan.'
I would argue that the retardation observed is only small, and so the term 'strongly' should not be used. Instead the text should read something like 'migration of ZGAL_3580 SusD-like is retarded by kappa-carrageenan and furcellaran, suggesting the protein interacts specifically with these polymers.'
In addition the shift seen with IC is so small as to be hard to interpret – it could be that just gel to gel variation rather than a specific interaction. The fact that this is stated to be a 'moderate retardation' should also be toned down.

2. Surprisingly the defect seen with the CD deletion strain is most obvious on IC and much less so on furcellaran (Fig 8B). Can the authors speculate on why this might be?

Minor point:
I think when talking about PULs that target marine polysaccharides the paper by Hehemann et al PNAS (2012) 109:19786-91 (PMID 23150581) should be cited somewhere (probably Intro) as this the first time that components of a PUL targeting a marine PS (porphyran) was characterised.

Reviewer #3 (Remarks to the Author):

N/A

Response to reviewers

Reviewer 1

"Overall I am satisfied that the authors have addressed most of my comments in a detailed and careful manner and I am happy to recommend publication of the revised MS.

I have only a couple of additional comments regarding the interpretation of new SusCD-like data shown in Fig 8:"

Query 1: "In the main text (line 326) the authors state that 'In contrast, migration of ZGAL_3580 is retarded (as a clear band) strongly by kappa-carrageenan and furcellaran and moderately by iota carrageenan.' I would argue that the retardation observed is only small, and so the term 'strongly' should not be used. Instead the text should read something like 'migration of ZGAL_3580 SusD-like is retarded by kappa-carrageenan and furcellaran, suggesting the protein interacts specifically with these polymers.' In addition the shift seen with IC is so small as to be hard to interpret – it could be that just gel to gel variation rather than a specific interaction. The fact that this is stated to be a 'moderate retardation' should also be toned down."

Answer 1. We have changed this sentence as requested by Reviewer 1: (page 8, lines 38-39): "In contrast, migration of ZGAL_3580 is retarded (as a clear band) by kappa-carrageenan and furcellaran and only slightly by iota carrageenan."

Query 2. "Surprisingly the defect seen with the CD deletion strain is most obvious on IC and much less so on furcellaran (Fig 8B). Can the authors speculate on why this might be?"

Answer 2. We have modified the paragraph to give a plausible explanation to the differences between the biochemical and genetic approaches (from Page 8, line 41 to page 9, line 3).

"Phenotyping experiments on *Z. galactanivorans* deletion mutant Δ*zgal_3580*/Δ*zgal_3581* indicate pronounced inhibition of growth relative to wild type on both KC and IC and moderate inhibition of growth on furcellaran (Fig. 8B). The biochemical (Fig. 8A) and the genetic (Fig. 8B) experiments confirm the affinity of ZGAL_3580 and ZGAL_3581 for family kappa carrageenans, but with a difference in preference depending on the chosen method. However, the genetic approach evaluates the combined properties of ZGAL_3580 and ZGAL_3581, whereas the gel shift assay only characterized ZGAL_3580. This suggests that the TBDT ZGAL_3581 has a strong affinity for iota-carrageenan, which compensates for the reduced affinity of the SusD-like protein ZGAL_3580 for this polysaccharide relative to KC."

Query 3. "Minor point: I think when talking about PULs that target marine polysaccharides the paper by Hehemann et al PNAS (2012) 109:19786-91 (PMID 23150581) should be cited somewhere (probably Intro) as this the first time that components of a PUL targeting a marine PS (porphyran) was characterised."

Answer 3. Before our current work, there were indeed some examples of partial characterization of PULs targeting marine polysaccharides, but not at an integrative level as observed in the field of PULs from human gut *Bacteroidetes*. We nonetheless agree to cite this reference alongside another such an example. We have added the following paragraph (from page 2, line 40 to page 3, line 2):

“Some previous studies have described the partial characterization of PULs targeting marine polysaccharides, for instance, alginate-specific PULs were shown to be genuine operons and to encode the enzymes responsible for the bioconversion of alginate into 2-keto-3deoxy-6-phosphogluconate (Thomas et al, 2012, Env Micobiol). The transcription of a PUL in *Bacteroides plebeius* was shown to be induced by porphyran. The PUL-encoded enzymes responsible for the initial endolytic degradation of this sulfated agar (family GH16 and GH86 β-porphyranases) were biochemically and structurally characterized (Hehemann et al, 2012, PNAS). However, such studies, focusing on macroalgal polysaccharides, have not yet reached the level of integrative characterization observed in the field of PULs from human gut *Bacteroidetes*.”
